# Functional biosynthetic stereodivergence in a gene cluster via a dihydrosydnone *N-*oxide

**DOI:** 10.1038/s42004-024-01372-3

**Published:** 2024-12-19

**Authors:** Jiajun Ren, Anugraha Mathew, María Rodríguez-García, Tobias Kohler, Olivier Blacque, Anthony Linden, Leo Eberl, Simon Sieber, Karl Gademann

**Affiliations:** 1 Department of Chemistry, University of Zurich, Zurich, Switzerland; 2 Department of Plant and Microbial Biology, University of Zurich, Zurich, Switzerland

**Keywords:** Biosynthesis, Biocatalysis, Metabolic pathways

## Abstract

Chirality plays a critical role in the biochemistry of life and often only one enantiomeric series is observed (homochirality). Only a few natural products have been obtained as racemates, e.g. the signalling molecule valdiazen produced by *Burkholderia cenocepacia* H111. In this study, we investigated the *ham* biosynthetic gene cluster and discovered that both the enantiomerically pure (*R*)-fragin and the racemic valdiazen result from the same pathway. This stereodivergence is based on the unusual heterocyclic intermediate dihydrosydnone *N*-oxide, as evident from gene knockout, stable isotope feeding experiments, and mass spectrometry experiments. Both non-enzymatic racemisation via keto-enol tautomerisation and enzyme-mediated dynamic kinetic resolution were found to be crucial to this stereodivergent pathway. This novel mechanism underpins the production of configurationally and biologically distinct metabolites from a single gene cluster. Our findings highlight the intricate design of an intertwined biosynthetic pathway and provide a deeper understanding of microbial secondary metabolism related to microbial communication.

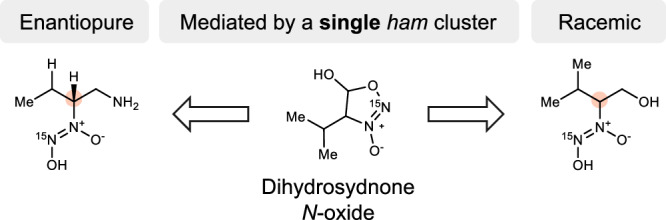

## Abstract (web summary)

The *ham* gene cluster in *Burkholderia cenocepacia* H111 produces two compounds: the signalling molecule valdiazen as racemate and the antifungal fragin in enantiopure form. Here, the authors demonstrate that the stereodivergence is based on the heterocyclic intermediate dihydrosydnone *N*-oxide, with both non-enzymatic racemization and enzyme-mediated dynamic kinetic resolution occurring in parallel.

## Introduction

**Fig. 1:**
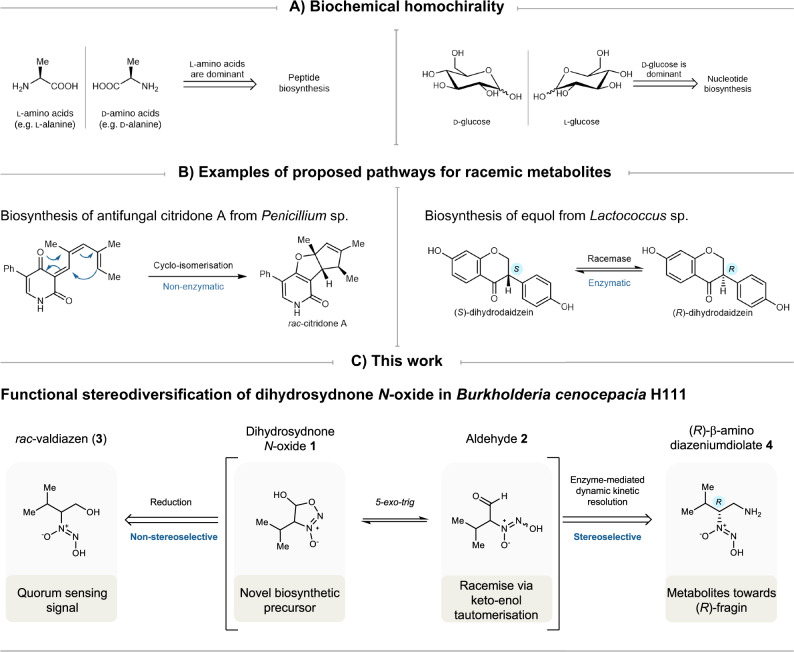
Homochirality and racemisation in natural products. **A** Homochiral preferences in the biosynthesis of peptides and nucleotides^1^. **B** Two proposed biosynthetic pathways resulting in racemic metabolites^9,10^. **C** Dihydrosydnone *N*-oxide is a key intermediate at the junction of the biosynthesis of valdiazen and fragin in *Burkholderia cenocepacia* H111.

Homochirality describes the dominance of one enantiomer over the other across the entire biosphere on Earth. For instance, the primary metabolism of living organisms relies almost exclusively on l-amino acids to make proteins, while using right-handed carbohydrates (d-monosaccharides) to make polysaccharides and DNA (Fig. 1a)^1^. Although the origin of biochemical homochirality remains controversial^2^, building macromolecules with one enantiomer eliminates the diastereoisomer problem^3^, and renders biological processes more efficient^4–7^. However, racemic secondary metabolites are sometimes produced in a homochiral biological environment, in particular regarding alkaloids and terpenoids^8^. Different racemisation mechanisms have been documented, including both enzymatic and non-enzymatic pathways^8^. The dihydrodaidzein racemase involved in the biosynthesis of equol is an example of enzymatic pathways^9^. Non-enzymatic pathways typically involve a reactive biosynthetic intermediate that can undergo dimerisation, cyclisation, or tautomerisation (Fig. 1b)^8,10^. In this study, we demonstrate that both non-enzymatic racemisation and enzyme-mediated dynamic kinetic resolution occur in parallel and are encoded by the *ham* gene cluster in *Burkholderia cenocepacia* H111^11^.

The *ham* cluster is rather unique in producing two secondary metabolites with different stereogenic configurations and distinct biological functions, in contrast to other diazeniumdiolate gene clusters^12–20^. The antifungal metabolite (–)-fragin features the (*R*)-configuration opposite to that of its proteinogenic (*S*)-configured l-valine precursor, whereas the signalling molecule valdiazen was found as a racemate^11^. This suggests that racemisation and dynamic kinetic resolution occur in the biosynthesis of these two molecules. Moreover, valdiazen regulates the production of fragin and autoregulates its own production via a positive feedback loop, i.e. valdiazen induces its own and fragin biosynthesis^11^. In this study, we provide evidence that the different configurations of secondary metabolites produced by the *ham* cluster might be the result of a finely tuned interplay between enol-keto tautomerisation and enzyme-mediated dynamic kinetic resolution, centred around the novel heterocycle dihydrosydnone *N*-oxide **1** (Fig. 1c). Specifically, we hypothesise the formation of aldehyde **2**, which can easily racemise due to keto-enol-transformation and conversion to dihydrosydnone *N*-oxide **1**. Further downstream processes lead either to racemic valdiazen (**3**) or to (*R*)-fragin (**5**) via enantiomerically pure amine **4**. In this study, we provide compelling evidence supporting these intermediates in this pathway and their stereochemical fate.

## Results and Discussion

### Evidence for a dihydrosydnone *N*-oxide as a biosynthetic intermediate

**Fig. 2:**
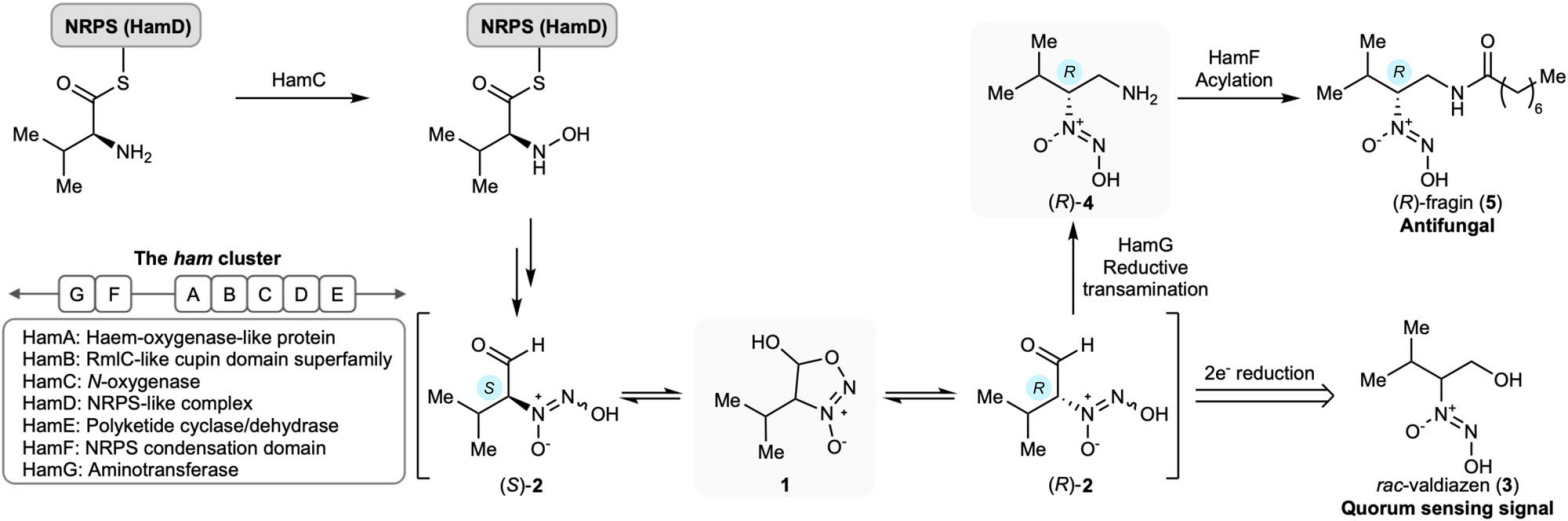
Proposed biosynthesis of (*R*)-fragin (5) and *rac*-valdiazen (3) via dihydrosydnone *N*-oxide 1 mediated by the *ham* cluster in *B. cenocepacia* H111. In this figure, the proposed mechanism of racemisation is represented and includes the formation of the dihydrosydnone *N*-oxide **1** intermediate.

We hypothesised the existence of a common aldehyde intermediate **2** (Fig. 2) for the *ham*-mediated biogenesis of (*R*)-fragin (**5**) and *rac*-valdiazen (**3**), based on previous findings from our group and others^11,13–16,19–26^. The *ham* cluster consists of seven genes distributed in two oppositely oriented operons^11,26^. Previous bioinformatic analyses predicted that operon *hamABCDE* encodes a haem-containing oxygenase (*hamA*), a cupin domain-containing protein (*hamB*), an *N-*oxygenase (*hamC*), a non-ribosomal peptide synthetase (NRPS)-like protein complex (*hamD*), and a polyketide cyclase or dehydrase (*hamE*)^11^. From the second operon, *hamF* was predicted to encode a starter condensation domain, while *hamG* was suggested to be an aminotransferase^11^.

As demonstrated by isotopically labelled feeding experiments, the *ham*-mediated biosynthesis starts with loading l-valine onto the HamD NRPS complex^26^. The tethered l-valine is oxidised by *N*-oxygenase HamC^11,23,27^, which shares homology with diiron *N*-oxygenase AurF^28^. In addition, the cupin domain-containing HamB was suggested non-essential for valdiazen production^11^, and that HamA (PvfA) and HamE (PvfD) were non-essential for the production of hydroxylamine intermediates towards valdiazen and pyrazine *N*-oxide derivatives^22^. However, enzymes responsible for diazeniumdiolate formation and two-electron reduction converting aldehyde **2** to valdiazen (**3**) remain elusive. It was hypothesised that the reductase domain of HamD NRPS could mediate the two-electron reduction^11^.

The NRPS HamD contains a reductase domain instead of the more typical thioesterase domain. A similar NRPS architecture leading to an aldehyde intermediate was discovered in the biosynthesis of myxochelins^21,29^ and myxalamid S^30^. The C(α)-proton in aldehyde **2** is expected to have increased acidity due to the presence of both electron-withdrawing aldehyde and diazeniumdiolate functional groups. From the second operon, both HamF and HamG are indispensable for the production of fragin^11^. In addition, we reason that either the aminotransferase HamG or the condensation domain HamF will determine the configuration of fragin. Consequently, tautomerisation and racemisation of such an aldehyde could be central for the stereodivergence mediated by this biosynthetic gene cluster.

**Fig. 3:**
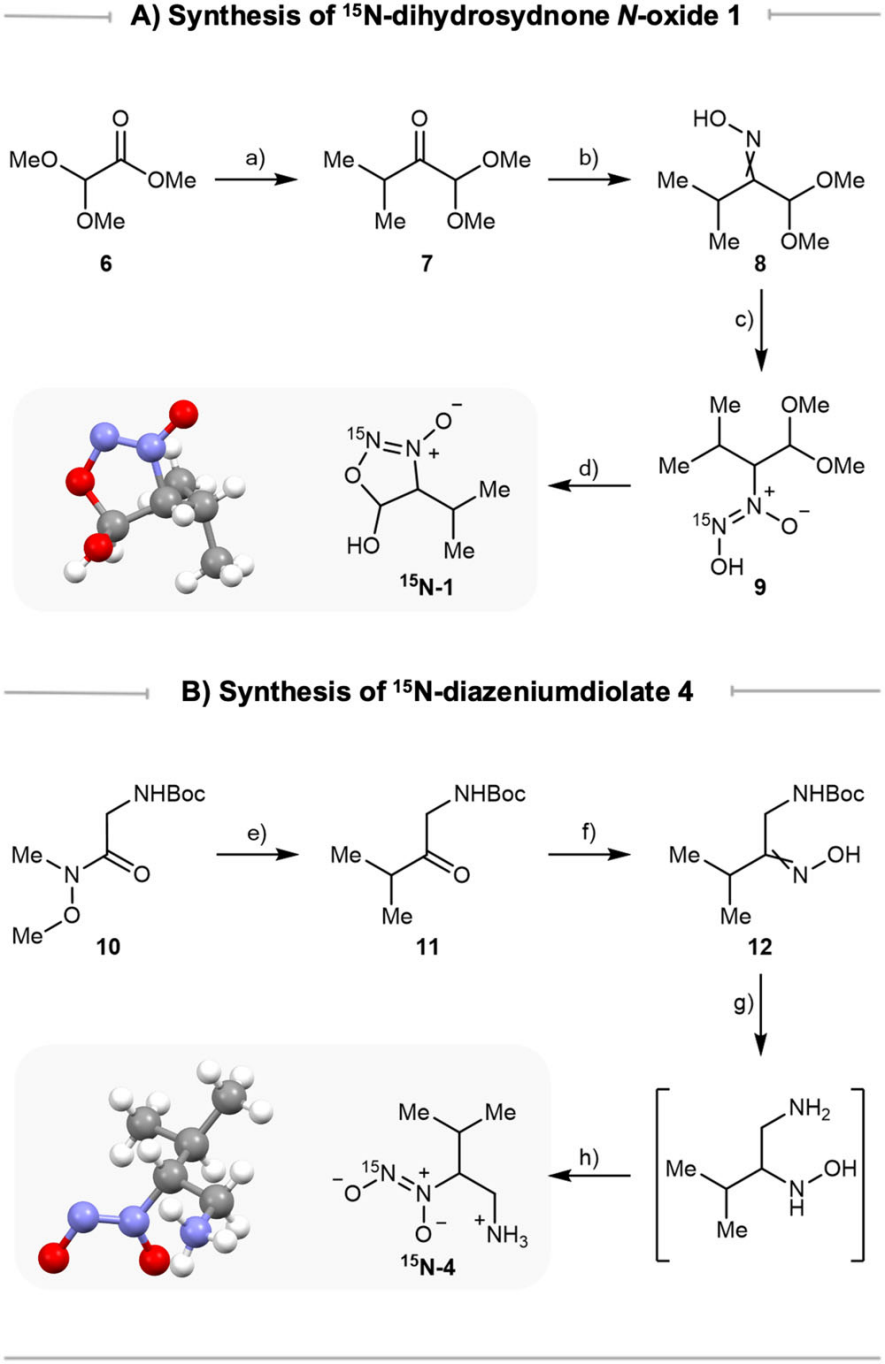
Synthesis of ^15^N-labelled tracers and the solid-state molecular structures of unlabelled compounds 1 and 4. Colour code: blue = nitrogen; red = oxygen; grey = carbon; white = hydrogen. **A** Synthesis of ^15^N-dihydrosydnone *N*-oxide **1**: a) AlMe_3_, *N*-methoxy-methanamine·HCl, 4.5 h, then isopropyl magnesium bromide, 2 h, 56% over 2 steps; b) NH_2_OH·HCl, NaOAc, EtOH/H_2_O, 70 °C, 3 h, 81%; c) NaBH_3_CN, HCl, EtOH, 1 h, then 1 M aq. HCl, Na^15^NO_2_, 1 h, 55% over 2 steps; d) 10% aq. HCl, 50 °C, 3 h, 46%. **B** Synthesis of ^15^N-diazeniumdiolate **4**: e) isopropyl magnesium bromide, 2 h, 49%; f) NH_2_OH·HCl, KOAc, EtOH/H_2_O, 70 °C, 3 h, 89%; g) NaBH_3_CN, HCl, EtOH/H_2_O, 1 h, then TFA, DCM, 1 h; h) 1 M aq. HCl, Na^15^NO_2_, 30 mins, 21% over 3 steps.

In order to address these hypotheses, we first synthesised standards and ^15^N-labelled tracers for feeding experiments and mass spectrometry analyses. Methyl 2,2-dimethoxyacetate **6** was transformed to ketone **7** via the respective Weinreb amide (Fig. 3a). Ketone **7** was then reacted with hydroxylamine to oxime **8** as a mixture of (*E*/*Z*)-isomers in 81% yield. The oximes were further reduced and nitrosated using ^15^N-sodium nitrite to give ^15^N-diazeniumdiolate acetal **9**. Subsequent acid hydrolysis at 50 °C did not lead to aldehyde **2**, but instead to hemiacetal ^15^N-**1**. This novel heterocycle dihydrosydnone *N*-oxide **1** was fully characterised, including by ^1^H-NMR spectroscopy (characteristic doublet at 5.95 ppm in methanol-*d*_*4*_), and its structure was unambiguously assigned by X-ray crystal structure analysis (Supplementary Table 1 and Supplementary Data 1). Interestingly, only the *trans*-diastereoisomer of racemate **1** was observed in the solid state and in solution, as evidenced by ^1^H-NMR spectroscopy. The heteroatom-rich molecular architecture of **1** constitutes the first example of dihydrosydnone *N*-oxides. The corresponding oxidised form, sydnone *N*-oxide (Traube’s anion), was discovered in 1895^31^. Since then, only a few derivatives of this aromatic family were synthesised mostly using NO gas^31–36^. Notably, molsidomine containing a sydnone imine core has been approved as a vasodilating drug for angina pectoris^37,38^. Other similar heterocycles including 4,5-dihydro-1,2,3-oxadiazole^39^, and 5-hydroxy-3-methyloxadiazolinium perchlorate have been synthesised^40^.

The ^15^N-labelled amine analogue **4** was synthesised to interrogate the role of the predicted condensation domain HamF. The commercially available Weinreb amide **10** was reacted with a Grignard reagent to give ketone **11**, which was subsequently transformed to a mixture of oximes **12** (Fig. 3b). A three-step reaction sequence involving oxime reduction, Boc deprotection and nitrosation with ^15^N-sodium nitrite led to amine ^15^N-**4**. Racemic **4** was crystallised from a methanol/diethyl ether mixture and its molecular structure was confirmed by X-ray crystal structure analysis. (Supplementary Table 2 and Supplementary Data 2).

### Dihydrosydnone *N*-oxide 1 is an intermediate at the junction between the biosynthesis of valdiazen and fragin

**Fig. 4:**
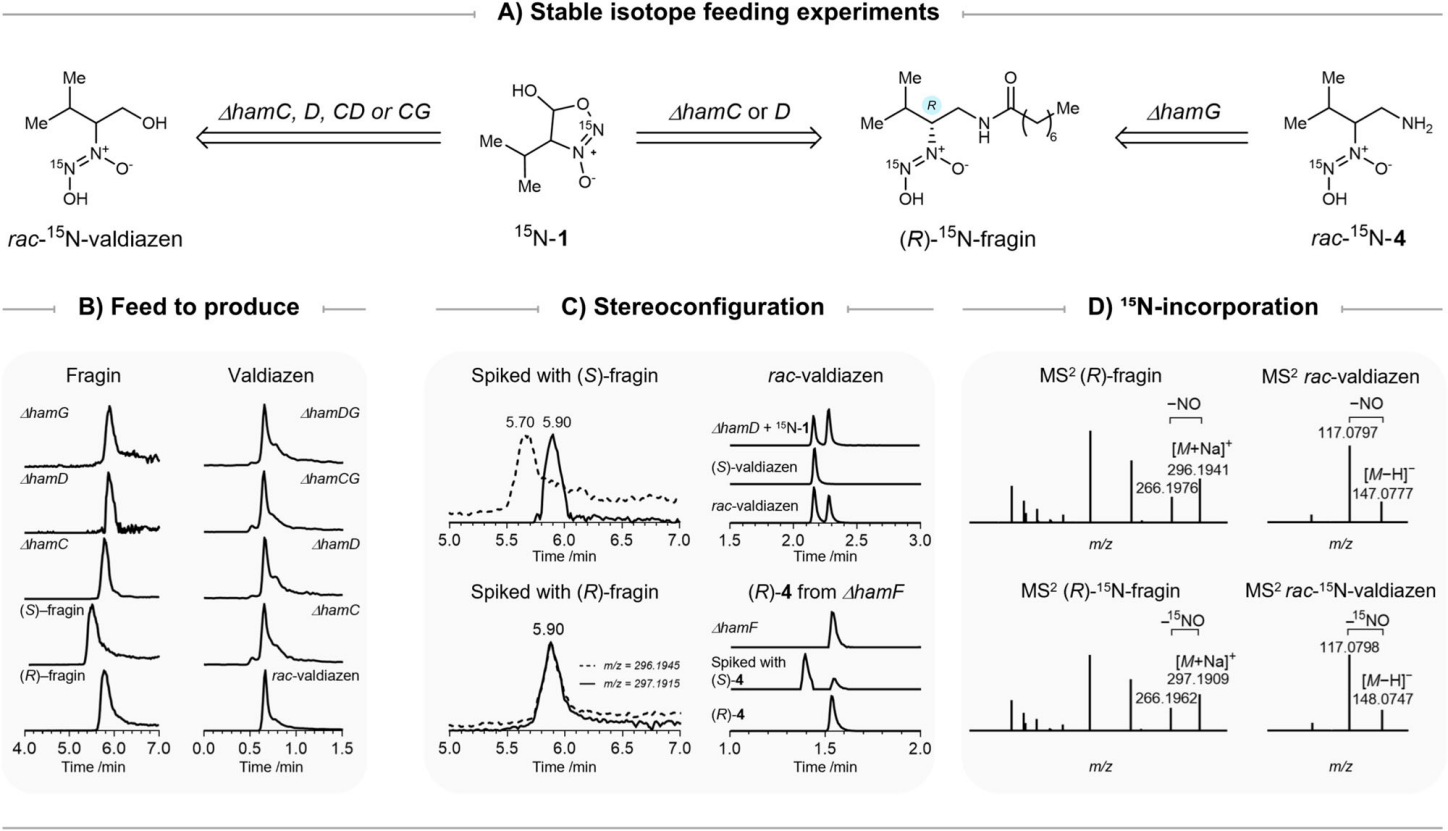
Results of feeding experiments. **A** Dihydrosydnone *N*-oxide **1** and β-amino diazeniumdiolate **4** are competent biosynthetic intermediates for the production of fragin and valdiazen. **B Feeding experiments**: Extracted-ion chromatogram (EIC) traces showed ^15^N-valdiazen production and the stereoselective (*R*)-^15^N-fragin production by feeding ^15^N-labelled intermediates in mutants related to *hamC, hamD*, and *hamG*. **C Configuration**: The configuration of fragin produced in feeding experiments was confirmed by spiking with unlabelled (*R*)- or (*S*)-fragin. One example set of EIC traces from *ΔhamD* feeding experiments is shown here. FDAA-derivatisation and spiking experiments confirmed the configuration of valdiazen and amine **4**. **D** ^15^**N-incorporation**: The incorporation of ^15^N-labels was confirmed by HRMS/HRMS spectra.

With both isotopically labelled compounds in hand, feeding experiments were performed in *B. cenocepacia* H111 mutants *ΔhamC, ΔhamD*, and *ΔhamG*, to investigate whether ^15^N-**1** and ^15^N-**4** can be incorporated into the biosynthetic pathway. To detect both fragin enantiomers, a separation method was developed on an ultra-high-performance liquid chromatography (UHPLC) instrument connected to a high-resolution mass spectrometer (HRMS). A screening of columns was performed, and the chiral Lux^®^ i-Amylose-3 (*Phenomenex*) was selected for its best separation performance. With the developed method (**see Methods**), both fragin enantiomers were differentiated by directly injecting cell-free supernatants. All feeding experiments were performed in biological triplicates (Supplementary Table 3). We observed stereoselective (*R*)-^15^N-fragin production when supplementing racemic dihydrosydnone *N*-oxide ^15^N-**1** in both *ΔhamC mutant* and *ΔhamD* mutant. In contrast for the *ΔhamG* mutant, compound **4** was instead required to produce (*R*)-^15^N-fragin (Fig. 4a, b). As expected, in the double mutants *ΔhamCG* and *ΔhamDG*, supplementation of dihydrosydnone *N*-oxide ^15^N-**1** did not result in fragin production (Supplementary Fig 1). To fully ascertain the stereogenic outcomes of ^15^N-fragin production, cell-free supernatants of all mutant cultures were spiked with enantiomerically pure (*R*)- and (*S*)-fragin, respectively (Fig. 4c and Supplementary Fig 2). A clear distinction between analytical fragin and ^15^N-labelled fragin could be observed by high-resolution mass spectrometry (HRMS), in addition to a characteristic loss of ^15^NO for ^15^N-labelled fragin detected by HRMS/HRMS (Fig. 4d).

Similar feeding experiments were conducted to deconvolute the valdiazen biosynthesis. Due to the detection challenges of valdiazen, each supernatant sample was extracted before the analysis by UHPLC-HRMS in negative ion mode using an NH_4_HCO_3_ buffer to increase sensitivity. As expected, supplying ^15^N-**4** to the *ΔhamG* mutant did not result in valdiazen production (Supplementary Fig 3). Interestingly, valdiazen was produced in all mutants tested when fed with dihydrosydnone *N*-oxide ^15^N-**1** (Fig. 4b). The characteristic loss of ^15^NO from valdiazen can also be seen in HRMS/HRMS in negative ion mode (Fig. 4d). We then extended the feeding experiments to the remaining genes of the *ham* cluster (Supplementary Fig 4 and Supplementary Table 3). When providing dihydrosydnone *N*-oxide ^15^N-**1**, the *ΔhamA, ΔhamB*, and *ΔhamE* mutants were all capable of synthesising (*R*)-^15^N-fragin and ^15^N-valdiazen. Notably, after spiking with analytical (*R*)-fragin, the amount of endogenous unlabelled fragin in *ΔhamB* was so high that the signal of its naturally occurring ^13^C-isomer ([*M*+Na]^+^ = 297.1978) dominated the signal of the ^15^N-labelled fragin ([*M*+Na]^+^ = 297.1915). Nevertheless, spiking with analytical (*S*)-fragin indirectly confirmed the (*R*)-configuration of the labelled fragin produced by *ΔhamB* (Supplementary Fig 2). Although HamB was previously found to be essential for the antifungal activity of *B*. *cenocepacia* H111^11^, we detected the presence of natural fragin in the *ΔhamB* mutant by UHPLC-HRMS. We hypothesised that the production of fragin was much lower in *ΔhamB* compared to the wild type, which led to the low antifungal activity in a disc-diffusion assay^11^. The presence of valdiazen in all strains was unexpected since it was postulated that the reductase domain of NRPS HamD was responsible for the two-electron reduction at the last biosynthetic step^21,41–43^. These observations suggest that the reduction of aldehyde/hemiacetal to alcohol might be mediated by an enzyme outside the *ham* cluster.

### Enantioseparation of valdiazen and β-amino diazeniumdiolate 4

The separation of valdiazen on chiral stationary phases with conventional UHPLC methods was challenging, because of its highly polar, volatile, and sensitive nature. Similar challenges were encountered with amine analogue **4**, as a valid biosynthetic intermediate for fragin production. To examine the configuration of valdiazen and compound **4** in supernatants, Marfey’s reagent (FDAA) was chosen to derivatise the diazeniumdiolates before UHPLC. FDAA is a well-known reagent that reacts with primary amines via nucleophilic aromatic substitution allowing enantioseparation of optical isomers such as amino acids^17,44^. We speculate that diazeniumdiolate is nucleophilic towards alkyl and aryl halides under mild basic conditions^26,45^. Pleasingly, pre-column derivatisation with FDAA enabled chiral separation of valdiazen and amine **4**. Consistent with the results from our previous study^11^, ^15^N-valdiazen derived from the feeding experiments was indeed produced as a racemate (Fig. 4c). FDAA was also capable of capturing unlabelled **4** in the supernatants from *ΔhamF* cultures without exogenous isotopically labelled tracers. By spiking with FDAA-derivatised enantiomerically pure (*R*) or (*S*)-**4**, we demonstrated that only (*R*)-**4** was present in the supernatant (Fig. 4c).

Bacterial cells may preferentially uptake and export one enantiomer over the other^46,47^. To investigate the configuration of amine **4** produced inside cells, we repeated our analysis with both supernatants and cell lysates from the *ΔhamF* culture. Both EIC traces and HRMS/HRMS spectra clearly showed stereoselective production of (*R*)-**4** in both *ΔhamF* supernatants and lysates (Supplementary Fig 5). Overall, this marks the first time that natural (*R*)-β-amino diazeniumdiolate **4** has been detected from cell-free supernatants and cell lysates, providing convincing evidence that (*R*)-**4** is produced stereoselectively and acts as a genuine biosynthetic intermediate in the biosynthesis of (*R*)-fragin.

### Homochirality in valdiazen signalling

**Fig. 5:**
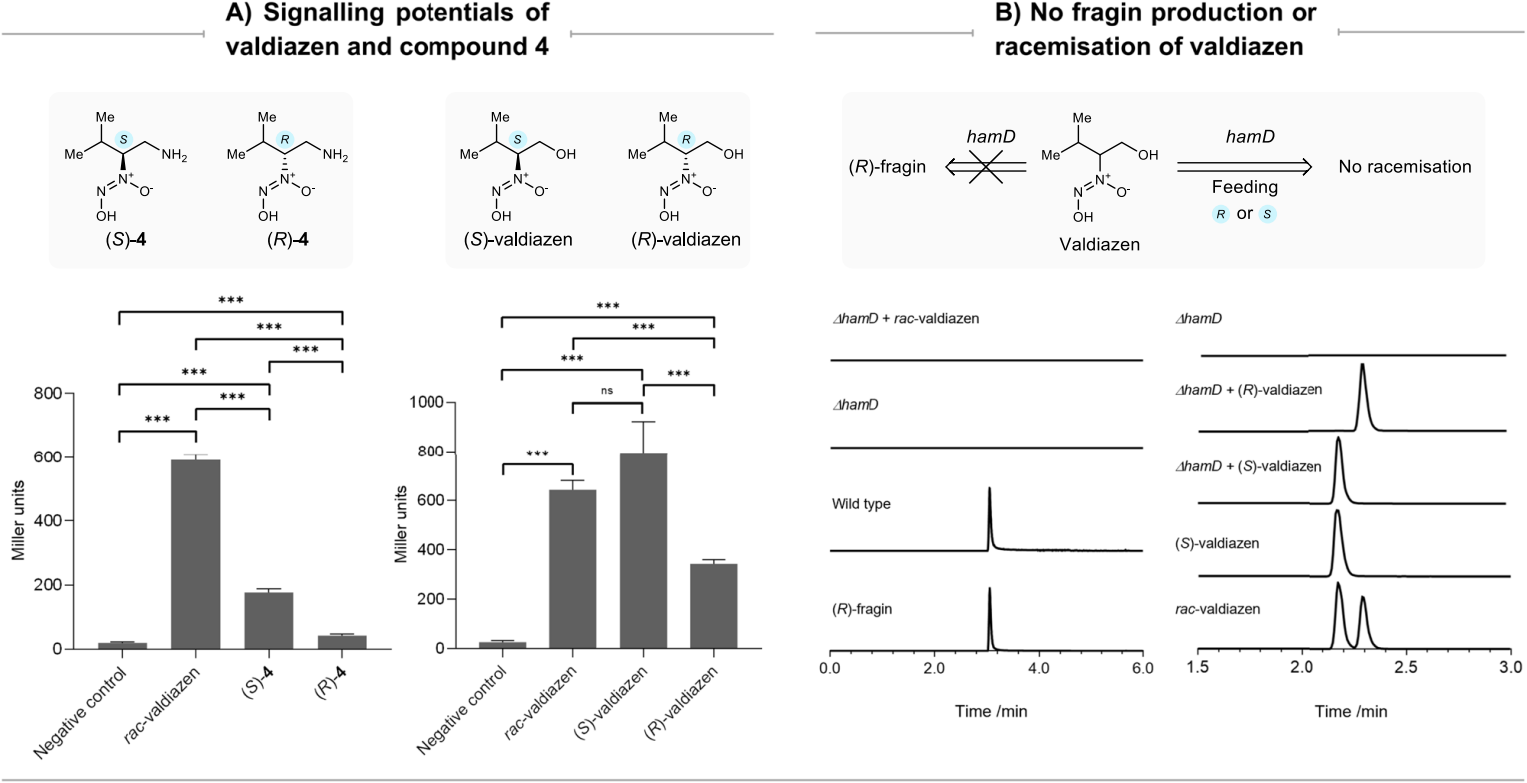
Valdiazen and amino derivative 4 as potential signalling molecules and control experiments. **A** Valdiazen and β-amino diazeniumdiolate **4** showed distinct signalling potentials at 50 μM in inducing the *hamABCDE* operon promoter in a β-galactosidase assay. Statistical analysis was performed with one way analysis of variance (ANOVA) followed by Tukey’s multiple comparison tests. In each plot, a total of 6 comparisons were made between each pair of results. **B** Extracted-ion chromatogram (EIC) traces confirmed that valdiazen is not a direct biosynthetic precursor in a *ΔhamD* mutant: The left set of EIC traces confirmed that no fragin was produced when feeding *rac*-valdiazen to a *ΔhamD* mutant. The right set of EIC traces showed that valdiazen enantiomers fed to a *ΔhamD* mutant did not racemise. Additional abbreviations: ns = not significant; ***represents p ≤ 0.001, n = 3.

We tested the efficacies of valdiazen and β-amino diazeniumdiolate **4** to serve as a signalling molecule by measuring the β-galactosidase activities of a transcriptional fusion of the *hamA* promoter to *lacZ* in the *ΔhamD* mutant (Fig. 5a). Both metabolites induced the *hamA* promoter, with valdiazen showing generally higher activity compared to amine **4**. The (*S*)-enantiomers of both metabolites showed significantly higher β-galactosidase activities than the (*R*)-enantiomers. In the case of amine **4**, the observed discrepancy in activity could be explained by the fact that HamF directs (*R*)-**4** to the biosynthesis of (*R*)-fragin in the *ΔhamD* mutant. Feeding valdiazen to the *ΔhamD* mutant did not restore fragin production, indicating that valdiazen does not serve as a direct precursor in the biosynthesis of fragin (Fig. 5b). Analysis of the FDAA-derivatised cell-free supernatants showed no racemisation of the supplied valdiazen (Fig. 5b). However, at present we cannot rule out the possibility that the transporter required for the uptake of valdiazen prefers the (*S*)- over the (*R*)-enantiomer. In any case, these results collectively show that (*S*)-valdiazen is more efficient at inducing transcription of the *hamA* promoter than (*R*)-valdiazen.

## Conclusion

In summary, our study provided evidence for stereodivergent biosynthesis in the *ham* cluster of *B. cenocepacia* H111, which leverages keto-enol tautomerisation and enzyme-mediated dynamic kinetic resolution to produce *rac*-valdiazen and (*R*)-fragin. By using synthetic chemistry and gene disruption, we deconvoluted the late steps of (*R*)-fragin and *rac*-valdiazen biosynthesis and suggested a mechanism of stereodiversification via the intermediate dihydrosydnone *N-*oxide **1**. The dihydrosydnone *N-*oxide **1** and β-amino diazeniumdiolate **4** were identified as biosynthetically relevant intermediates in the production of (*R*)-fragin and *rac*-valdiazen. Our results also suggest that the racemisation during the biosynthesis is not mediated by racemases or epimerases, but rather via the intrinsic reactivity of keto-enol tautomerisation. The racemic intermediate can then be processed via enzyme-mediated dynamic kinetic resolution, where only the (*R*)-enantiomer is recognised by HamG and reductively aminated to give (*R*)-**4**. Finally, acylation of (*R*)-**4** by HamF results in (*R*)-fragin. HamF is also capable of mediating enzymatic kinetic resolution when providing racemic **4**. To support our updated biosynthetic pathway proposed here, a summary of metabolite analysis performed by our group is tabulated (Supplementary Table 4).

Research of the past three decades provided overwhelming evidence that most bacteria are capable of communicating with each other with the aid of small signalling molecules. In most cases, these signalling systems are used to monitor the density of the population to control social behaviours, a phenomenon commonly referred as quorum sensing (QS)^48,49^. *N*-acyl homoserine lactones (AHL) are QS signalling compounds that are widespread in Gram-negative bacteria^50^. Both l- and d-AHLs have been identified, with d-AHLs being generally much less efficient in inducing the QS responses^51–53^. Similar to classic AHL-based QS systems, we discovered that both stereoisomers of valdiazen were biosynthesised, although (*S*)-valdiazen displayed significantly higher activity than its enantiomeric counterpart. We were intrigued that the signalling molecule was not produced in a stereospecific manner, given the superior biological activity of one of the enantiomers. We hypothesised that the less potent stereoisomer of the autoinducer could in fact act as a partial agonist.

This study utilised both chemical synthesis and biosynthesis experiments in wild-type and mutant bacteria. By synthetic chemistry, tool molecules were developed to tackle intricate biosynthetic questions. In the meantime, the pursuit of biological questions led to the discovery of a new chemical scaffold, dihydrosydnone *N*-oxide. This unique heterocycle may serve as a new NO-releasing motif. Moreover, NRPS clusters with a C-terminal reductase have also been identified in other bacteria, such as *Mycobacterium tuberculosis* and *Bacillus brevis*^42,54–56^. Our study may therefore serve as a blueprint for the identification of intermediates that represent branching points for the biosynthesis of bioactive compounds with different configurations.

## Methods

### Chemical synthesis and characterisation

All information and data regarding chemical synthesis and characterisation can be found in Supplementary Methods in the Supplementary Information.

### Bacterial strains and plasmids

Strains and plasmids used in this study are listed in Supplementary Table 5.

### Construction of *Burkholderia cenocepacia* H111 deletion mutants

Construction of H111 strains with single gene deletions in *hamC* and *hamD* has been described in our previous study^11^.

Single deletion mutant H111 *ΔhamG* and double deletion mutants H111 *ΔhamCG* and H111 *ΔhamDG* were constructed using the pGPI-SceI/pDAI system^57^. Briefly, the upstream and downstream homology regions flanking the region to be deleted (*hamC* and *hamD* genes) were cloned into pGPI-SceI::TetAR and the plasmid was transferred to the *hamG* mutant of H111. Correct integration of the plasmid into the host genome of *hamG* mutant was verified by PCR. The plasmid pDAI::Gm^R^, which carries the I-SceI nuclease, was transferred into the target strain, which resulted in a double-strand DNA break at its recognition site, linearising the chromosome and resulting in a second homologous recombination event. Conjugants were selected on PIA Gm plates and screened μM by PCR using the check primers. Colonies were patched on PIA and PIA Gm20 to isolate colonies that are cured of the pDAI.

### Stable isotope feeding experiments

The composition of ABG minimal medium has been described in our previous study^11^.

Different mutants of H111 were grown in ABG minimal medium containing 0.005 % yeast extract with agitation (220 rpm) at 37 °C for 24 h, after which 50 μM ^15^N-intermediate was added to the cultures and subsequently incubated for 48 h. Bacterial cultures were centrifuged at 5000 rpm with an Eppendorf Centrifuge 5804R and supernatants were filtered with a Millipore Steritop Vacuum Bottle Top Filter 0.22 μm system to remove all the bacterial cells. The ^15^N-intermediates used in this study include ^15^N-dihydrosydnone *N-*oxide **1**, and ^15^N-β-amino diazeniumdiolate analogue **4**.

### Assessment of promoter activity in liquid culture

Construction of the transcriptional lacZ fusions with the putative promoters of the *hamABCDE* operon has been described previously^11^.

Promoter activity of transcriptional lacZ fusions by β-galactosidase assays was performed as previously described with minor modifications^58^. Briefly, bacterial cells were grown overnight in ABG minimal medium and synthetic valdiazen or β-amino diazeniumdiolate **4** dissolved in methanol was added to the cultures, where indicated, to have a final concentration of 50 μM. Bacterial cells were harvested, resuspended in 2 mL Z-buffer and 1 mL bacterial suspension was used to measure the optical density (OD_600_). Cells were lysed by adding chloroform (25 μL) and sodium dodecyl sulphate (SDS; 25 μL, 0.01%) to the residual 1 mL of bacterial suspension and briefly vortexed. The resulting mixture was incubated for 10 min at 28 °C. Subsequently, the reaction was initiated by adding *o*-nitrophenyl-β-galactosidase (ONPG; 200 μL, 4 mg/mL), vortexed briefly and incubated at room temperature. The reaction was stopped by the addition of 500  μL 1 M aqueous Na_2_CO_3_. Cell debris was removed by centrifugation (10 min, 15000 rpm) and the absorbance at 420 nm (OD_420_) was measured. The β-galactosidase activity was calculated using the following equation: Activity [Miller units] = (1000 × OD_420_) / (time [min] × volume [mL] × OD_420_), where time is the incubation time measured in minutes and volume is the number of cells used in mL.

### Bacterial lysates production

H111 *ΔhamF* strain was grown in ABG medium (150 mL) for 48 h and the cells were pelleted by centrifugation at 6000 rpm for 10 min. The supernatant was filter sterilised, while the cell pellets were washed once with ABG medium (30 mL), and resuspended in ABG medium (30 mL). Cell lysate was obtained by lysis of the cell suspension using a Continuous Flow Cell Disruptor (CF1 model, *Constant Systems*). Three biological replicates were employed in the study.

### Chiral HPLC separation and detection of fragin

The supernatants were transferred to Eppendorf tubes, centrifuged (5 min, 20238 rcf) and part of the liquid was transferred to UHPLC vials. The analysis was performed on a Vanquish Horizon UHPLC system. The separation was achieved using a Lux^®^ i-Amylose-3 column (*Phenomenex*, 50 × 2.0 mm, 3 μm) with a flow of 0.3 mL/min, a temperature of 45 °C, an injection volume of 3 μL, and a solvent system composed of A (H_2_O + 0.1% HCO_2_H) and B (MeOH + 0.1% HCO_2_H). The column was equilibrated for 0.5 min at 40% B, the gradient of the run started with isocratic conditions with 40% B for 0.5 min, increasing to 90% B over 6.5 min, followed by an increase to 100% B within 1 min. 100% B was held for 1 min, then the gradient was decreased to 40% B within 0.4 min, and the final conditions were held for 0.6 min. For the results obtained from *ΔhamC* mutants, the supernatant was concentrated, and dissolved in MeOH before analysis.

The ion source parameters of the HRMS (Exploris 240, *ThermoFischer*) in positive ion mode were set as follows: spray voltage 3.5 kV; capillary temperature 325 °C; sheath gas 50 L min^–1^; aux gas 10 L min^–1^; sweep gas 1 L min^−1^; and vaporizer temperature 340 °C. Full scan analysis was set up at a resolution of 60000 with a scan range of 100–1500 *m*/*z* in positive ion mode and a ddMS_2_ was set up with a resolution of 30000, a scan width of 1 Da, a HCD collision energy at 10, 30, and 50% and was triggered for the targeted masses at 296.1945 *m*/*z* for [fragin + Na]^+^ and 297.1915 *m*/*z* for [^15^N-fragin + Na]^+^. The EASY-IC^TM^ internal calibration system was used at the beginning of each run. (*R*) or (*S*)-fragin were identified by comparison of their retention times with an analytical standard and by analysis of their MS/MS spectra. To further confirm the configuration of fragin, a spiking experiment was performed by adding a solution of the (*R*) and (*S*)-fragin to each supernatant, respectively, before UHPLC separation. The separation and detection procedures were performed for *ΔhamA, ΔhamB, ΔhamC, ΔhamD, ΔhamE, ΔhamCG*, and *ΔhamDG* fed with ^15^N-**1** and with *ΔhamG* fed with ^15^N-**4**. Experiments involving supernatants were repeated in triplicate using the biological triplicates generated from the respective feeding experiments. All samples were centrifuged (5 min, 20238 rcf) and transferred to HPLC vials before analysis.

### Achiral HPLC separation and detection of fragin

To improve the sensitivity of the detection, a more sensitive method to detect fragin was developed using an achiral column. Briefly, the supernatants were transferred to Eppendorf tubes, centrifuged (5 min, 20238 rcf) and part of the liquid was transferred to UHPLC vials. The analysis was performed on a Vanquish Horizon UHPLC system. The separation was achieved using an EVO C18 column (*Phenomenex*, 50 × 2.1 mm, 1.7 μm) with a flow of 0.4 mL/min, a temperature of 40 °C, an injection volume of 1 µL, and a solvent system composed of A (H_2_O + 0.1% HCO_2_H) and B (MeCN + 0.1% HCO_2_H). The column was equilibrated for 0.5 min at 5% B, the gradient of the run started with isocratic conditions with 5% B for 0.5 min, increased to 95% B over 3.5 min, and was kept at 95% B for 1 min. The ion source parameters of the HRMS (Exploris 240, *ThermoFischer*) in positive ion mode were set as follows: spray voltage 3.5 kV; capillary temperature 325 °C; sheath gas 50 L min^–1^; aux gas 10 L min^–1^; sweep gas 1 L min^−1^; and vaporizer temperature 340 °C. Full scan analysis was set up at a resolution of 60000 with a scan range of 100–1500 *m*/*z* in positive ion mode and a ddMS_2_ was set up with a resolution of 30000, a scan width of 1 Da, a HCD collision energy at 10, 30, and 45% and was triggered for the targeted masses at 244.2145 *m*/*z* for [fragin – NO + H]^+^ and 274.2125 *m*/*z* for [fragin + H]^+^. The EASY-IC^TM^ internal calibration system was used at the beginning of each run. Fragin was identified by comparison of the retention time of the analytical standard (3.05 min) and by analysis of its MS/MS spectra. The separation and detection procedures were performed for WT, *ΔhamD*, and *ΔhamD* fed with *rac*-valdiazen. Experiments involving supernatants were repeated in triplicate using the biological triplicates generated from the respective experiments.

### Achiral HPLC separation and detection of valdiazen

For valdiazen extraction, cell-free supernatant was adjusted to a pH value of 11 with 10 M aq. NaOH and extracted twice with half of the corresponding volumes of dichloromethane. The dichloromethane phases were discarded, and the water phase was subsequently adjusted to a pH value of 5 with 10 M aq. HCl and extracted twice with half of the corresponding volumes of dichloromethane. The two dichloromethane phases were combined, dried using anhydrous magnesium sulphate (Sigma-Aldrich, Switzerland), filtered, and concentrated *in vacuo*. The concentrated extracts were stored at −20 °C. The extracts were dissolved in MeOH (1 mL), transferred to Eppendorf tubes, centrifuged (5 min, 20238 rcf) and part of the liquid was transferred to UHPLC vials.

The analysis was performed on a Vanquish Horizon UHPLC system. The separation was achieved using a Luna^®^ Omega PS C18 column (*Phenomenex*, 50 × 2.1 mm, 1.6 μm) with an injection volume of 1 μL and a solvent system composed of A (H_2_O + 0.5 mM NH_4_CO_3_) and B (MeCN). Two analytical methods (A and B) were used and, for both methods, the column was equilibrated for 0.5 min at the starting conditions. **Method A**: The run started with a flow of 0.3 mL/min and isocratic conditions were kept at 0% B for 0.49 min, the flow was increased to 0.4 mL/min over 0.01 min, the gradient was increased from 0 to 95% B within 3 min, 95% B was held for 2 min, the gradient was decreased to 0% B within 0.1 min, and the final conditions were held for 1 min. **Method B**: The flow was 0.4 mL/min and the gradient of the run started with an increase from 0 to 95% B over 3.5 min, 95% B was held for 2 min, the gradient was decreased to 0% B within 0.1 min, and the final conditions were held for 1 min. With method A, valdiazen was detected at 0.67 min and with method B, valdiazen was detected at 0.53 min. The ion source parameters of the HRMS (Exploris 240, *ThermoFischer*) in negative ion mode were set as follows: spray voltage 2.5 kV; capillary temperature 325 °C; sheath gas 50 L min^–1^; aux gas 10 L min^–1^; sweep gas 1 L min^−1^; and vaporizer temperature 325 °C. Full scan analysis was set up at a resolution of 60000 with a scan range of 100–1500 *m*/*z* in negative ion mode and a ddMS_2_ was set up with a resolution of 30000, a scan width of 0.4 Da, a HCD collision energy at 10, 30, and 50% and was triggered for the targeted masses at 147.0775 *m*/*z* for [valdiazen – H]^–^ and 148.0745 *m*/*z* for [^15^N-valdiazen − H]^–^. The EASY-IC^TM^ internal calibration system was used at the beginning of each run. Valdiazen was identified by comparison of the retention time of the analytical standard and by analysis of its MS/MS spectra. The separation and detection procedures were performed for *ΔhamA, ΔhamB, ΔhamC, ΔhamD, ΔhamE, ΔhamCG*, and *ΔhamDG* fed with ^15^N-**1**. Some samples were diluted in H_2_O before analysis due to the high concentration of the targeted compounds. Experiments involving supernatants were repeated in triplicate using the biological triplicates generated from the respective feeding experiments. All samples were centrifuged (5 min, 20238 rcf) and transferred to HPLC vials before analysis.

### Pre-column derivatisation with FDAA

An aliquot of supernatant (10 mL) or cell lysate (2 mL) was concentrated under a gentle flow of nitrogen for 16 h at 40 °C. The residue was resuspended in Milli-Q^®^ water (100 μL), treated with 1% FDAA in acetone (200 μL), 1 M aq. sodium bicarbonate solution (40 μL) and heated at 40 °C for 1 h. The mixture was cooled to r.t. and treated with 2 M aq. HCl (20 μL). The sample was then concentrated under a gentle flow of nitrogen for 1 h to remove acetone.

### Chiral HPLC separation and detection of valdiazen via FDAA pre-column derivatisation

FDAA derivatisation was performed using the analytical standards (*S*)-valdiazen and *rac*-valdiazen at the 5 μmol scale, and with the supernatants (10 mL) of the *ΔhamD* fed with ^15^N-**1**. Experiments involving supernatants were repeated using the biological triplicates generated from the respective feeding experiments. For spiking experiments, samples from supernatants were mixed with FDAA-treated synthetic analytical samples. All samples were centrifuged (5 min, 20238 rcf) and transferred to HPLC vials before analysis.

The analysis was performed on a Vanquish Horizon UHPLC system. The separation was achieved using an EVO C18 column (*Phenomenex*, 50 × 2.1 mm, 1.7 μm) with a flow of 0.4 mL/min, a temperature of 40 °C, an injection volume of 1 μL, and a solvent system composed of A (H_2_O + 0.5 mM NH_4_CO_3_) and B (MeCN). The column was equilibrated for 0.5 min at 5% B, the gradient of the run was increased from 5% to 95% B over 4 min, and was kept at 95% B for 2 min.

The ion source parameters of the HRMS (Exploris 240, *ThermoFischer*) in negative ion mode were set as follows: spray voltage 2.5 kV; capillary temperature 325 °C; sheath gas 50 L min^–1^; aux gas 10 L min^–1^; sweep gas 1 L min^−1^; and vaporizer temperature 340 °C. The EASY-IC^TM^ internal calibration system was used at the beginning of each run. A full scan with a resolution of 60000, a scan range of 140–1000 Da, and RF lens of 70% were used in negative ion mode and a ddMS_2_ was set up with a scan width of 0.4 *m*/*z*, at a resolution of 30000, and with an HCD collision energy at 15, 30, and 50%). Valdiazen-FDAA derivatives (*m*/*z* 399.12699 for [valdiazen-FDAA – H]^-^ and *m*/*z* 400.12402 for [^15^N-valdiazen-FDAA – H]^-^) were identified by comparison of the retention time of the analytical standard (2.17 min for *S* and 2.28 min for *R*) and by analysis of their MS/MS spectra. The separation and detection procedures were performed for *ΔhamD* fed with ^15^N-**1**. Experiments involving supernatants were repeated in triplicate.

### Chiral HPLC separation and detection of the amine analogue 4 via pre-column derivatisation with FDAA

FDAA derivatisation was performed using *ΔhamF* supernatant (10 mL) or *ΔhamF* cell lysate (2 mL). The same conditions were used for the synthetic enantiomerically pure amine analogues **4** (*R* and *S*) at the 5 μmol scale as were used for the analytical standards and spiking experiments later. The sample was then suspended with MeCN (200 µL) for the lysate, or with MeCN up to a volume of 1 mL for the supernatant. For spiking experiments, samples from supernatants were mixed with FDAA-treated synthetic analytical samples. All samples were vortexed, centrifuged (5 min, 20238 rcf) and transferred to HPLC vials before analysis. Further dilution with MeCN can be made depending on sample concentration. Experiments involving supernatants were repeated in triplicate using the biological triplicates generated from the respective feeding experiments.

The analysis was performed on a Vanquish Horizon UHPLC system. The separation was achieved using an EVO C18 column (*Phenomenex*, 50 × 2.1 mm, 1.7 μm) with a flow of 0.4 mL/min, a temperature of 30 °C, an injection volume of 1–3 μL, and a solvent system composed of A (H_2_O + 0.1% HCOOH) and B (MeCN + 0.1% HCOOH). The column was equilibrated for 0.5 min at 5% B, the gradient of the run started to increase from 5% to 95% B over 4 min, and was kept at 95% B for 2 min. The ion source parameters of the HRMS (Exploris 240, *ThermoFischer*) in positive ion mode were set as follows: spray voltage 3.5 kV; capillary temperature 325 °C; sheath gas 50 L min^–1^; aux gas 10 L min^–1^; sweep gas 1 L min^−1^; and vaporizer temperature 340 °C. The EASY-IC^TM^ internal calibration system was used at the beginning of each run. A full scan with a resolution of 240000, a scan range of 400.1–400.2 Da, and RF lens of 70% were used in negative ion mode. Amine **4** FDAA derivatives (*m*/*z* 400.15752 for [amine **4**-FDAA + H]^+^) were identified by comparison of the retention time of derivatized analytical standards 1.37 min for *S* (1.43 min using a new column) and 1.54 min for *R* (1.60 min using a new column). For an optimal detection of the peak, the mass tolerance was set to 3 ppm. The separation and detection procedures were performed using the supernatant and cell lysate of *ΔhamF*. Experiments involving supernatants and lysates were repeated in triplicate.

### Data analysis and plot generation

The UHPLC-HRMS data were visualized with FreeStyle (*ThermoFischer*), and the chromatograms and spectra were exported as CSV files. GraphPad Prism (version 10.1.2) was used to plot the chromatographic data points in the relevant range of retention time from a single feeding experiment. The intensity data from the chromatograms were normalised with the highest intensity set to 100% before being plotted as a line graph. Overlaid plots comparing negative control and positive results were plotted using intensity without normalisation. MS/MS graphs were plotted directly from the source data. For β-galactosidase assay results, one-way analysis of variance (ANOVA) followed by Tukey’s multiple comparison tests were performed using the original algorithm package from GraphPad Prism (version 10.1.2).

### Reporting summary

Further information on research design is available in the Nature Portfolio Reporting Summary linked to this article.

## Supplementary information

Supplemental Material

Reporting Summary

Supplementary Data 1

Supplementary Data 2

Supplementary Data 3

## Data Availability

The data that support the findings of this study are available within the main text and Supplementary Information. The crystal structures used in this work can be obtained from the Cambridge Crystallographic Data Centre with the identifier “2351242”, “2351243” and “2351244”. The UHPLC-MS data used during this study can be found on the Zenodo platform (https://zenodo.org/records/14067000).
