# Supplementary material for: Functional biosynthetic stereodivergence in a gene cluster via a dihydrosydnone N-oxide
Source: Commun Chem. 2024 Dec 19;7:301. doi: 10.1038/s42004-024-01372-3 (PMC11659414; doi:10.1038/s42004-024-01372-3)
Supplement: Supplementary file 1 — Supplemental Material [file 42004_2024_1372_MOESM1_ESM.pdf]

Functional Biosynthetic Stereodivergence in a Gene Cluster via a Dihydrosydnone *N*-oxide

Jiajun Ren[1], Anugraha Mathew[2], María Rodríguez-García[2], Tobias Kohler[1], Olivier Blacque,[1] Anthony Linden,[1] Leo Eberl[2*], Simon Sieber[1*], and Karl Gademann[1,3*]

Affiliations:

[1]Department of Chemistry, University of Zurich, 8057 Zurich, Switzerland.

[2]Department of Plant and Microbial Biology, University of Zurich, 8008 Zurich, Switzerland.

Correspondence and requests for materials should be addressed to K.G. (email: karl.gademann@uzh.ch), S.S. (email: simon.sieber@uzh.ch), or to L.E. (email: leberl@botinst.uzh.ch)

Supplementary Methods

General procedure

The syntheses of (*S*)-fragin, (*R*)-fragin, (*S*)-valdiazen, (*R*)-valdiazen were performed as described in a previous publication.[1]

Unless otherwise stated, all chemicals were of reagent grade and purchased from Sigma-Aldrich-Merck, Acros Organics, Honeywell, Fluorochem or TRC. Solvents for reactions were of analytical grade. Evaporation of solvents *in vacuo* was performed with a rotary evaporator equipped with a water bath at 40 °C and indicated pressure. **Thin layer chromatography** (TLC): reaction control with TLC were performed on Merck TLC plates, silica gel 60 F254 on aluminium with the indicated solvent system; the spots were visualized by UV light (254 nm) or $KMnO_4$ stain. **Flash Chromatography**: Silica gel column chromatography was performed using silica gel 60 (230–400 Mesh) purchased from Sigma-Aldrich and the compounds were eluted with the solvent mixture indicated. **Solid-phase extraction** (SPE) **column**: Discovery® DSC-18 SPE tubes for semi-purification were purchased from Sigma-Aldrich-Merck, and used following their guidelines; **Ultra-high-performance liquid chromatography coupled to mass spectrometry** (UHPLC-MS) **for reaction control**: reaction controls with UHPLC-MS were performed on an *Ultimate 3000 LC* instrument (*Thermo Fisher Scientific*) coupled to a triple quadrupole *Quantum Ultra EMR MS* (*Thermo Fisher Scientific*) using a reversed-phase column (*Kinetex*® EVO C18; 1.7 µm; 100 Å, 50 × 2.1 mm; *Phenomenex*), heated to 40 °C. The LC was equipped with an *HPG-3400RS* pump, a *WPS-3000TRS* autosampler, a *TCC-3000RS* column oven and a *Vanquish DAD* detector (all *Thermo Fisher Scientific*). The following solvents were used as eluents: H_2O+0.1 % HCO_2H (A), MeCN+0.1 % HCO_2H (B). The MS was equipped with an H-ESI II ion source. The source temperature was 250 °C, the capillary temperature 270 °C and capillary voltage 3500 V, and datasets were acquired at resolution 0.7 on Q3 in centroid mode. **Infrared spectra** (IR): IR spectra were recorded on *SpectrumTwo FT-IR Spectrometer* (*Perkin–Elmer*) equipped with a *Specac Golden GateTM ATR* (attenuated total reflection) accessory. The samples were applied as neat samples or as films. **Melting points** (m.p.): Melting points were determined using the *Büchi* B-545 apparatus in open capillaries and are uncorrected. **Nuclear magnetic resonance spectra** (NMR): ^{1}H-NMR spectra were recorded using the indicated deuterated solvents at 298 K on the Bruker *AVII* or *III-500* (500 MHz with Cryo-BBO, TXI, BBI or BBO probe) or *AVII-400* (400 MHz with QNP, BBO or BBFO probe); ^{13}C-NMR spectra were recorded using the indicated solvents and on the same instruments. **High resolution electrospray ionization mass spectrometry for compound characterization** (HR-ESI-MS): HRMS for the characterization of synthetic compounds were measured on a *Dionex Ultimate 3000* UHPLC system (*ThermoFischer Scientifics*, Germering, Germany) connected

to a QExactive MS with a heated ESI source (*ThermoFisher Scientific*, Bremen, Germany); onflow injection of 1 µL sample (c = ca. 50 µg mL^{-1} in the indicated solvent) with an *XRS* auto-sampler (*CTC*, Zwingen, Switzerland); flow rate 120 µL min^{-1}; ESI: spray voltage 3.0 kV, capillary temperature 280 °C, sheath gas 30 L min^{-1}, aux gas 8 L min^{-1}, s-lens RF level 55.0, aux gas temperature 250 °C (N_2); full scan MS in the alternating (+)/(−)-ESI mode; mass ranges 80–1200 *m/z*, 133–2000 *m/z*, or 200–3000 *m/z* at 70000 resolution (full width half-maximum); automatic gain control (AGC) target of 3.00×10^6; maximum allowed ion transfer time (IT) 30 ms; mass calibration to <2 ppm accuracy with *Pierce*® ESI calibration solns. (*ThermoFisher Scientific,* Rockford, USA); lock masses: ubiquitous erucamide (*m/z* 338.34174, (+)-ESI) and palmitic acid (*m/z* 255.23295, (–)-ESI). **Ultra-high-performance liquid chromatography coupled to high resolution electrospray ionization mass spectrometry for the detection of fragin and valdiazen** (UHPLC-HR-ESI-MS): The samples were measured on a Vanquish Horizon UHPLC system (*ThermoFischer*) equipped with a quaternary pump, an autosampler, a Diode Array Detector, a Split Sampler HT, a Binary Pump H, and Column Compartment. The UHPLC system used was connected to a HRMS (Exploris 240, *ThermoFischer*) instrument. **Specific optical rotation**: Specific optical rotations were recorded using a *Jasco P-2000 Polarimeter* with a path length of 1 dm using the 589.3 nm D-line of sodium. Measurements were recorded at the indicated temperature (in °C) and concentration (in g/100 mL) in the indicated solvent. **Preparative high performance liquid chromatography (preparative HPLC)**: Purification was made using a prominence modular HPLC instrument (*Shimadzu*) coupled to an *SPD-20A* UV/Vis detector (*Shimadzu*) with a reversed-phase column (*Phenomenex Synergi*™ 10 µm Hydro-RP 80 Å, 250 mm × 21.2 mm). The LC was equipped with a *CBM-20A* system controller, *LC-20A* solvent delivery unit, a *DGU-20A* degassing unit, and an *FRC-10A* fraction collector (all units from *Shimadzu*). The conditions used are detailed in the experimental below.

Single crystal X-ray diffraction

The measurements were made at 160 K on a *Rigaku Oxford Diffraction Synergy/Hypix* diffractometer (**1**, **4**) and on a *Rigaku Oxford Diffraction SuperNova/Atlas* area detector diffractometer (**17**) using Cu Kα radiation (λ = 1.54184 Å) and *Oxford Instruments Cryojet XL* coolers. The selected suitable single crystals were mounted in oil on cryo-loops. Pre-experiment, data collection, data reduction and absorption correction were performed with the program suite *CrysAlisPro* (*Version 1.171.40-53/1.171.42.93a, Rigaku Corporation, Wroclaw, Poland, 2019-2023*). Using *Olex2*,[2] the structures were solved with the *SHELXT*[3] small molecule structure solution program and refined with the *SHELXL* program package[4] by full-matrix least-squares minimization on F^2. *PLATON*[5] was used to validate the results of the X-ray analyses. CCDC 2351242 (**1**), 2351243 (**4**) and 2351244 (**17**) contain the supplementary crystallographic data for this paper. The data can be obtained free of charge from The Cambridge Crystallographic Data Centre via www.ccdc.cam.ac.uk/structures.

Supplementary Table 1. Crystallographic data for the dihydrosydnone *N*-oxide **1**.

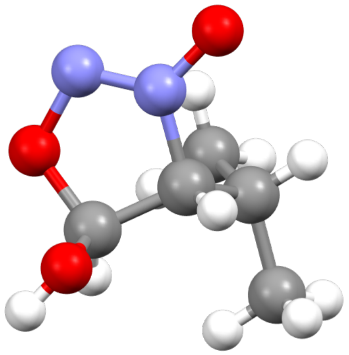

Crystallised from	Diethyl ether and cyclopentane (antisolvent)
Empirical formula	$C_5H_{10}N_2O_3$
Formula weight	146.15
Temperature/K	160.0(1)
Crystal system	orthorhombic
Space group	Pbca
a/Å	11.57870(10)
b/Å	9.31170(10)
c/Å	13.18000(10)
α/°	90
β/°	90
γ/°	90
Volume/Å^3	1421.03(2)
Z	8
ρ_{calc}g/cm^3	1.366
μ/mm^{-1}	0.965
F(000)	624.0
Crystal size/mm^3	0.26 × 0.19 × 0.12
Radiation	Cu Kα (λ = 1.54184)
2Θ range for data collection/°	13.44 to 154.51
Index ranges	$-13 \leq h \leq 14$, $-11 \leq k \leq 10$, $-15 \leq l \leq 16$
Reflections collected	9864
Independent reflections	1507 [R_{int} = 0.0166, R_{sigma} = 0.0106]
Data/restraints/parameters	1507/0/98
Goodness-of-fit on F^2	1.065
Final R indexes [I>=2σ (I)]	R_1 = 0.0310, wR_2 = 0.0804
Final R indexes [all data]	R_1 = 0.0316, wR_2 = 0.0809
Largest diff. peak/hole / e Å^{-3}	0.27/-0.18

Supplementary Table 2. Crystallographic data for amine **4**.

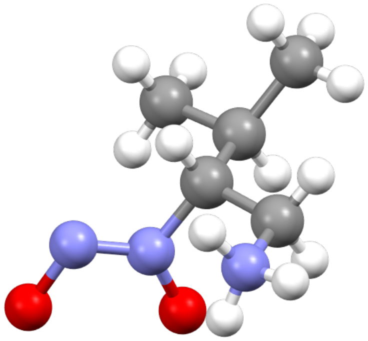

Crystallised from	Methanol and diethyl ether (antisolvent)
Empirical formula	$C_{12}H_{35}ClN_6O_6$
Formula weight	394.91
Temperature/K	160.0(1)
Crystal system	monoclinic
Space group	$P2_1/c$
a/Å	9.9830(2)
b/Å	21.2959(6)
c/Å	11.0843(3)
α/°	90
β/°	106.801(3)
γ/°	90
Volume/Å^3	2255.90(10)
Z	4
ρ_{calc}g/cm^3	1.163
μ/mm^{-1}	1.809
F(000)	856.0
Crystal size/mm^3	0.14 × 0.07 × 0.04
Radiation	Cu Kα (λ = 1.54184)
2Θ range for data collection/°	8.30 to 154.72
Index ranges	$-12 \leq h \leq 12$, $-26 \leq k \leq 25$, $-14 \leq l \leq 14$
Reflections collected	25904
Independent reflections	4767 [R_{int} = 0.0434, R_{sigma} = 0.0320]
Data/restraints/parameters	4767/0/268
Goodness-of-fit on F^2	1.058
Final R indexes [I>=2σ (I)]	R_1 = 0.0471, wR_2 = 0.1349
Final R indexes [all data]	R_1 = 0.0606, wR_2 = 0.1455
Largest diff. peak/hole / e Å^{-3}	0.57/-0.31

Supplementary Table 3. Summary of feeding experiments using ^{15}N-labelled intermediates in various mutants in this study.

	**Production of ^{15}N-labelled compound**		**Average retention time±standard error (min)**	
	rac-valdiazen	(*R*)-fragin	*rac*-valdiazen	(*R*)-fragin
ΔhamA + ^{15}N-**1**	Yes	Yes	0.509±0.0284	6.02±0.0153
ΔhamB + ^{15}N-**1**	Yes	Yes	0.497±0.0300	6.02±0.0115
ΔhamC + ^{15}N-**1**	Yes	Yes	0.659±0.00808	5.76±0.0404
ΔhamD + ^{15}N-**1**	Yes	Yes	0.654±0.00173	5.88±0.0404
ΔhamE + ^{15}N-**1**	Yes	Yes	0.483±0.00808	6.02±0.02
ΔhamG + ^{15}N-**4**	No	Yes	-	5.88±0.0265
ΔhamCG + ^{15}N-**1**	Yes	No	0.654±0.00100	-
ΔhamDG + ^{15}N-**1**	Yes	No	0.654±0.00153	-

Note: Each run in the MS has different amount of total data points and measurements that were scattered over different time points among repeats, it was impossible to average data points without curve fitting or approximation, either of which will compromise the high-quality data obtained from the state-of-art HRMS. To assess the reproducibility among the triplicates, calculation of the mean and standard error (SE) of the time point where maximum intensity is reached was carried out.

Supplementary Table 4. Summary of metabolite analysis from *Burkholderia cenocepacia* H111 WT and mutants.

Feeding compound	Detected compound	Isolation/Detection	HPLC(-MS) Instrument	Reference
**H111 (WT)**				
-	(*R*)-fragin	Extract, isolation, optical rotation and X-ray		Jenul *et al.*[1]
-	*rac*-valdiazen	Extract, isolation, and HPLC normal phase with chiral column	Agilent 1100, Chiralpak OD-H column	Jenul *et al.*[1]
-	(*R*)-fragin	Supernatant, UHPLC reverse phase, chiral column	Vanquish Horizon, Lux® i-Amylose-3 column, MS Exploris 240	This study
[U-$^{13}C_5$]-L-valine	[$^{13}C_5$]-fragin	Extraction, HRMS analysis	Q Exactive MS	Sieber *et al.*[6]
ΔhamA				
^{15}N-**1**	^{15}N-valdiazen	Extraction, UHPLC reverse phase, chiral column	Vanquish Horizon, Luna Omega PS C18 column, MS Exploris 240	This study
^{15}N-**1**	^{15}N-(*R*)-fragin	Supernatant, UHPLC reverse phase, chiral column	Vanquish Horizon, Lux® i-Amylose-3 column, MS Exploris 240	This study
ΔhamB				
^{15}N-**1**	^{15}N-valdiazen	Extraction, UHPLC reverse phase, chiral column	Vanquish Horizon, Luna Omega PS C18 column, MS Exploris 240	This study
^{15}N-**1**	^{15}N-(*R*)-fragin	Supernatant, UHPLC reverse phase, chiral column	Vanquish Horizon, Lux® i-Amylose-3 column, MS Exploris 240	This study
ΔhamC				
^{15}N-**1**	^{15}N-valdiazen	Extraction, UHPLC reverse phase, chiral column	Vanquish Horizon, Luna Omega PS C18 column, MS Exploris 240	This study
^{15}N-**1**	^{15}N-(*R*)-fragin	Supernatant, UHPLC reverse phase, chiral column	Vanquish Horizon, Lux® i-Amylose-3 column, MS Exploris 240	This study
ΔhamD				
-	no fragin no valdiazen detected	Extract, HPLC reverse phase	Dionex HPLC, Gemini-NX column, MS MSQ-ESI	Jenul *et al.*[1]
-	no fragin no valdiazen detected	Supernatant, UHPLC reverse phase	Vanquish Horizon, EVO C18 column, MS Exploris 240	This study
^{15}N-**1**	^{15}N-valdiazen	Extraction, UHPLC reverse phase, chiral column	Vanquish Horizon, Luna Omega PS C18 column, MS Exploris 240	This study
^{15}N-**1**	^{15}N-*rac*-valdiazen	Supernatant, FDAA derivatisation and UHPLC-HRMS	Vanquish Horizon, EVO C18 column, MS Exploris 240	This study

^{15}N-**1**	^{15}N-(*R*)-fragin	Supernatant, UHPLC reverse phase, chiral column	Vanquish Horizon, Lux® i-Amylose-3 column, MS Exploris 240	This study
ΔhamE				
^{15}N-**1**	^{15}N-valdiazen	Extraction, UHPLC reverse phase, chiral column	Vanquish Horizon, Luna Omega PS C18 column, MS Exploris 240	This study
^{15}N-**1**	^{15}N-(*R*)-fragin	Supernatant, UHPLC reverse phase, chiral column	Vanquish Horizon, Lux® i-Amylose-3 column, MS Exploris 240	This study
ΔhamF				
	valdiazen	Conc. supernatant, UHPLC reverse phase	Ultimate 3000, EVO C18 column, TSQ Quantum Ultra	Jenul *et al.*[1]
	(*R*)-**4**	Conc. supernatant, FDAA derivatization and UHPLC-HRMS	Ultimate 3000, EVO C18 column, TSQ Quantum Ultra	This study
	(*R*)-**4**	Cell lysate, FDAA derivatization and UHPLC-HRMS	Ultimate 3000, EVO C18 column, TSQ Quantum Ultra	This study
ΔhamG				
^{15}N-**4**	^{15}N-(*R*)-fragin	Supernatant, UHPLC reverse phase, chiral column	Vanquish Horizon, Lux® i-Amylose-3 column, MS Exploris 240	This study
ΔhamCG				
^{15}N-**1**	^{15}N-valdiazen	Extraction, UHPLC reverse phase, chiral column	Vanquish Horizon, Luna Omega PS C18 column, MS Exploris 240	This study
ΔhamCG				
^{15}N-**1**	^{15}N-valdiazen	Extraction, UHPLC reverse phase, chiral column	Vanquish Horizon, Luna Omega PS C18 column, MS Exploris 240	This study

Supplementary Table 5. Bacterial strains and plasmids used in this study.

Strain or plasmid	Characteristics	Source/Reference
Burkholderia cenocepacia		
H111	Cystic Fibrosis isolate, (Germany)	Gotschlith *et al.*[7]
H111 *ΔhamC*	Unmarked *hamC* deletion mutant	Jenul *et al.*[1]
H111 *ΔhamD*	Unmarked *hamD* deletion mutant	Jenul *et al.*[1]
H111 *ΔhamG*	Unmarked *hamG* deletion mutant	This study
H111 *ΔhamCG*	Double deletion mutant of *hamC* and *hamG*	This study
H111 *ΔhamDG*	Double deletion mutant of *hamD* and *hamG*	This study
Escherichia coli		
Top 10	F-mcrAΔ(mrr-hsdRMS-mcrBC) φ80lacZΔM15 ΔlacX74 nupG recA1 araD139 Δ(ara-leu)7697 galE15 galK16 rpsL(StrR) endA1 λ-	Invitrogen
CC118	λpir Δ(ara,leu)7697araD139 ΔlacX74 galEgalKphoA20 thi-1rpsErpoB(RFR) argE(am) recA1λpir+	Herrero *et al.*[8]
**Plasmids**		
pSU11	Promoter probe vector for lacZ fusion, GmR	O'Grady *et al.*[9]
pGPI-SceI::TetAR	Suicide plasmid with oriR6K, mob+, I-SceI restriction site; TpR TcR	Habjanič *et al.*[10]
pDAIGm-SceI	pDA17 plasmid carrying the I-SceI nuclease gene; GmR	Flannagan *et al.*[11]
phamA-lacZ	pSU11 containing the *hamA* promoter	Jenul *et al.*[1]

Supplementary Table 6. Crystallographic data for the diol **17**.

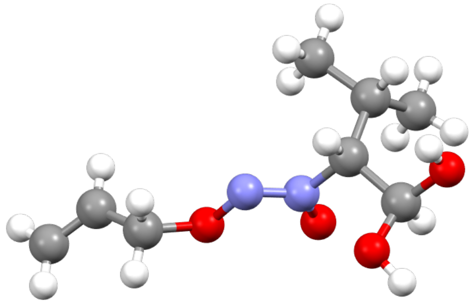

Crystallised from	Pentane and diethyl ether
Empirical formula	$C_8H_{16}N_2O_4$
Formula weight	204.23
Temperature/K	160(1)
Crystal system	monoclinic
Space group	$P2_1/c$
a/Å	9.7461(2)
b/Å	10.1007(4)
c/Å	10.8278(2)
α/°	90
β/°	90.358(2)
γ/°	90
Volume/Å3	1065.89(5)
Z	4
ρcalcg/cm3	1.273
μ/mm-1	0.860
F(000)	440.0
Crystal size/mm3	0.25 × 0.21 × 0.02
Radiation	Cu Kα (λ = 1.54184)
2Θ range for data collection/°	9.07 to 146.00
Index ranges	-12 ≤ h ≤ 12, -12 ≤ k ≤ 11, -13 ≤ l ≤ 12
Reflections collected	18546
Independent reflections	2108 [R_{int} = 0.0239, R_{sigma} = 0.0101]
Data/restraints/parameters	2108/0/138
Goodness-of-fit on F2	1.043
Final R indexes [I>=2σ (I)]	R1 = 0.0316, wR2 = 0.0821
Final R indexes [all data]	R1 = 0.0335, wR2 = 0.0843
Largest diff. peak/hole / e Å-3	0.20/-0.19

Supplementary Fig 1. Extracted-ion chromatogram (EIC) traces demonstrating no fragin was produced when feeding *ΔhamCG* and *ΔhamDG* with ^{15}N-**1**, stacked above the positive result from *ΔhamC* + ^{15}N-**1**.

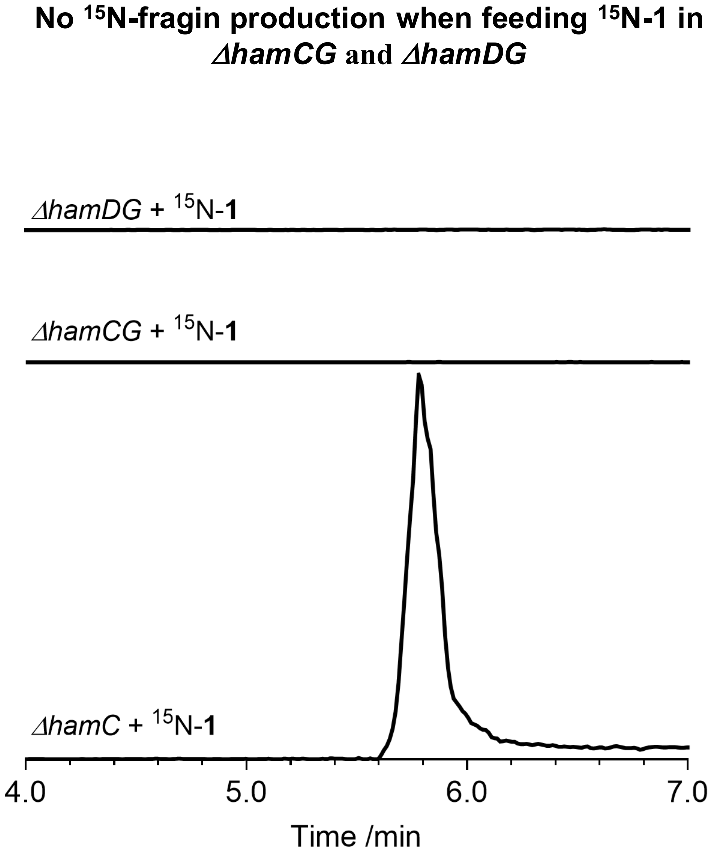

Supplementary Fig 2. EIC traces of different supernatant samples spiked with (*R*) or (*S*)-fragin.

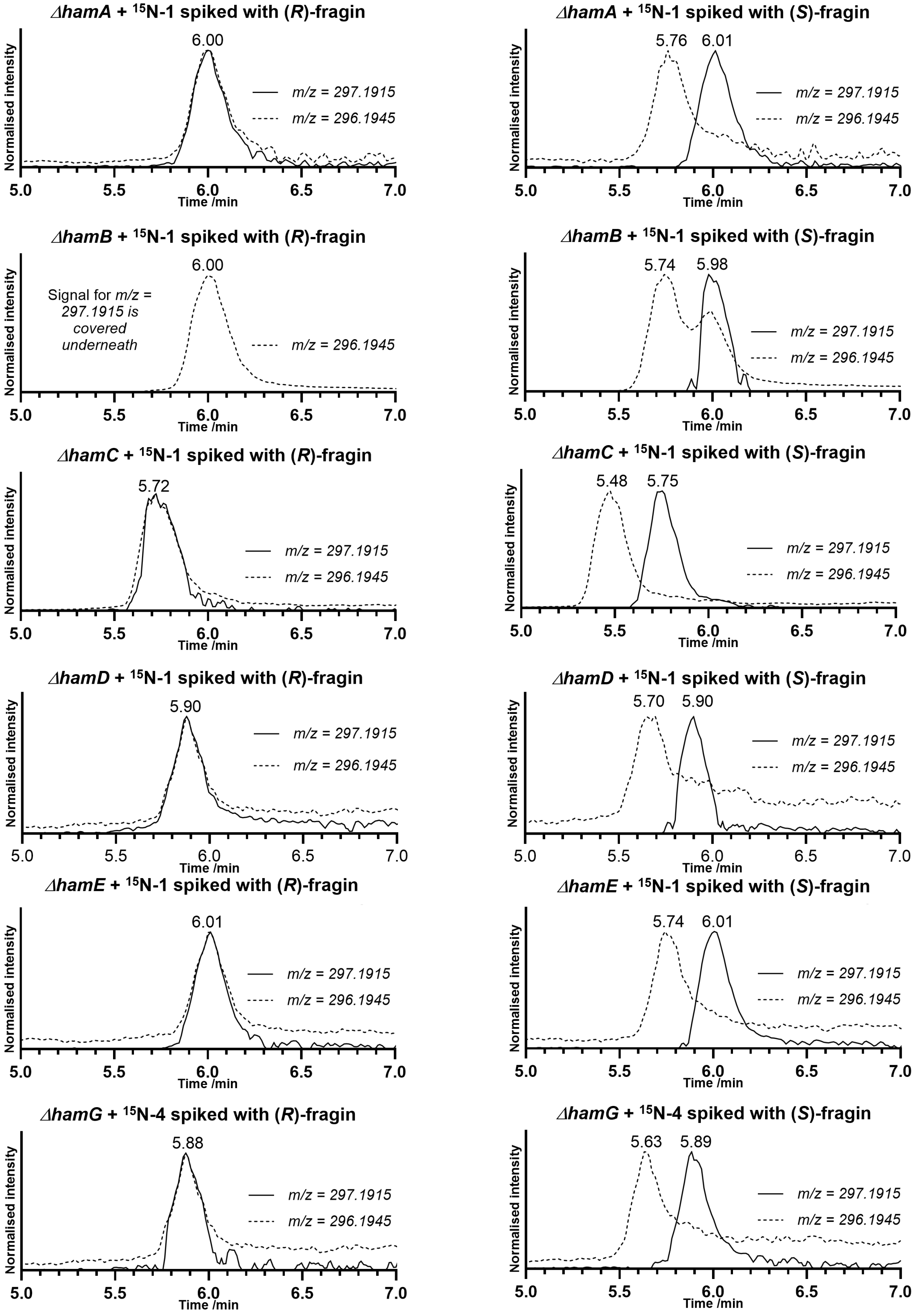

Supplementary Fig 3. EIC traces demonstrating no valdiazen was produced when feeding *ΔhamG* with ^{15}N-**4**, stacked above the positive result from *ΔhamC* + ^{15}N-**1**.

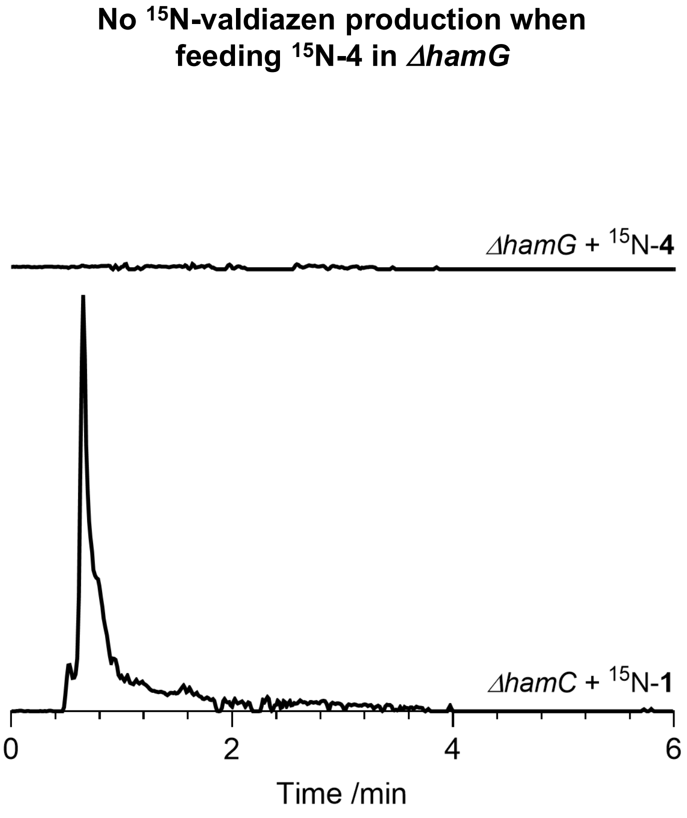

Supplementary Fig 4. Waterfall plots of EIC traces demonstrating the production of ^{15}N-labelled fragin and valdiazen from *ΔhamA, ΔhamB and ΔhamE* mutants.

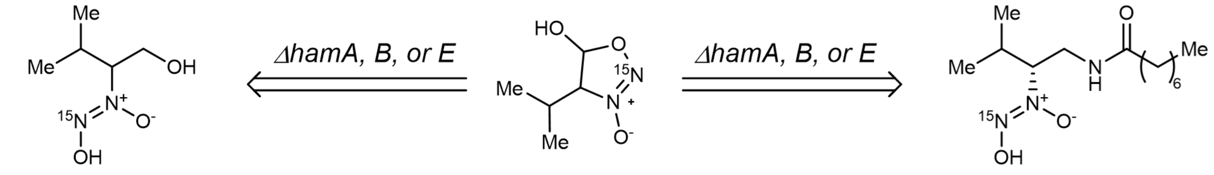

rac-^{15}N-valdiazen

^{15}N-**1**

(*R*)-^{15}N-fragin

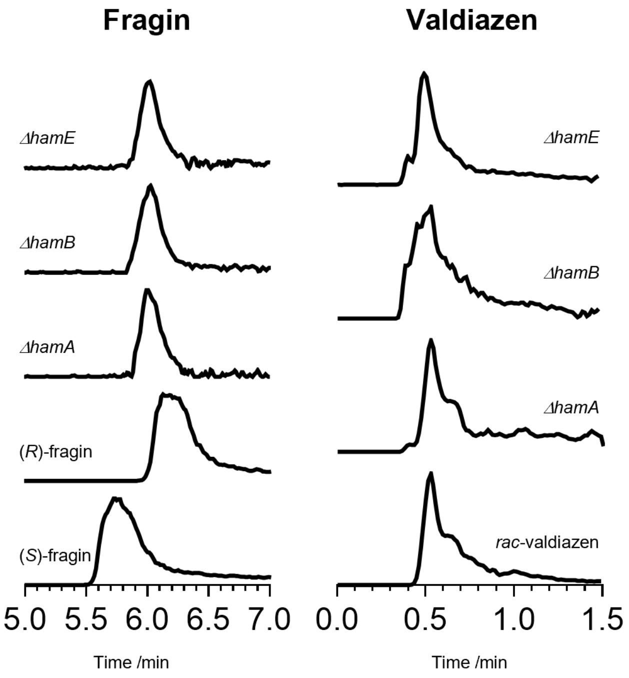

Supplementary Fig 5. Production of (*R*)-**4** in the *ΔhamF* supernatant and lysate. Left: EIC traces confirmed the stereoselective production of (*R*)-**4** in the *ΔhamF* mutant. Right: HRMS/HRMS spectra of FDAA-(*R*)-**4**.

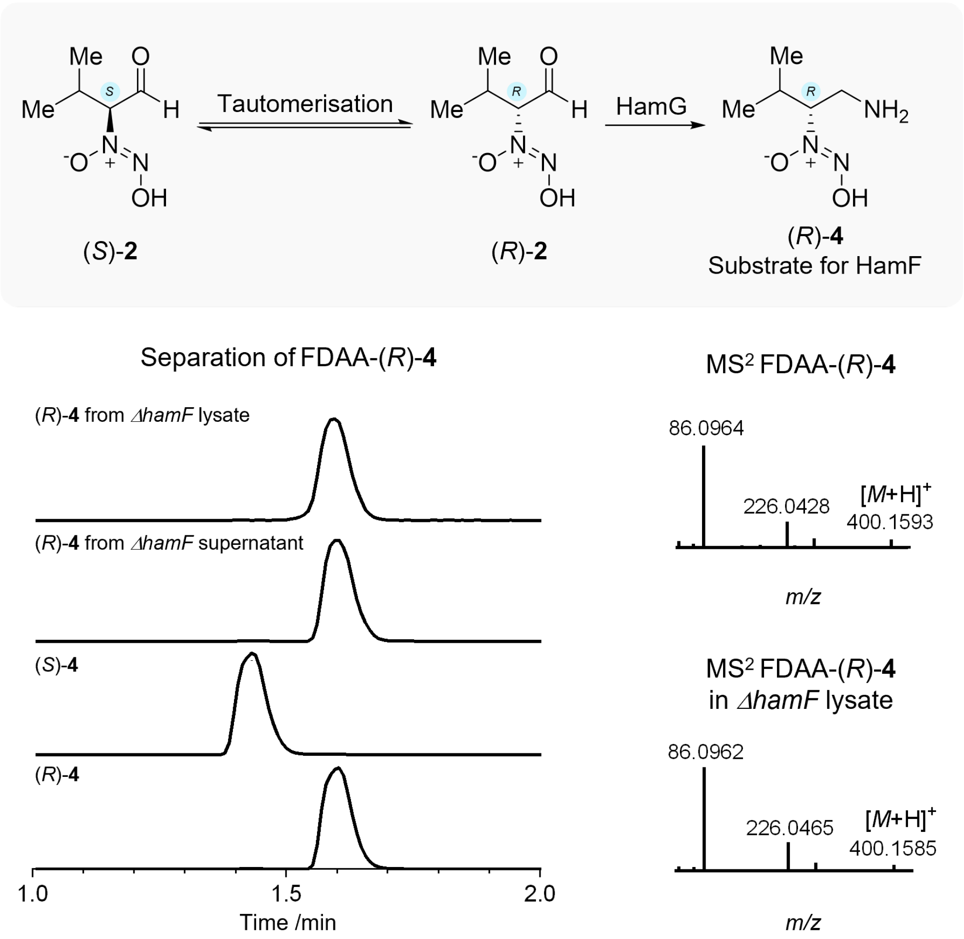

Synthesis of 1,1-dimethoxy-3-methylbutan-2-one (7)

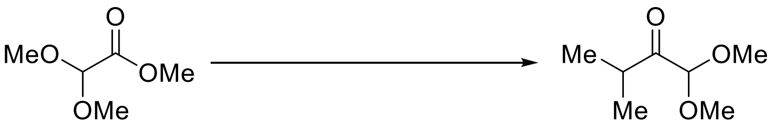

A solution of $AlMe_3$ in hexanes (2 M, 20.4 mL, 40.8 mmol, 2.5 eq.) was slowly added under a N_2 atmosphere to *N*,*O*-dimethylhydroxylamine hydrochloride (3.98 g, 40.8 mmol, 2.5 eq.) in dry DCM (22 mL) at 0 °C, then the reaction mixture was allowed to warm up to r.t. and stirred for 1 hour at r.t.. The reaction mixture was cooled to 0 °C and a solution of methyl dimethoxyacetate (**6**, 2.00 mL, 16.3 mmol, 1 eq.) in dry DCM (17.5 mL) was slowly added over 2 hours. The reaction mixture was allowed to warm up to r.t. and stirred for another 2 hours at this temperature. TLC monitoring (50% EtOAc in hexanes) indicated full consumption of the starting material. The reaction mixture was then slowly poured into an ice-cold 0.5 M aq. HCl soln. (22 mL) and stirred at 0 °C for 30 min before the layers were separated. The aqueous phase was extracted with diethyl ether (2 × 20 mL). The combined organic phases were dried over anhydrous Na_2SO_4, filtered, and concentrated *in vacuo* (minimum 200 mbar, 40 °C) to give the Weinreb amide (2.65 g) as a light-yellow oil. TLC in neat EtOAc (R_f = 0.6, faint under UV, oxidisable with $KMnO_4$) showed a very clean profile with minor impurity above the desired product spot. The compound is volatile under a high stream of nitrogen gas.

To a solution of the Weinreb amide (2.65 g) in dry THF (55 mL) at −78 °C under N_2, isopropylmagnesium bromide in 2-MeTHF (3 M, 7.60 mL, 22.7 mmol, 1.3 eq.) was added dropwise. The reaction mixture was then warmed to r.t. and stirred for 2 hours. TLC monitoring in neat EtOAc and 20% EtOAc in hexanes indicated the completion of the reaction after 2 hours. The reaction was then cooled to 0 °C and quenched with sat. aq. NH_4Cl soln. (20 mL). The reaction was allowed to warm to r.t. and extracted with diethyl ether (3 × 50 mL). The combined organic layers were washed with brine (50 mL), dried over anhydrous Na_2SO_4, filtered, and concentrated *in vacuo* to give a colourless oil. The residue (2.11 g) was loaded onto silica gel (250 mL) and eluted with 20% diethyl ether in hexanes (2 L) to give 1,1-dimethoxy-3-methylbutan-2-one (1.34 g, 9.17 mmol, 56% over 2 steps) as a colourless oil. The compound was volatile and evaporated below 50 mbar at 40 °C on a rotary evaporator. The analytical data match those reported in the literature.[12]

$\mathbf{R_f}$ = 0.50 (SiO_2, 20% diethyl ether in hexanes, $KMnO_4$ stain).

^{1}H NMR (400 MHz, $CDCl_3$) δ = 4.61 (d, J = 0.4 Hz, 1H), 3.39 (d, J = 0.4 Hz, 6H), 3.01 (pd, J = 6.9, 0.4 Hz, 1H), 1.10 (d, J = 0.4 Hz, 3H), 1.09 (d, J = 0.5 Hz, 3H).

^{13}C NMR (101 MHz, $CDCl_3$) δ = 209.25, 103.12, 54.60, 35.99, 18.37.

ESI-HRMS (MeOH + NaI): *m/z* 169.08352 ($C_7H_{14}NaO_3^+$; $[M+Na]^+$; calc. 169.08352).

^{1}H NMR (400 MHz, $CDCl_3$)

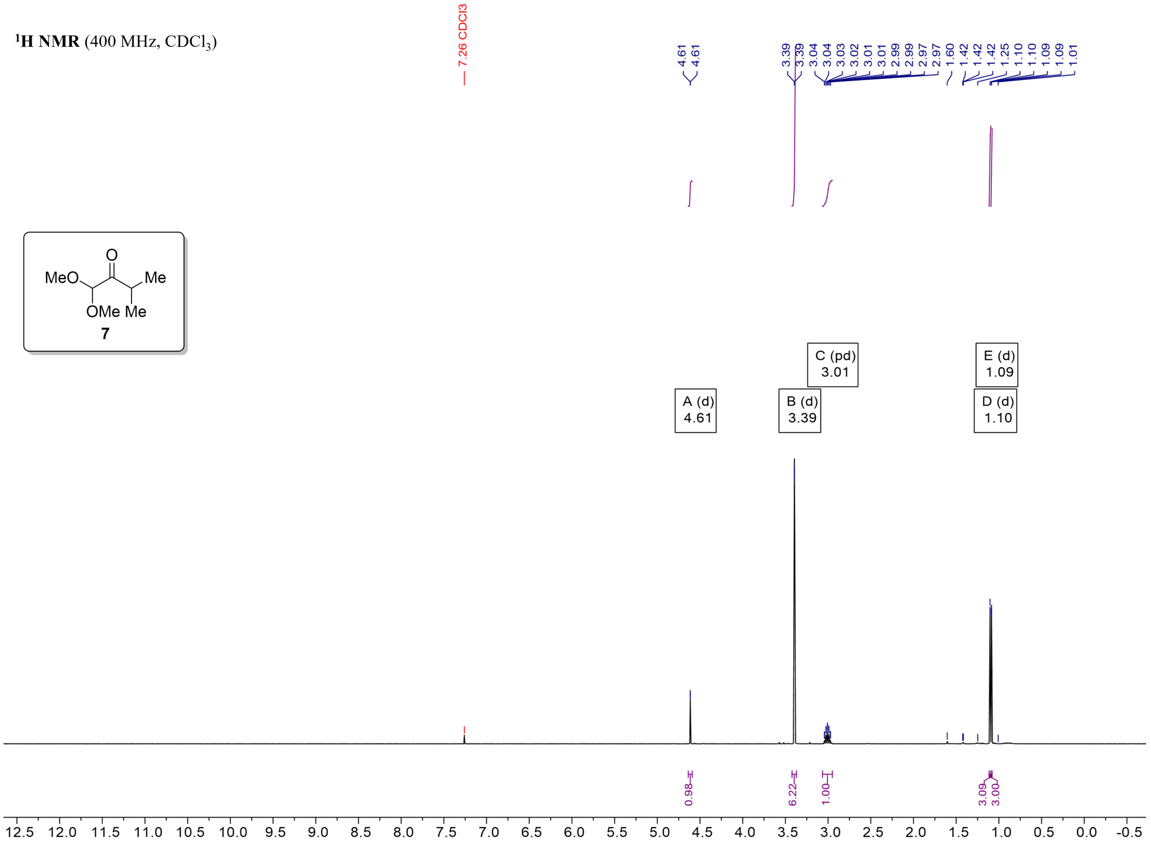

^{13}C NMR (101 MHz, $CDCl_3$)

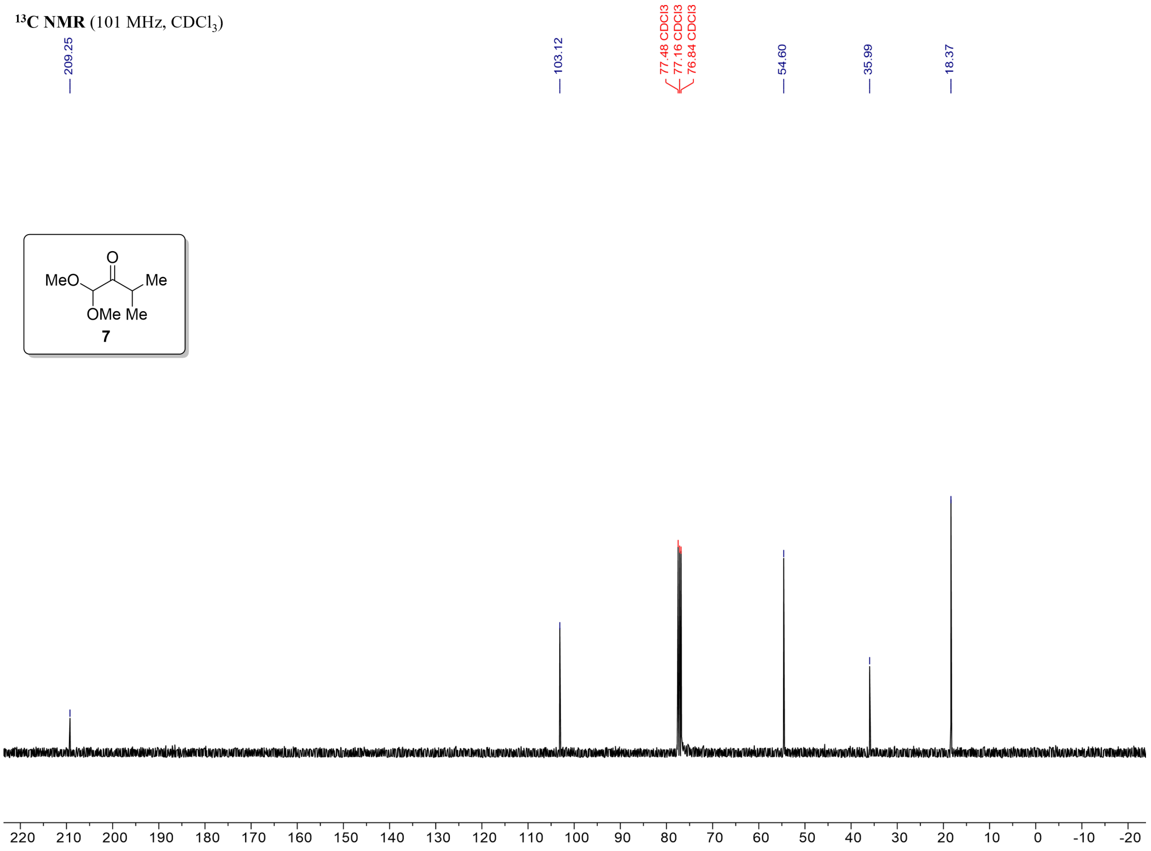

Synthesis of (*E*) and (*Z*)-1,1-dimethoxy-3-methylbutan-2-one oximes (8)

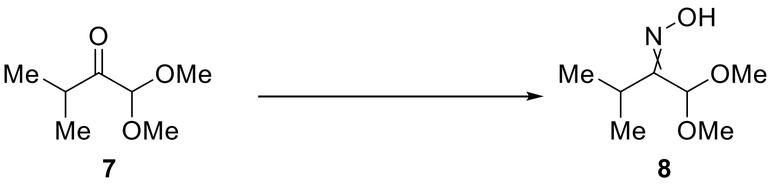

To a mixture of hydroxylamine hydrochloride (359 mg, 5.17 mmol, 1.2 eq.) and NaOAc (707 mg, 8.62 mmol, 2 eq.) in EtOH (30 mL) and water (13 mL) was added ketone **7** (630 mg, 4.31 mmol, 1 eq.) in a single portion. The mixture was then heated to 70 °C for 3 hours. TLC monitoring (20% EtOAc in hexanes) indicated full consumption of the starting material, and two spots detectable by $KMnO_4$ stain were observed. The mixture was cooled to r.t., concentrated *in vacuo* and diluted with water (10 mL). The aqueous layer was extracted with EtOAc (3 × 20 mL). The combined organic phases were dried over anhydrous Na_2SO_4, filtered, and concentrated *in vacuo* to give a colourless oil. The residue (760 mg) was loaded onto silica gel (50 mL) and the compounds were eluted with 20% EtOAc in hexanes to give the corresponding oximes as a colourless oil consisting of a mixture of *E*/*Z* isomers (545 mg, 3.38 mmol, 78%) in a 1:2.7 ratio based on ^{1}H NMR.

R_f = 0.57 and 0.36 (SiO_2, 20% EtOAc in hexanes, $KMnO_4$ stain).

^{1}H NMR (400 MHz, $CDCl_3$) δ = 5.50 (s, 1H), 4.67 (s, 3H), 3.45 (s, 6H), 3.37 (s, 17H), 3.07 (p, J = 7.1 Hz, 3H), 2.79 (p, J = 6.9 Hz, 1H), 1.22 (d, J = 7.1 Hz, 16H), 1.14 (d, J = 6.9 Hz, 6H).

^{13}C NMR (101 MHz, $CDCl_3$) δ = 162.22, 160.19, 104.00, 97.95, 55.72, 54.42, 28.36, 26.55, 21.20, 18.69.

FTIR $\tilde{\nu}$ (cm^{-1}) = 3282m, 2967m, 2935m, 2877m, 2833m, 1701w, 1547w, 1453m, 1383m, 1360m, 1214m, 1190m, 1155w, 1107s, 1068s, 1014m, 931s, 901s, 828m, 787m, 764m, 596w, 568w.

ESI-HRMS (MeOH): *m/z* 184.09470 ($C_7H_{15}O_3NNa^+$; $[M+Na]^+$; calc. 184.09441).

^{1}H NMR (400 MHz, $CDCl_3$)

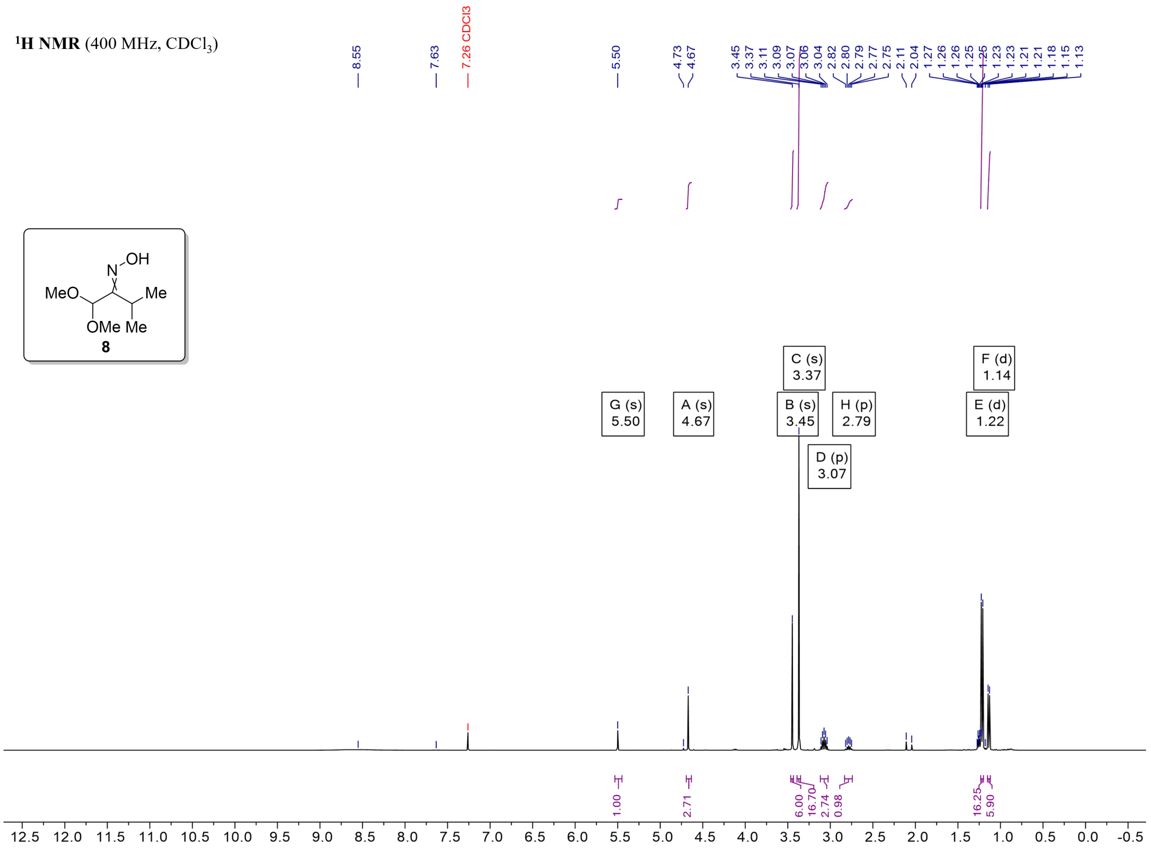

^{13}C NMR (101 MHz, $CDCl_3$)

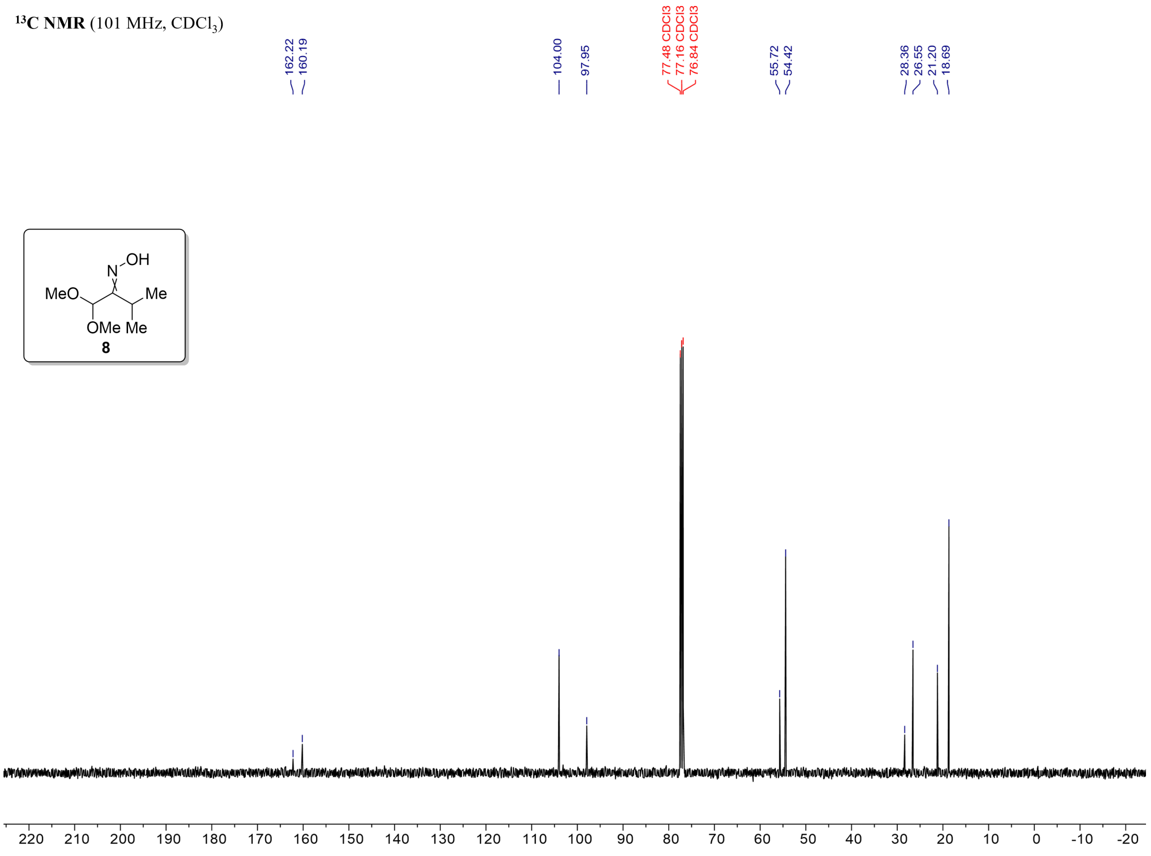

Synthesis of (*Z*)-1-(1,1-dimethoxy-3-methylbutan-2-yl)-2-hydroxydiazene 1-oxide-2-^{15}N (9)

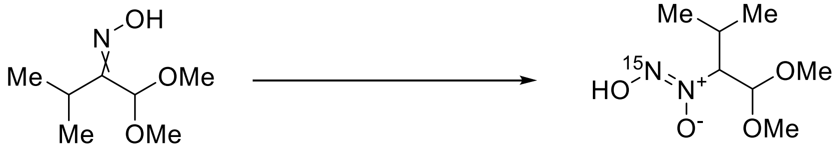

8 **9**

To a stirred solution of oximes **8** (1.06 g, 6.58 mmol, 1 eq.) in dry ethanol (21 mL) under a N_2 atmosphere at 0 °C, $NaBH_3CN$ (522 mg, 0.744 mmol, 1.2 eq.) was added followed by dropwise addition of HCl in ethanol (1.25 M, 6.40 mL, 8.00 mmol, 1.2 eq.). The reaction mixture was then stirred at r.t. for 1 hour. UHPLC-MS analysis indicated full conversion. The reaction mixture was concentrated *in vacuo*, water (20 mL) and EtOAc (20 mL) were added to the residue before neutralising with sat. aq. Na_2CO_3 soln. (2 mL). The aqueous layer was extracted with EtOAc (5 × 20 mL). The combined organic layers were dried over anhydrous Na_2SO_4, filtered, and concentrated *in vacuo* to give the intermediate hydroxylamine (1.09 g, quant.) as a light pink oil. UHPLC-MS analysis supported the desired mass.

To a solution of the hydroxylamine (200 mg, 1.23 mmol, 1 eq.) in a 1:1 EtOH/H_2O mixture (2 mL) at 0 °C was added 1 M aq. HCl soln. (1.40 mL, 1.40 mmol, 1.1 eq.) dropwise. The mixture was degassed with Ar for 10 mins. In a separate flask, a solution of $Na^{15}NO_2$ (95.0 mg, 1.36 mmol, 1.1 eq.) in water (1 mL) was also degassed with Ar argon for 10 mins before being added dropwise to the hydroxylamine solution at 0 °C. The reaction was then stirred at 0 °C for 30 minutes. UHPLC-MS analysis indicated full conversion of the starting material and the formation of the desired product. The reaction mixture was then treated with sat. aq. $NaHCO_3$ soln. (4 mL) and freshly distilled diethyl ether (5 mL). The aqueous phase was washed with freshly distilled diethyl ether (3 × 5 mL) before being acidified with 1 M aq. HCl soln. (6 mL). The aqueous phase was then extracted with diethyl ether (5 × 10 mL). The combined organic phases were dried over anhydrous Na_2SO_4, filtered, and concentrated *in vacuo* to give the diazeniumdiolate acetal **9** (130 mg, 0.673 mmol, 55% over 2 steps) as a light-yellow oil.

Odor: Pungent spicy smell

^{1}**H NMR** (400 MHz, Methanol-*d$_4$*) δ = 4.85 (d, *J* = 7.9 Hz, 1H), 4.16 (ddd, *J* = 7.9, 6.0, 1.9 Hz, 1H), 3.44 (s, 3H), 3.38 (s, 3H), 2.27 (pd, *J* = 6.9, 5.9 Hz, 1H), 1.03 (d, *J* = 6.9 Hz, 3H), 0.99 (d, *J* = 7.0 Hz, 3H).

^{13}**C NMR** (101 MHz, Methanol-*d$_4$*) δ = 103.62, 78.92, 55.73, 54.99, 29.47, 19.73, 18.24.

FTIR $\tilde{\nu}$ (cm^{-1}) = 2968m, 2939m, 2838w, 1457m, 1392m, 1371m, 1281m, 1192m, 1150m, 1119m, 1055s, 992m, 971m, 945m, 909m, 855w, 829m, 781m, 702m, 668m, 593m, 531w, 479w.

ESI-HRMS (MeCN): *m/z* 216.09728 ($C_7H_{16}O_4N^{15}NNa^+$; $[M+Na]^+$; calc. 216.09726).

^{1}H NMR (400 MHz, Methanol-d_4)

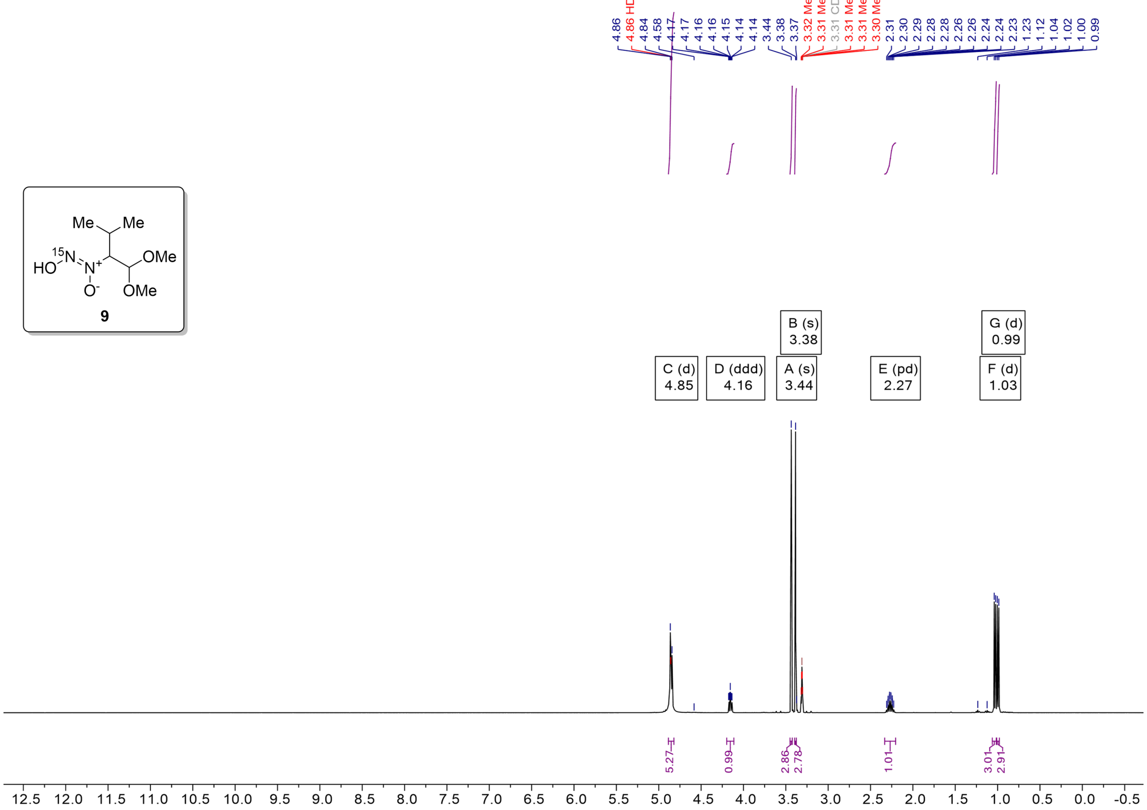

^{13}C NMR (101 MHz, Methanol-d_4)

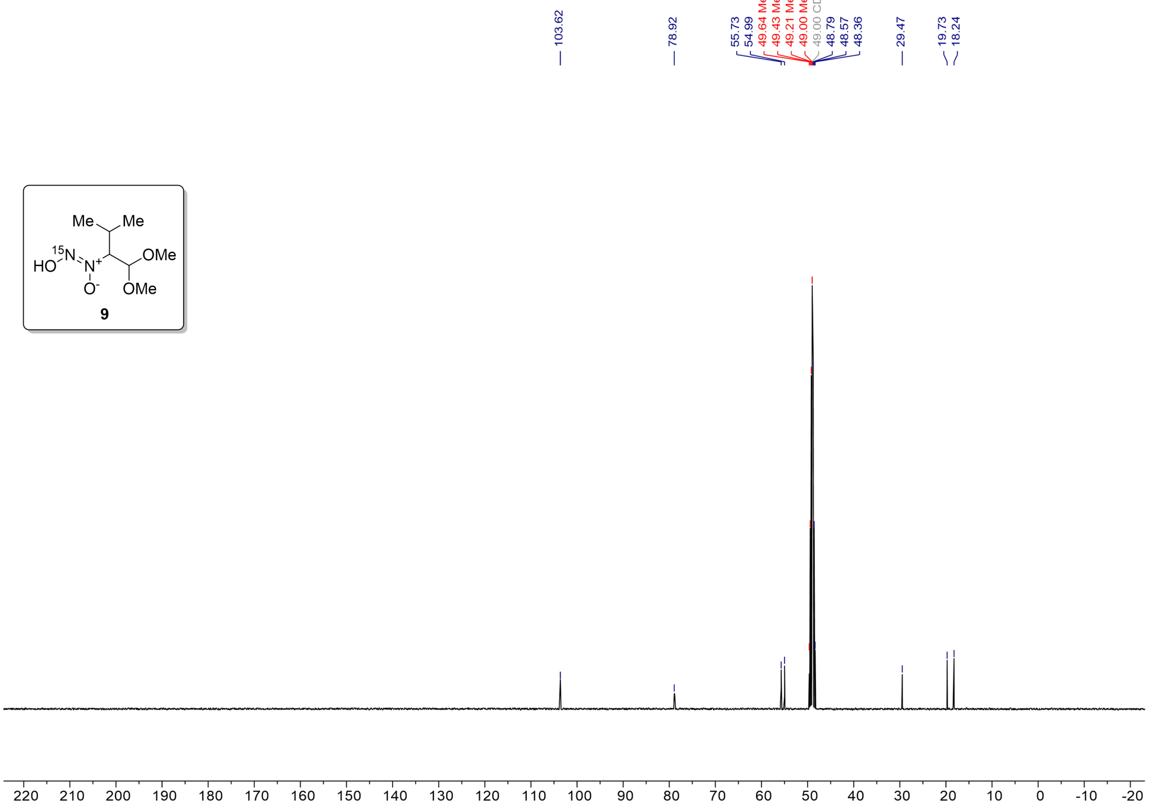

Synthesis of 5-hydroxy-4-isopropyl-4,5-dihydro-1,2,3-oxadiazole 3-oxide-2-^{15}*N* (^{15}N-1)

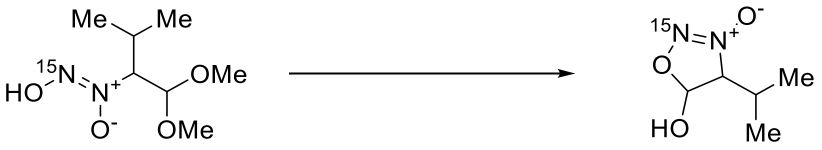

9 ^{15}N-**1**

The diazeniumdiolate acetal **9** (49.0 mg, 0.254 mmol, 1 eq.) was treated with 10% aq. HCl soln. (1.20 mL, 3.81 mmol, 15 eq.) and the reaction mixture was stirred at 50 °C for 3 hours. The mixture was concentrated *in vacuo* to less than 0.5 mL before being purified by preparative HPLC (*Phenomenex Synergi*^{TM} 10 µm Hydro-RP 80 Å, 250 mm × 21.2 mm, 20 mL/min, 0%–50% MeCN + 0.1% formic acid in Milli-Q® water + 0.1% formic acid, 30 minutes, R_t = 15.0 min.) to give dihydrosydnone *N*-oxide ^{15}N-**1** (17.0 mg, 0.116 mmol, 46%) as a sticky, fluffy white solid. The solid was recrystallised from diethyl ether/cyclopentane for a single crystal X-ray structure analysis.

m.p.= 55.8–60.2 °C

^{1}H NMR (500 MHz, Methanol-d_4) δ = 5.95 (d, J = 2.8 Hz, 1H), 4.15 (dd, J = 4.4, 2.8 Hz, 1H), 2.43 (pd, J = 7.0, 4.4 Hz, 1H), 1.09 (d, J = 7.0 Hz, 4H), 0.98 (d, J = 6.9 Hz, 3H).

^{13}C NMR (126 MHz, Methanol-d_4) δ = 101.29, 85.54, 29.18, 18.01, 17.02.

FTIR $\tilde{\nu}$ (cm^{-1}) = 3153m, 2973w, 1441m, 1397m, 1376w, 1343m, 1305m, 1246m, 1147m, 1130m, 1093m, 1008w, 976m, 959s, 933m, 903m, 843m, 807s, 765m, 694m, 612w, 576m, 501m, 477w.

ESI-HRMS (MeCN): *m/z* 146.05906 ($C_5H_9O_3N^{15}N^-$; [*M*−H]$^-$; calc. 146.05890).

¹H NMR (500 MHz, Methanol-d_4)

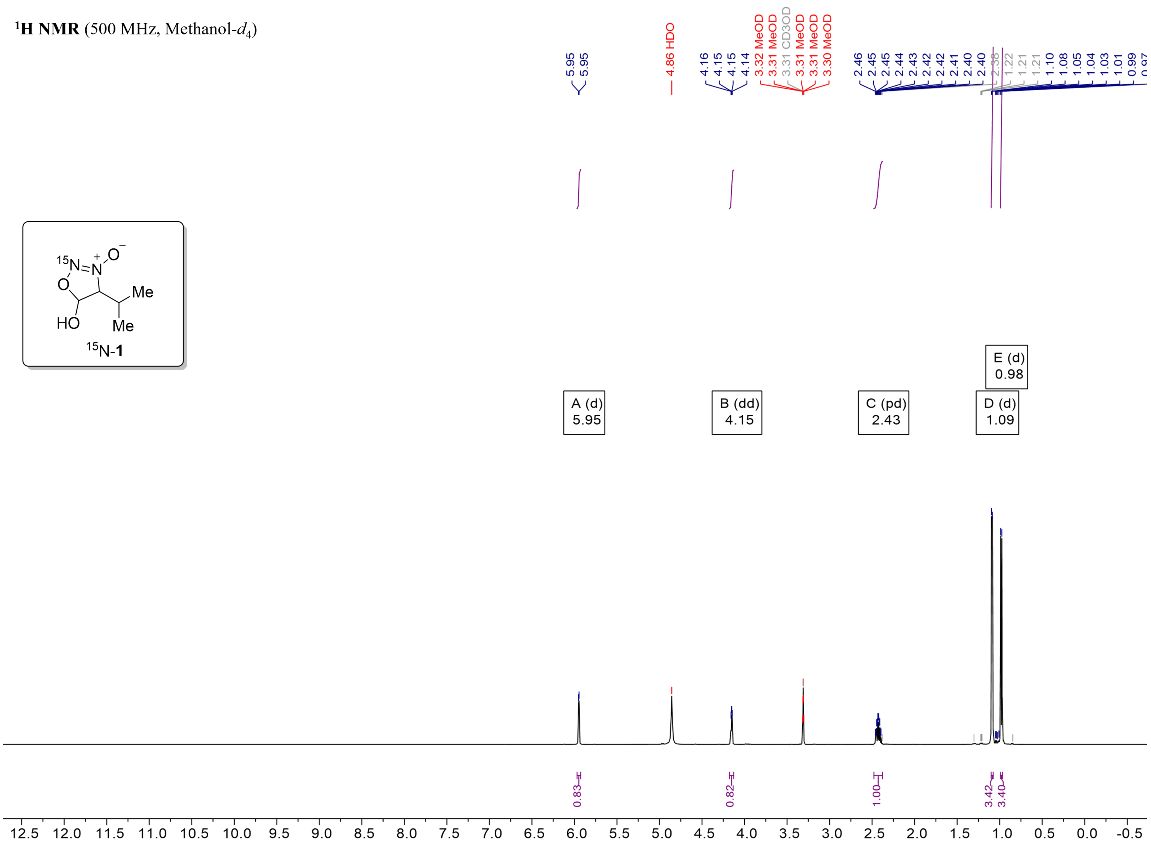

¹³C NMR (126 MHz, Methanol-d_4)

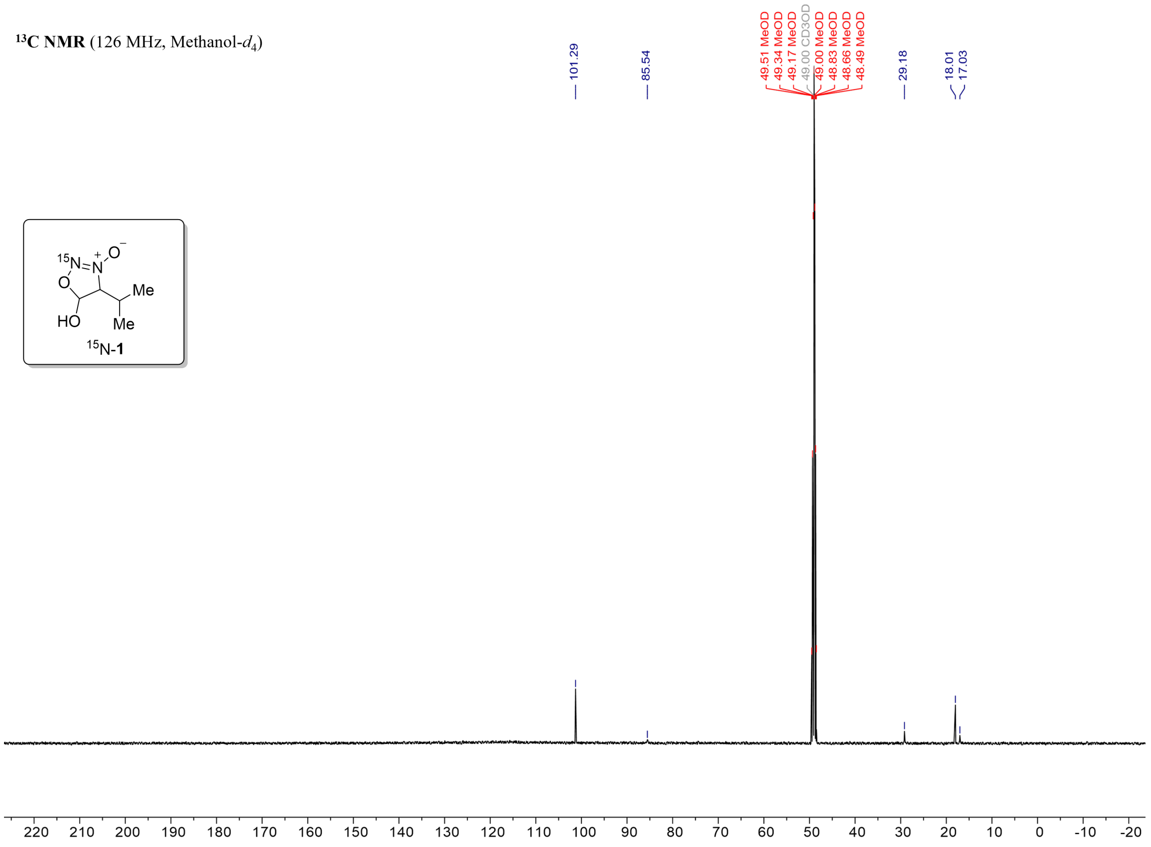

Synthesis of *tert*-butyl (3-methyl-2-oxobutyl) carbamate (11)

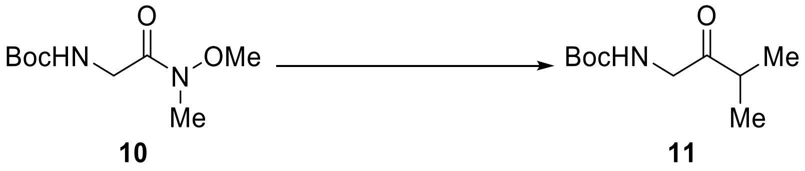

Isopropylmagnesium bromide in 2-MeTHF (3M, 4.00 mL, 12.0 mmol, 1.3 eq.) was added dropwise to a solution of commercially available Weinreb amide **10** (2.00 g, 9.16 mmol, 1 eq.) in dry THF (29 mL) at −78 °C under N_2. The reaction mixture was then allowed to warm to r.t. and stirred for 2 hours. TLC monitoring was carried out in neat EtOAc and 20% EtOAc in hexanes using $KMnO_4$ stain. Upon completion, the reaction then was cooled to 0 °C and quenched with sat. aq. NH_4Cl soln. (10 mL). The reaction mixture was allowed to warm to r.t. and extracted with diethyl ether (3 × 50 mL). The combined organic layers were washed with brine (20 mL), dried over Na_2SO_4, filtered, and concentrated *in vacuo* to give the ketone **11** (889 mg, 4.42 mmol, 49%) as a colourless oil. The analytical date match those reported in the literature.[13]

R_f = 0.50 (SiO_2, 20% EtOAc in hexanes, $KMnO_4$ stain).

^{1}H NMR (400 MHz, $CDCl_3$) δ = 5.24 (s, 1H), 4.09 (d, J = 4.6 Hz, 2H), 2.64 (hept, J = 6.9 Hz, 1H), 1.45 (s, 9H), 1.15 (s, 3H), 1.13 (s, 3H).

^{13}C NMR (101 MHz, $CDCl_3$) δ 209.49, 79.90, 48.56, 38.87, 28.48, 18.36.

ESI-HRMS (MeCN): *m/z* 224.12581 ($C_{10}H_{19}O_3NNa^+$; $[M+Na]^+$; calc. 224.12571).

^{1}**H NMR** (400 MHz, $CDCl_3$)

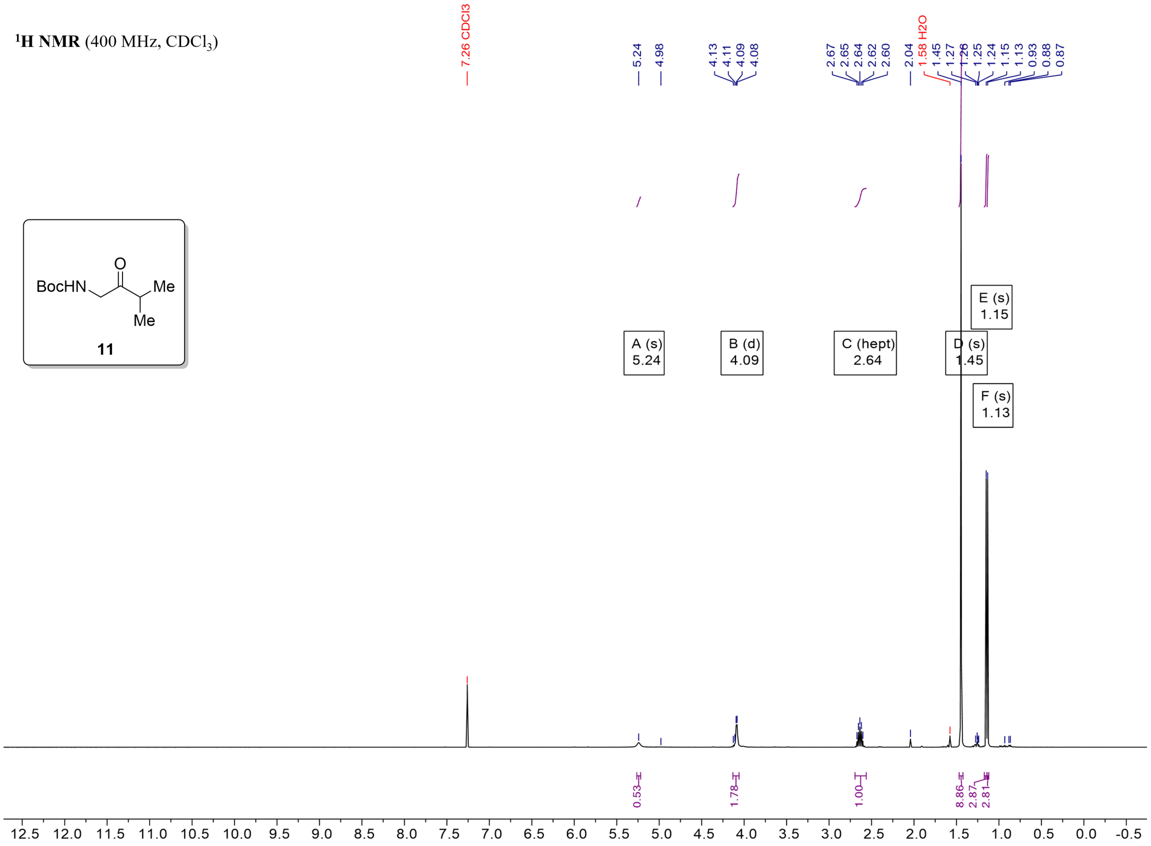

^{13}**C NMR** (101 MHz, $CDCl_3$)

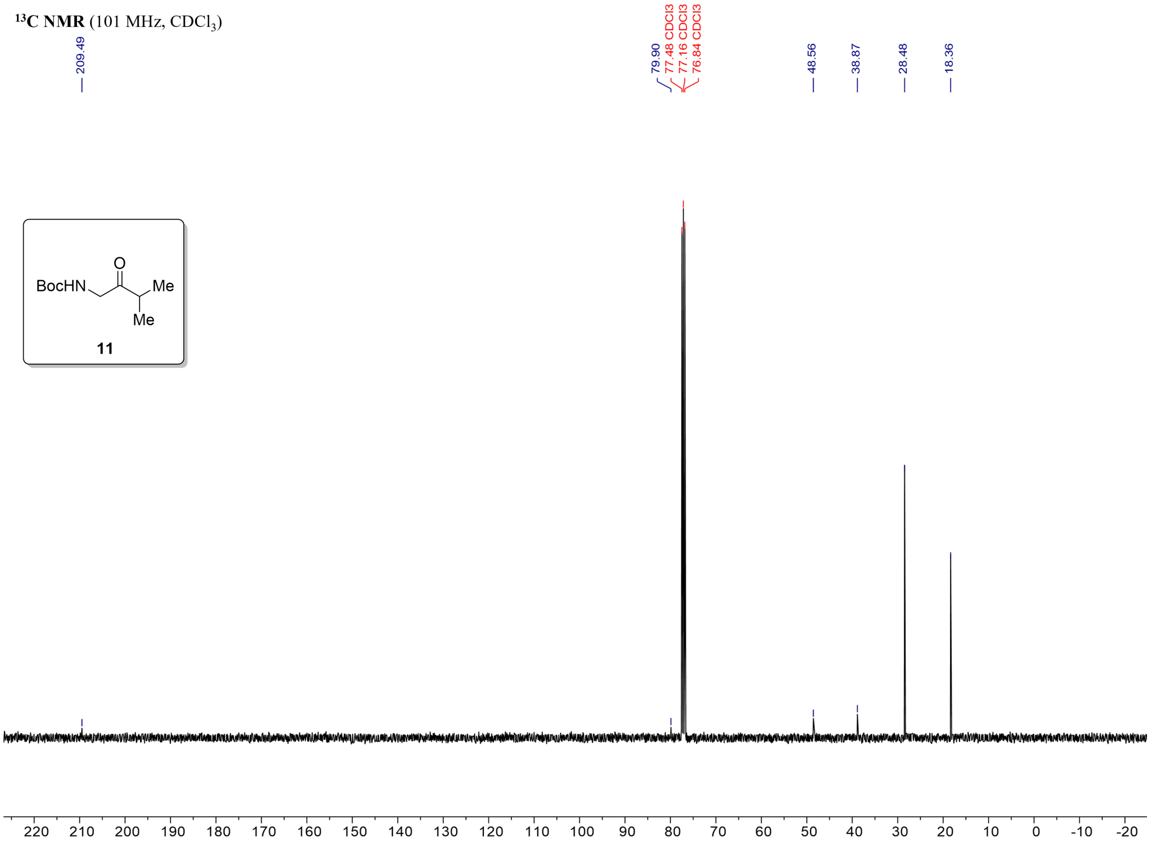

Synthesis of *tert*-butyl (2-(hydroxyimino)-3-methylbutyl) carbamate (12)

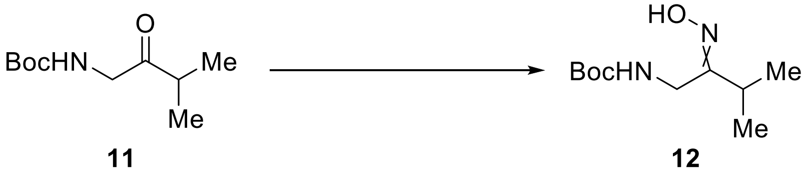

To a mixture of hydroxylamine hydrochloride (380 mg, 5.30 mmol, 1.2 eq.) and KOAc (868 mg, 8.84 mmol, 2 eq.) in EtOH (30 mL) and water (13 mL) was added ketone **11** (889 mg, 4.42 mmol, 1 eq.) in a single portion. The reaction mixture was then heated to 70 °C for 3 hours. TLC monitoring in 20% EtOAc in hexanes indicated full consumption of the starting material and two spots detectable by $KMnO_4$ staining. The reaction mixture was cooled to r.t., concentrated *in vacuo*, and diluted with water (10 mL). The aqueous layer was extracted with EtOAc (3 × 20 mL). The combined organic phases were dried over anhydrous Na_2SO_4, filtered, and concentrated *in vacuo* to give a colourless oil. The residue (1.22 g) was loaded onto silica gel (120 mL) and eluted with 20% EtOAc in hexanes to give the corresponding oximes as a white solid consisting of a mixture of *E*/*Z* isomers (850 mg, 3.93 mmol, 89%), in a 1:1.8 ratio based on ^{1}H NMR.

R_f = 0.38 and 0.30 (SiO_2, 20% EtOAc in hexanes, $KMnO_4$ stain).

m.p.= 94.5–98.2 °C

^{1}H NMR (400 MHz, $CDCl_3$) δ = 8.26 (s, 1H), 7.59 (s, 1H), 5.24 (s, 1H), 5.15 (s, 1H), 3.97 (d, J = 6.3 Hz, 3H), 3.90 (d, J = 5.0 Hz, 2H), 3.39 (p, J = 7.1 Hz, 1H), 2.59 (hept, J = 6.9 Hz, 2H), 1.45 (s, 23H), 1.12 (d, J = 6.8 Hz, 10H), 1.09 (d, J = 7.0 Hz, 6H).

^{13}C NMR (101 MHz, $CDCl_3$) δ = 163.26, 155.77, 79.75, 40.06, 36.39, 32.90, 28.52, 25.86, 19.62, 18.74.

FTIR $\tilde{\nu}$ (cm^{-1}) = 3323m, 2982w, 2965w, 1704w, 1681s, 1539s, 1454m, 1434m, 1390m, 1365m, 1341w, 1279s, 1249s, 1166s, 1147s, 1110m, 1038m, 1020m, 951m, 937s, 912m, 899w, 887m, 858m, 771w, 752m, 722m, 677s, 597m.

ESI-HRMS (MeCN): *m/z* 217.15459 ($C_{10}H_{21}O_3N_2^+$; $[M+H]^+$; calc. 217.15467).

¹H NMR (400 MHz, $CDCl_3$)

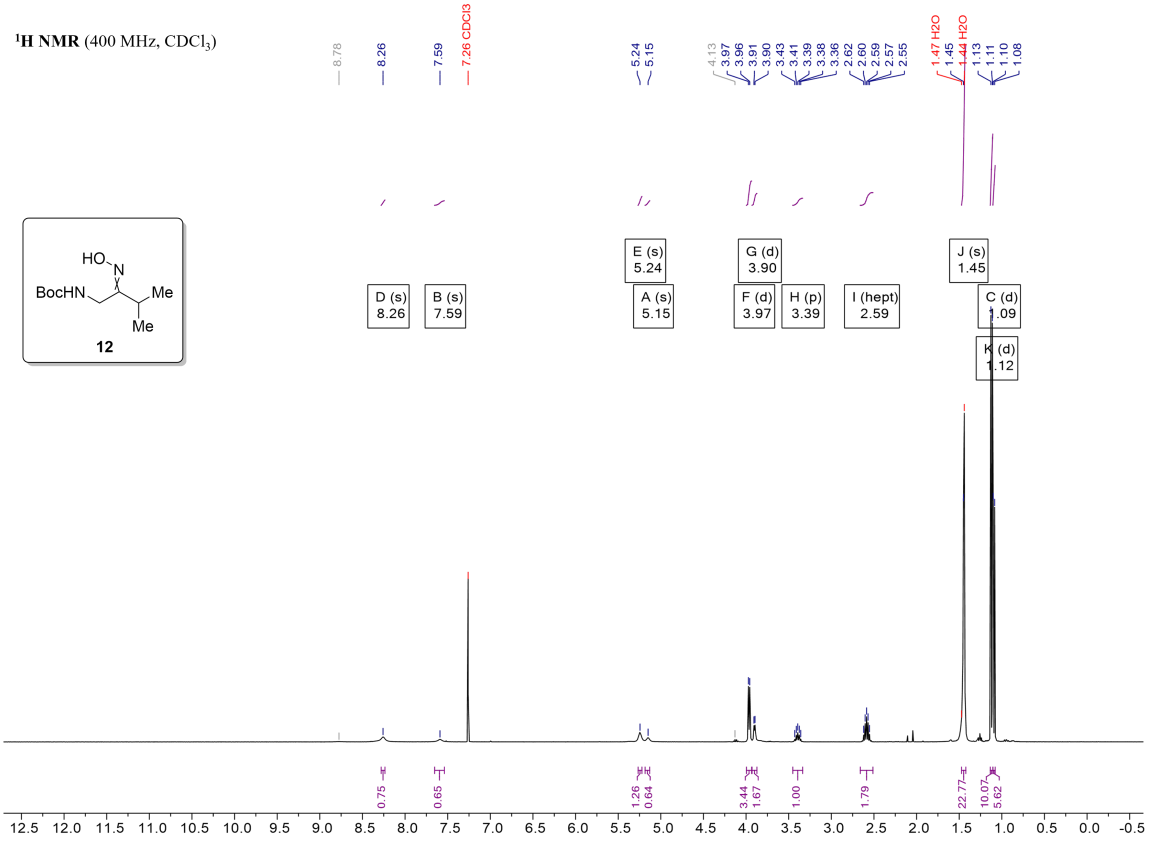

¹³C NMR (101 MHz, $CDCl_3$)

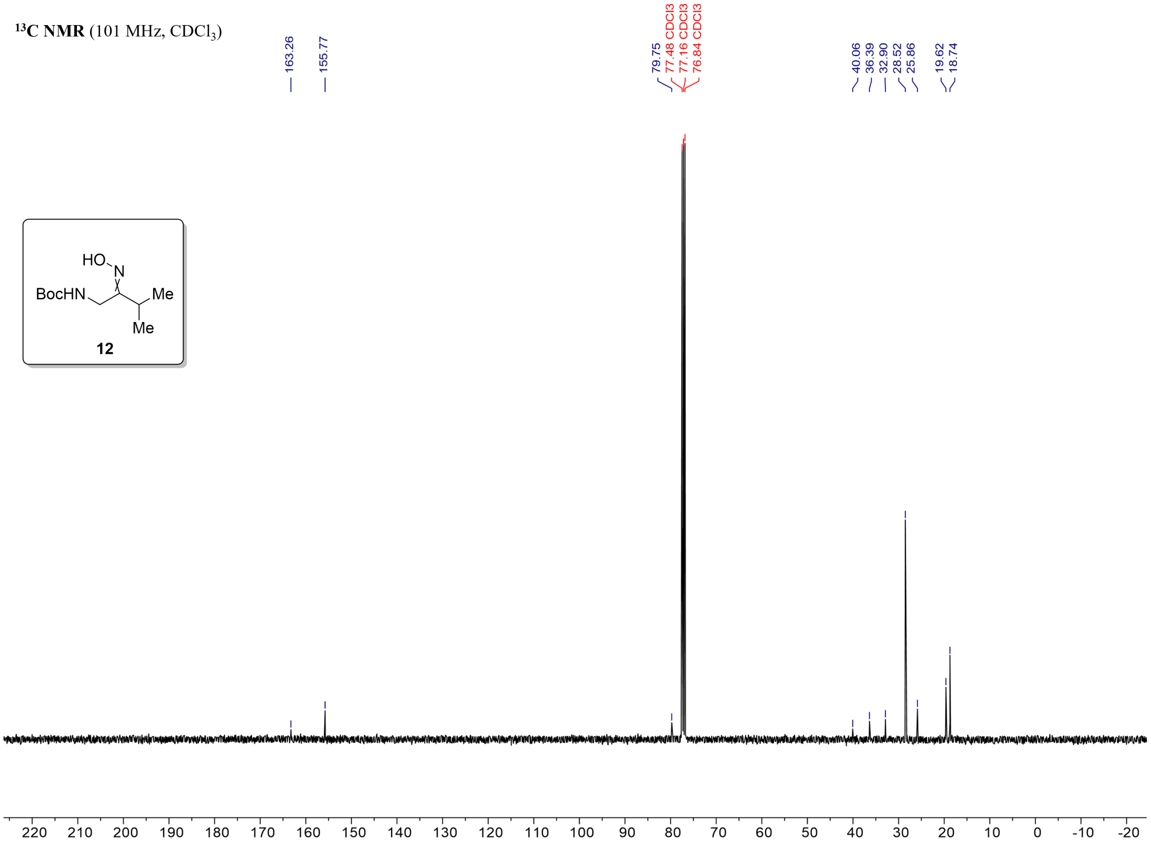

Synthesis of (*Z*)-1-(1-amino-3-methylbutan-2-yl)-2-hydroxydiazene 1-oxide-2-^{15}N (^{15}N-4)

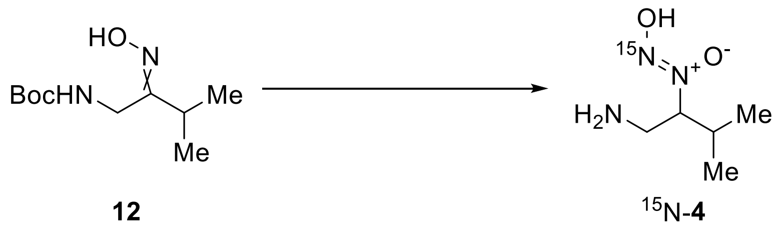

To a stirred solution of oxime **12** (207 mg, 0.957 mmol) in dry ethanol (3.00 mL) under N_2 at 0 °C, $NaBH_3CN$ (180 mg, 2.87 mmol, 3 eq.) was added, followed by dropwise addition of ethanolic HCl soln. (1.25 M, 2.30 mL, 2.88 mmol) at 0 °C. The reaction mixture was then warmed to r.t. and stirred for 1 hour. All starting material was consumed based on TLC monitoring (20% EtOAc in hexanes). UHPLC-MS indicated full consumption of the starting material and the formation of the desired product. The mixture was treated with EtOAc (10 mL) and sat. aq. Na_2CO_3 soln. (3 mL). The aqueous layer was extracted with EtOAc (4 × 10 mL). The combined organic layers were washed with brine (10 mL), dried over anhydrous Na_2SO_4, filtered, and concentrated *in vacuo* to give the corresponding hydroxylamine (201 mg) as a colourless gum.

To a solution of the hydroxylamine (201 mg, 0.921 mmol, 1 eq.) in DCM (1.70 mL) at 0 °C was added TFA (711 µL, 9.57 mmol, 10 eq.). The reaction mixture was stirred for 1 hour at r.t.. UHPLC-MS analysis indicated full consumption of the starting material and the formation of the desired product as a TFA salt. The mixture was concentrated *in vacuo*, the residue was taken up in water (1 mL) and filtered through a DSC-18 cartridge (5 g bed) eluting with water to give the corresponding amine (358 mg) as a TFA salt.

To a solution of the above amine (358 mg, 1.03 mmol, 1 eq.) in 1:1 EtOH/H_2O (3.00 mL) at 0 °C was added an aq. HCl soln. (1 M, 2.20 mL, 2.20 mmol, 2.2 eq.) dropwise. The mixture was degassed with Ar for 10 minutes. In a separate flask, a solution of $Na^{15}NO_2$ (81.0 mg, 1.16 mmol, 1.1 eq.) in water (1.00 mL) was degassed with Ar for 10 minutes before being added dropwise to the hydroxylamine solution at 0 °C. The reaction was then stirred at 0 °C for 30 minutes. UHPLC-MS analysis indicated full conversion of the starting material and the formation of the desired product eluting at the solvent front. The mixture was concentrated *in vacuo* and treated with acetone (1 mL). The suspension was filtered and the filtrate was concentrated *in vacuo*. The residue was purified by preparative HPLC (*Phenomenex Synergi™* 10 µm Hydro-RP 80 Å, 250 mm × 21.2 mm, 20 mL/min, 100% Milli-Q® water + 0.1% formic acid, 10 minutes, R_t = 6.5 minutes) to give the β-amino diazeniumdiolate ^{15}N-**4** (30.0 mg, 0.202 mmol, 21%) as a white solid. The solid was recrystallised from methanol/diethyl ether for single crystal X-ray structure analysis.

m.p. = 50.2–56.2 °C

^{1}H NMR (400 MHz, Methanol-*d_4*) δ = 4.29 (t, J = 9.4 Hz, 1H), 3.57 (dd, J = 13.8, 9.7 Hz, 1H), 3.36 (dd, J = 13.7, 2.6 Hz, 1H), 2.20 (h, J = 6.8 Hz, 1H), 1.06 (d, J = 6.7 Hz, 3H), 0.94 (d, J = 6.5 Hz, 3H).

^{13}C NMR (101 MHz, Methanol-*d_4*) δ = 39.99, 30.53, 19.16, 19.09.

FTIR $\tilde{\nu}$ (cm^{-1}) = 2973br m, 1668s, 1537m, 1432m, 1377m, 1304w, 1273w, 1179s, 1131s, 1068m, 976m, 925m, 900m, 837m, 799m, 722s, 598w, 518m.

ESI-HRMS (H_2O): *m/z* 149.10495 ($C_5H_{14}O_2N_2{}^{15}N^+$; $[M+H]^+$; calc. 149.10509).

^{1}**H NMR** (400 MHz, Methanol-d_4)

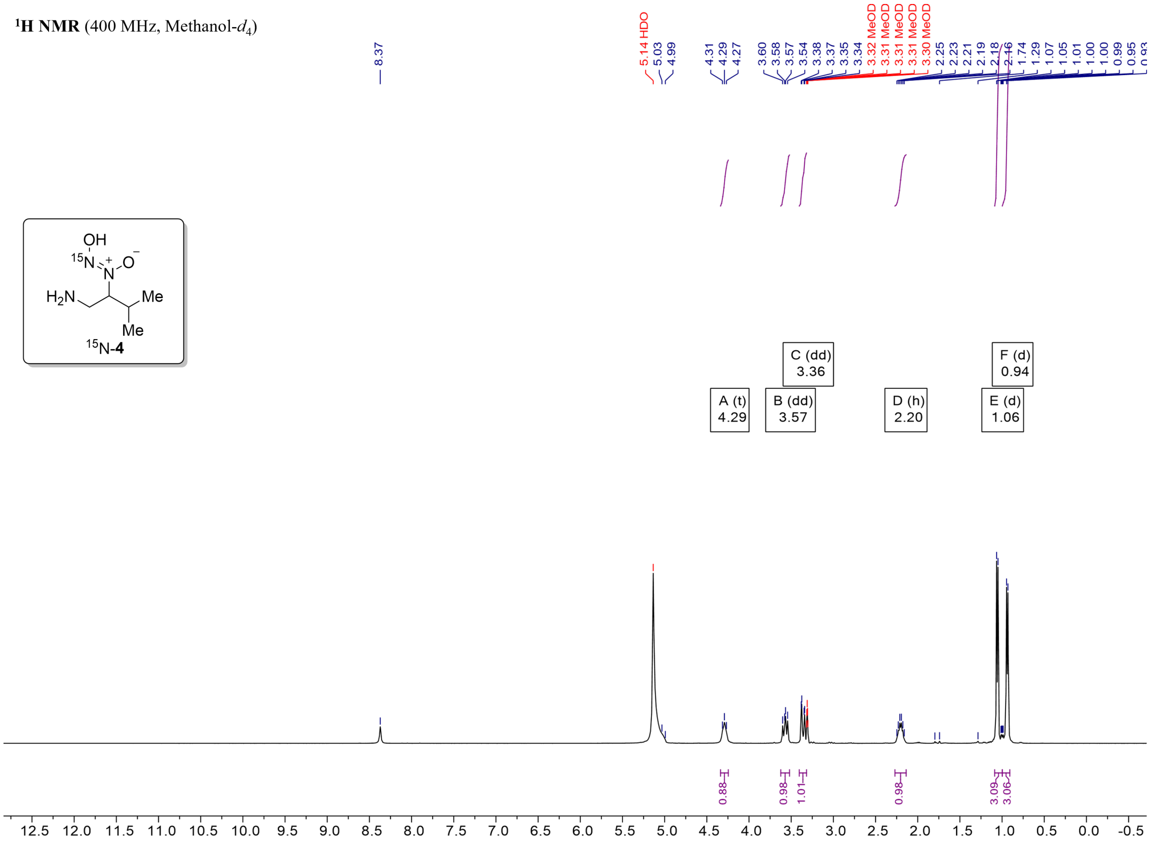

^{13}**C NMR** (101 MHz, Methanol-d_4)

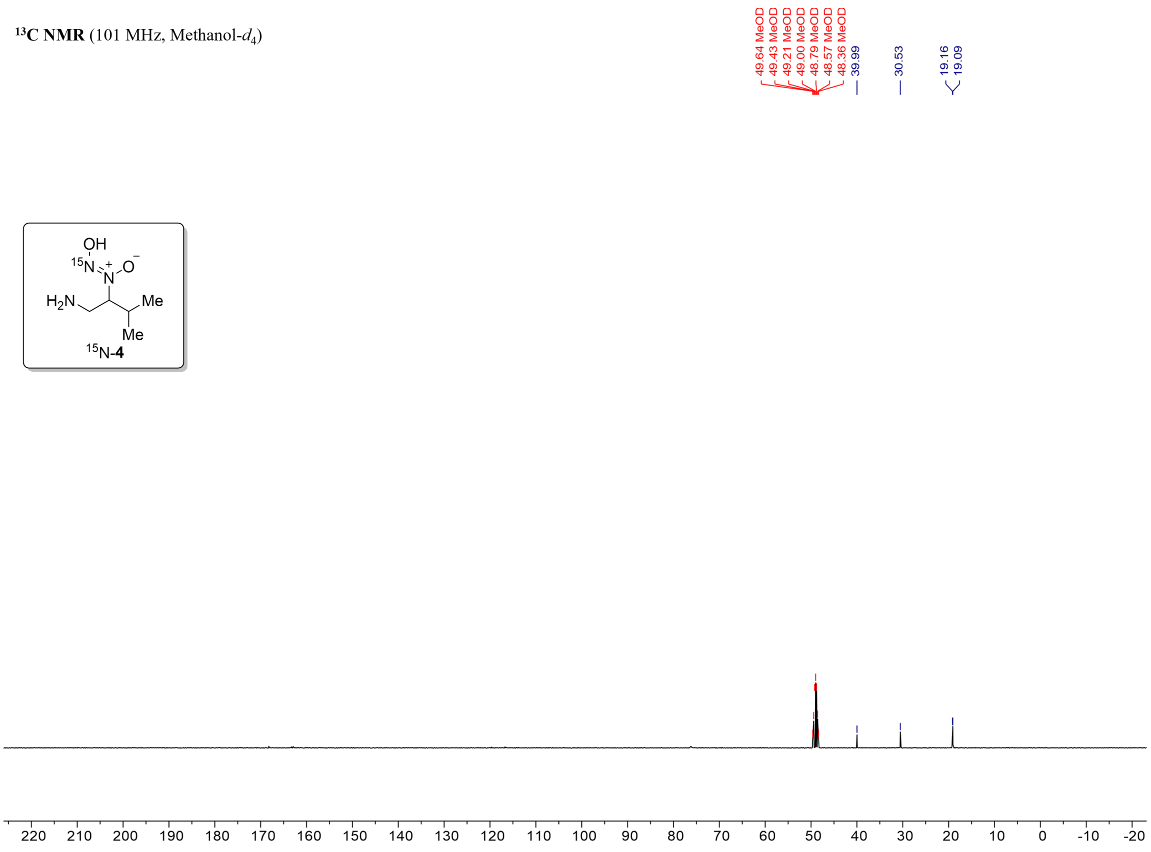

Synthesis (*S*)-2-((benzoyloxy)amino)-3-methylbutan-1-ol (((*S*)-13)

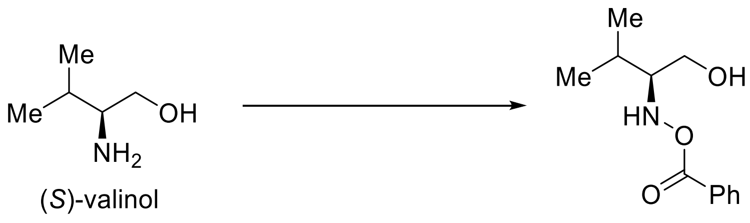

To a suspension of dibenzoylperoxide (611 mg, 2.52 mmol, 1.3 eq.) and K_2HPO_4 (541 mg, 3.10 mmol, 1.6 eq.) in dry THF (5.00 mL) at r.t. was added a solution of (*S*)-valinol (200 mg, 1.94 mmol, 1 eq.) in dry THF (2.30 mL) dropwise. The mixture was stirred at r.t. for 16 hours. The mixture was filtered through a short pad of Celite® with THF. The mixture was concentrated *in vacuo* to give a gummy colourless residue. The residue (655 mg) was loaded onto silica gel (70 mL) and eluted with 10% EtOAc in hexanes (250 mL), then with 30% EtOAc in hexanes (200 mL) to give (*S*)-**13** (317 mg, 1.42 mmol, 73%) as a colourless gum. The analytical data match those reported in the literature.[14]

R_f = 0.35 (SiO_2, 30% EtOAc in hexanes, UV and $KMnO_4$ stain).

^{1}H NMR (400 MHz, $CDCl_3$) δ = 8.01 (dd, J = 8.0, 1.5 Hz, 2H), 7.64 – 7.55 (m, 1H), 7.46 (t, J = 7.7 Hz, 2H), 3.80 (dd, J = 11.6, 3.5 Hz, 1H), 3.64 (dd, J = 11.6, 7.1 Hz, 1H), 2.87 (td, J = 7.3, 3.5 Hz, 1H), 2.00 – 1.87 (m, 1H), 1.11 (d, J = 6.9 Hz, 4H), 1.03 (d, J = 6.8 Hz, 3H).

^{13}C NMR (101 MHz, $CDCl_3$) δ = 167.28, 133.64, 129.54, 128.74, 128.36, 68.60, 60.18, 27.41, 19.83, 19.55.

ESI-HRMS (MeCN): *m/z* 224.12814 ($C_{12}H_{18}O_3N^+$; $[M+H]^+$; calc. 224.12812).

Optical rotation: $[\alpha]_D^{25} = -11.618$ (c = 0.90, $CHCl_3$).

^{1}H NMR (400 MHz, $CDCl_3$)

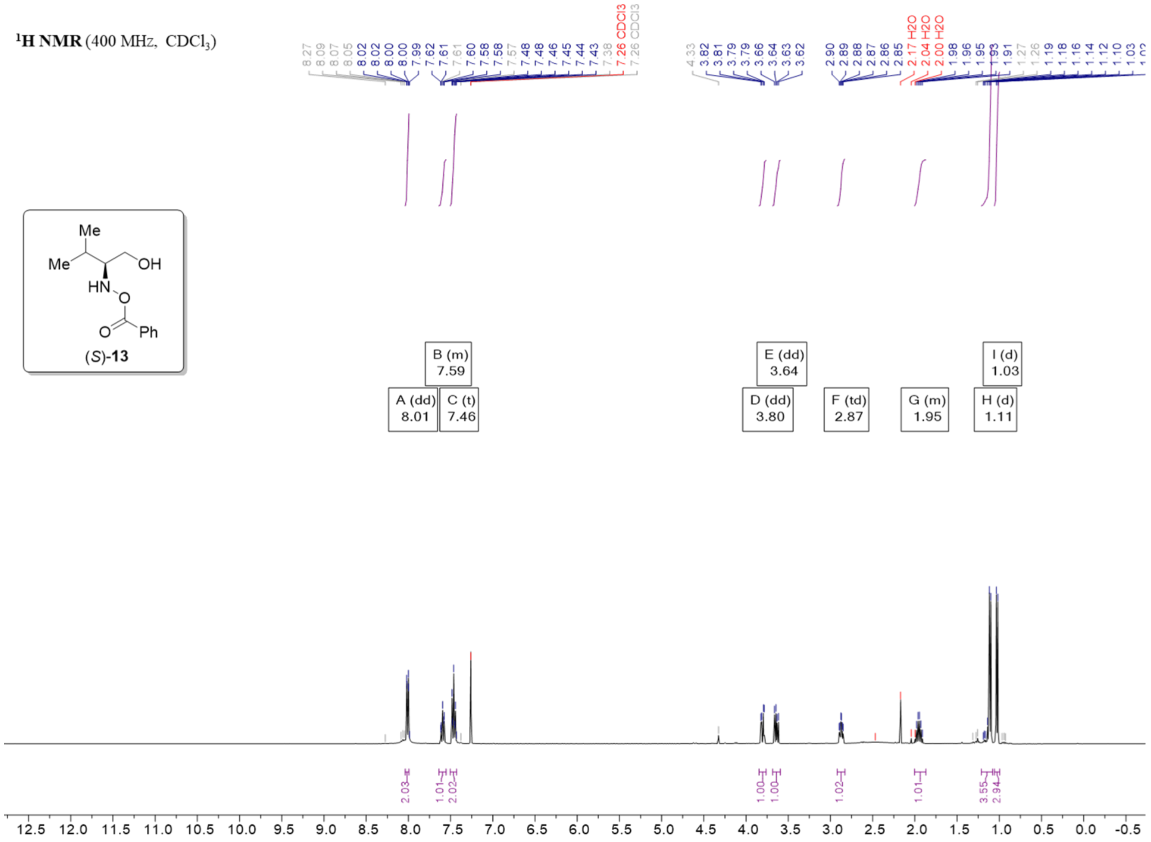

^{13}C NMR (101 MHz, $CDCl_3$)

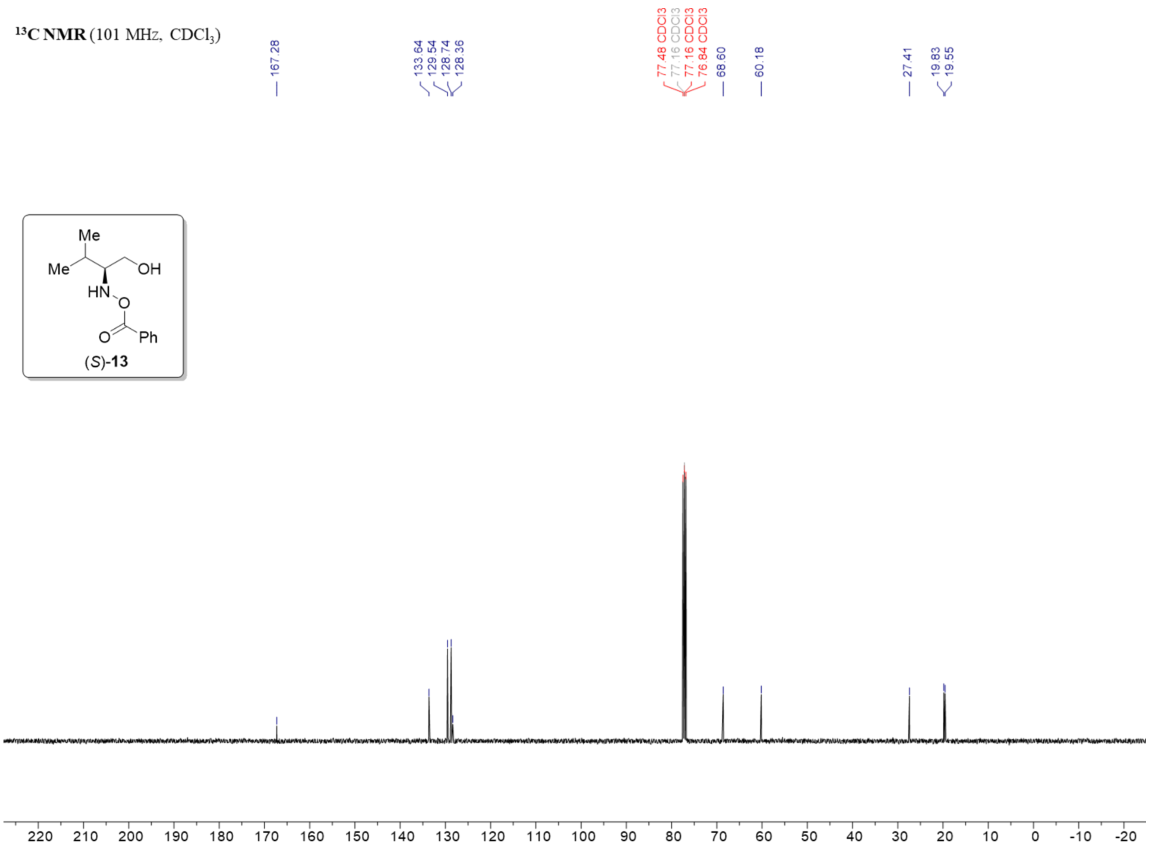

Synthesis of (*S*,*Z*)-1-(1-amino-3-methylbutan-2-yl)-2-hydroxydiazene 1-oxide ((*S*)-4)

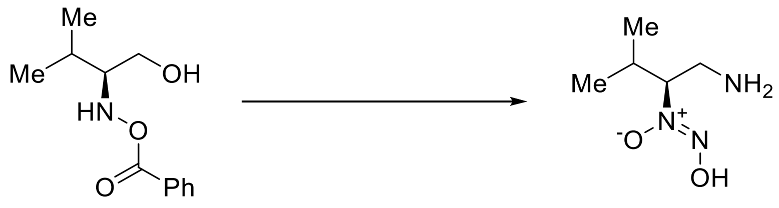

(*S*)-**13** (*S*)-**4**

To a solution of alcohol (*S*)-**13** (791 mg, 3.54 mmol, 1 eq.) in dry THF (20.0 mL) under N_2 at 0 °C, PPh_3 (2.32 g, 8.85 mmol, 2.5 eq.) and *N*-Boc-*tert*-butylcarbamate (1.92 g, 8.85 mmol, 2.5 eq.) were added at 0 °C. The reaction mixture was stirred for 5 minutes before the addition of DEAD (40% in toluene, 3.5 mL, 2.5 eq.) at 0 °C. The reaction was allowed to warm to r.t. and stirred for 18 hours. TLC monitoring (30% EtOAc in hexanes) indicated full consumption of the starting material and the formation of several non-polar UV-active spots. The mixture was concentrated *in vacuo* to give a light-yellow residue (2.50 g). The residue was loaded onto silica gel (250 mL) and eluted with 10% EtOAc in hexanes to give an inseparable mixture containing mono-*N*-Boc and bis-*N*-Boc products (330 mg).

To a solution of the above residue (330 mg) in MeOH (2.40 mL) was added K_2CO_3 (160 mg, 1.16 mmol). The reaction was stirred at r.t. for 2 hours. HPLC-MS showed full deprotection of the benzoyl group. The suspension was filtered, washed with acetone, and the filtrate was concentrated *in vacuo* to give a light yellow solid (524 mg).

The residue was then dissolved in DCM (3.7 mL) at 0 °C and TFA was added (1.60 mL, 21.5 mmol). The reaction was stirred at r.t. for 1 hour. HPLC-MS monitoring indicated full conversion to the desired mass at the solvent front. The mixture was treated with water (5 mL) and separated. The aqueous layer was washed with DCM (2 × 8 mL) and was then concentrated *in vacuo* to give a colourless oil (560 mg). The residue was dissolved in Milli-Q® water (0.5 mL) and filtered through a DSC-18 cartridge (2 g bed) eluting with Milli-Q® water (3 column volumes). The filtrate was concentrated *in vacuo* to give the corresponding β-amino hydroxylamine as a TFA salt (372 mg).

To a solution of the β-amino hydroxylamine TFA salt (372 mg) in 1:1 $EtOH/H_2O$ (3.60 mL) at 0 °C was added an aq. HCl soln. (1 M, 2.30 mL, 2.30 mmol). The mixture was degassed with Ar for 10 minutes. In a separate flask, a solution of $NaNO_2$ (82.0 mg, 1.19 mmol) in water (1.00 mL) was degassed with Ar for 10 minutes before being added to the solution of the hydroxylamine at 0 °C. The mixture was stirred at 0 °C for 30 minutes. HPLC-MS analysis indicated full conversion by detection of the desired product mass eluting at the solvent front. The mixture was concentrated *in vacuo* to give a white gummy residue, which

was treated with acetone (2 × 0.5 mL), sonicated, and filtered to give a light-yellow gum (400 mg). The gum was dissolved in Milli-Q® water (1 mL) and filtered through a DSC-18 cartridge (5 g bed) eluting with Milli-Q® water (3 column volumes). The filtrate was concentrated *in vacuo* to give a colourless gum (294 mg). The gum was purified by preparative HPLC (*Phenomenex Synergi*™ 10 µm Hydro-RP 80 Å, 250 mm × 21.2 mm, 20 mL/min, 100% Milli-Q® water + 0.1% formic acid, 10 minutes, R_t = 6.5 minutes) to give (*S*)-**4** (48.0 mg, 0.326 mmol, 9% over 4 steps) as a white solid.

m.p. = 50.5–54.5 °C

^{1}H NMR (400 MHz, Methanol-d_4) δ = 4.26 – 4.18 (m, 1H), 3.53 (dd, *J* = 13.8, 9.6 Hz, 1H), 3.36 – 3.32 (m, 1H), 2.21 (dp, *J* = 8.6, 6.7 Hz, 1H), 1.06 (d, *J* = 6.7 Hz, 3H), 0.95 (d, *J* = 6.6 Hz, 3H).

^{13}C NMR (126 MHz, Methanol-d_4) δ = 76.00, 40.09, 30.43, 19.24, 19.10.

FTIR $\tilde{\nu}$ (cm^{-1}) = 2971br m, 1672m, 1532m, 1433m, 1302w, 1273m, 1183s, 1134s, 1066m, 976m, 924m, 898m, 837m, 798m, 722m, 699m, 598w, 518m, 443w, 406w.

ESI-HRMS (MeOH): *m/z* 148.10798 ($C_5H_{14}O_2N_3^+$; $[M+H]^+$; calc. 148.10805).

Optical rotation: $[\alpha]_D^{25} = -7.018$ (c = 0.79, methanol).

^{1}H NMR (500 MHz, Methanol-d_4)

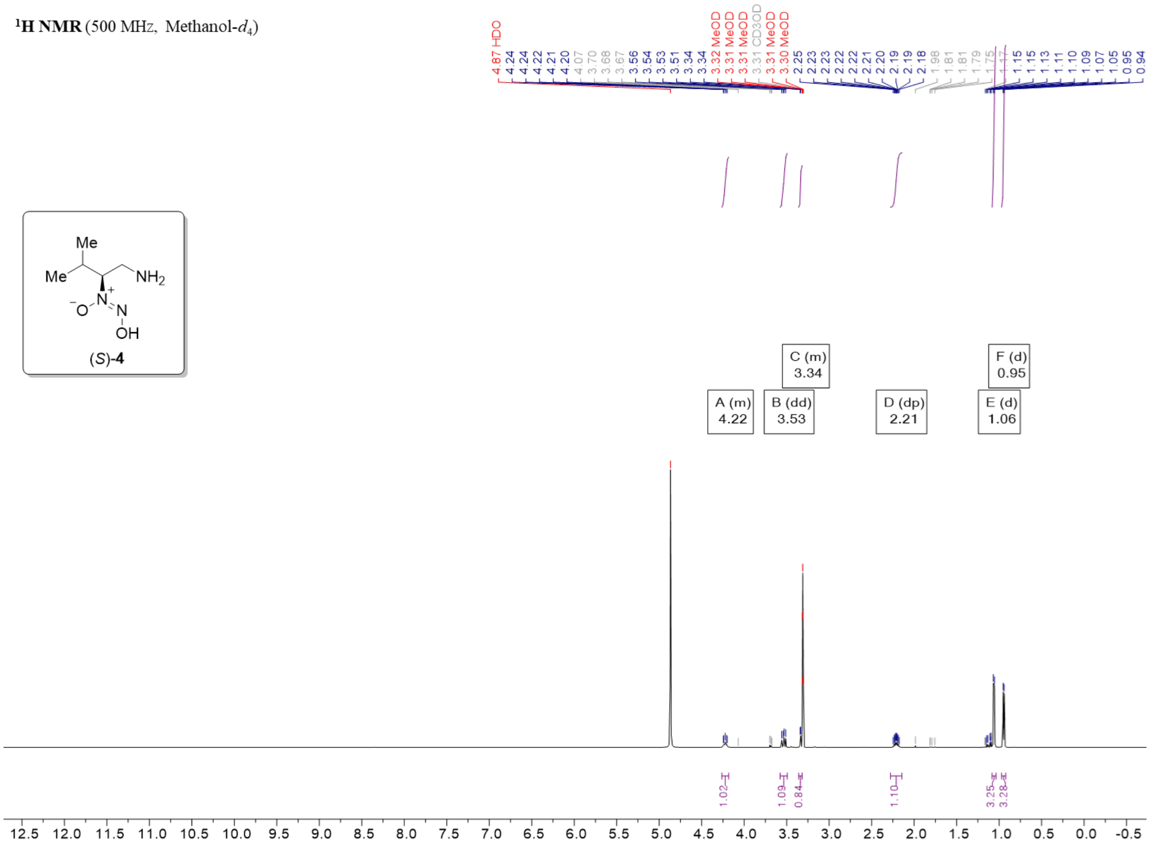

^{13}C NMR (126 MHz, Methanol-d_4)

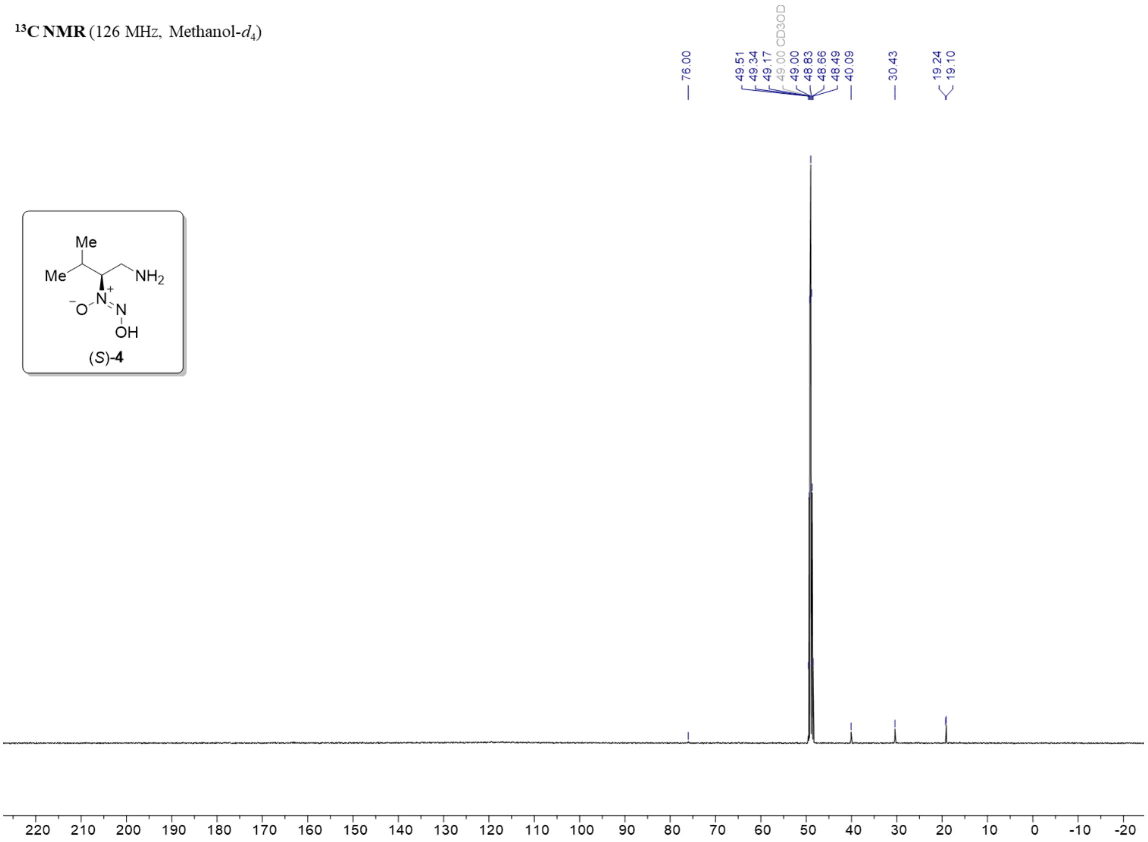

Synthesis (*R*)-2-((benzoyloxy)amino)-3-methylbutan-1-ol ((*R*)-13)

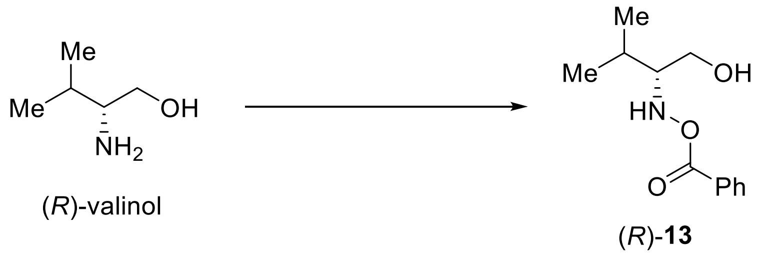

To a suspension of dibenzoylperoxide (1.53 g, 6.32 mmol, 1.3 eq.) and K_2HPO_4 (1.36 g, 7.81 mmol, 1.6 eq.) in dry THF (12.5 mL) at r.t. was added a solution of (*R*)-valinol (500 mg, 4.85 mmol, 1 eq.) in dry THF (5.80 mL) dropwise. The mixture was stirred at r.t. for 16 hours. The mixture was filtered through a short pad of Celite® and eluted with THF. The mixture was concentrated *in vacuo* to give a gummy colourless residue. The residue (1.50 g) was loaded onto silica gel (200 mL) and eluted with 30% EtOAc in hexanes (1 L) to give (*R*)-**13** (824 mg, 3.69 mmol, 76%) as a colourless gum.

R_f = 0.35 (SiO_2, 30% EtOAc in hexanes, UV and $KMnO_4$ stain).

^{1}H NMR (400 MHz, $CDCl_3$) δ = 8.05 – 7.97 (m, 2H), 7.64 – 7.55 (m, 1H), 7.51 – 7.42 (m, 2H), 3.80 (dd, J = 11.7, 3.6 Hz, 1H), 3.64 (dd, J = 11.6, 7.0 Hz, 1H), 2.87 (td, J = 7.3, 3.5 Hz, 1H), 2.04 – 1.87 (m, 1H), 1.11 (d, J = 6.8 Hz, 3H), 1.03 (d, J = 6.9 Hz, 3H).

^{13}C NMR (101 MHz, $CDCl_3$) δ = 167.28, 133.64, 129.54, 128.74, 128.36, 68.60, 60.19, 27.41, 19.83, 19.55.

FTIR $\tilde{\nu}$ (cm^{-1}) = 3243br w, 3065w, 2963m, 2875m, 1720m, 1600m, 1574m, 1451m, 1388m, 1315m, 1270s, 1177m, 1112m, 1069m, 1026m, 975w, 923w, 836w, 781w, 709s, 688m, 542w.

ESI-HRMS (MeOH): *m/z* 224.12810 ($C_{12}H_{18}O_3N^+$; $[M+H]^+$; calc. 224.12812).

Optical rotation: $[\alpha]_D^{25}$ = +11.375 (c = 0.80, $CHCl_3$).

^{1}H NMR (400 MHz, $CDCl_3$)

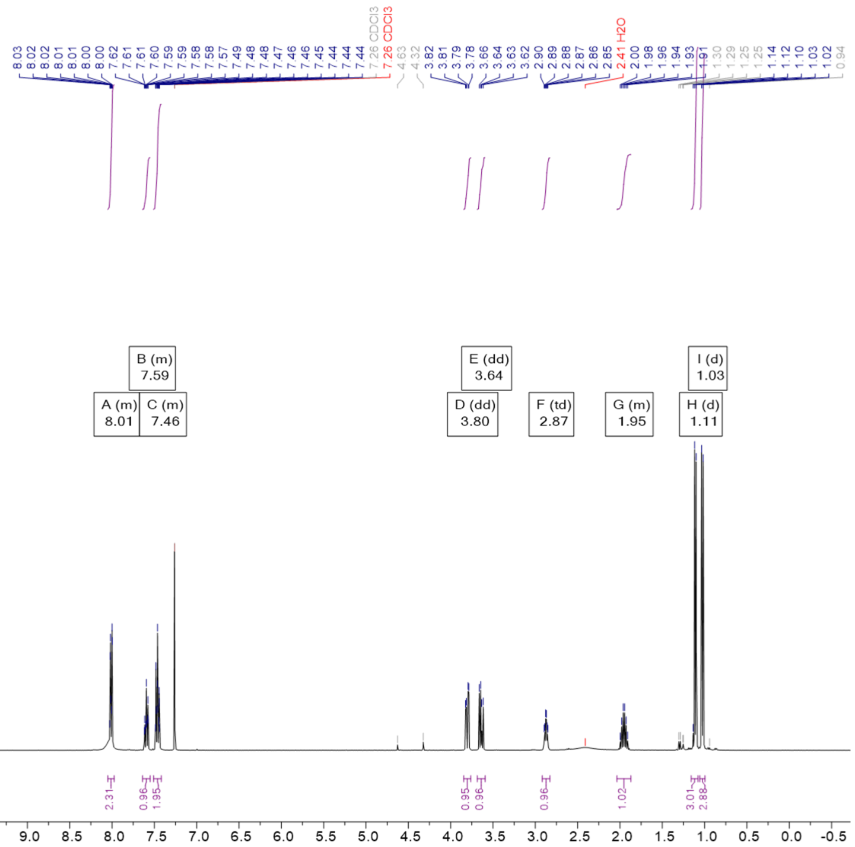

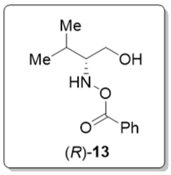

^{13}C NMR (101 MHz, $CDCl_3$)

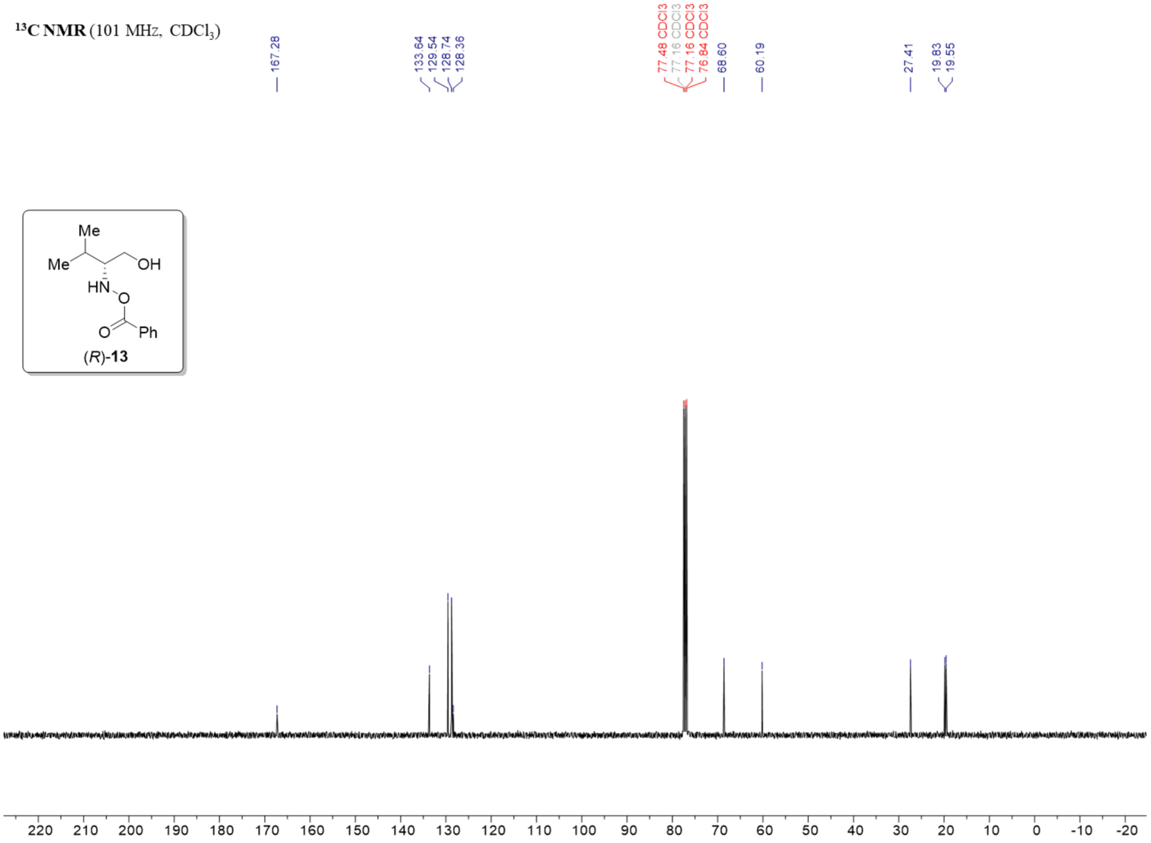

Synthesis of (*R*,*Z*)-1-(1-amino-3-methylbutan-2-yl)-2-hydroxydiazene 1-oxide (((*R*)-4)

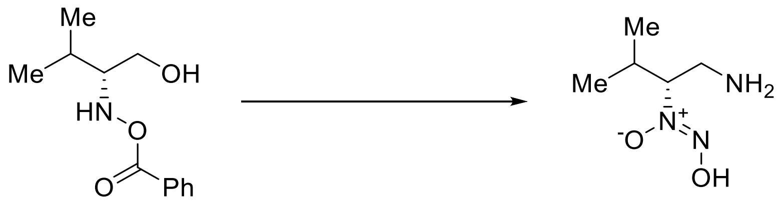

(*R*)-**13** (*R*)-**4**

To a solution of alcohol (*R*)-**13** (750 mg, 3.36 mmol, 1 eq.) in dry THF (19.0 mL) under N_2 at 0 °C, PPh_3 (2.20 g, 8.40 mmol, 2.5 eq.) and *N*-Boc-*tert*-butylcarbamate (1.83 g, 8.40 mmol, 2.5 eq.) were added at 0 °C. The mixture was stirred for 5 minutes before the addition of DEAD (40% in toluene, 3.3 mL, 2.5 eq.) at 0 °C. The reaction mixture was then warmed to r.t. and stirred for 18 hours. TLC monitoring (30% EtOAc in hexanes) indicated full consumption of the starting material and the formation of several non-polar UV-active spots. The reaction mixture was concentrated *in vacuo* to give a light-yellow residue (2.10 g). The residue was loaded onto silica gel (200 mL) and eluted with 10% EtOAc in hexanes to give an inseparable mixture containing mono-*N*-Boc and bis-*N*-Boc products (376 mg).

To a solution of the above mixture (376 mg) in MeOH (2.4 mL) was added K_2CO_3 (182 mg, 1.32 mmol). The reaction was stirred at r.t. for 2 hours. HPLC-MS monitoring indicated full deprotection of the benzoyl group. The suspension was filtered, washed with acetone, and the filtrate was concentrated *in vacuo* to give a light yellow solid (507 mg).

This residue was taken up in DCM (3.70 mL) at 0 °C and TFA was added (1.80 mL, 24.2 mmol). The reaction mixture was stirred at r.t. for 1 hour. HPLC-MS monitoring indicated full conversion, as evident from the detection of the desired mass at the solvent front. The mixture was treated with water (5 mL) and separated. The aqueous layer was washed with DCM (2 × 8 mL) and was then concentrated *in vacuo* to give a colourless oil (540 mg). The oil was reconstituted in Milli-Q® water (0.5 mL) and filtered through a DSC-18 cartridge (2 g bed) eluting with Milli-Q® water (3 column volumes). The filtrate was concentrated *in vacuo* to give the corresponding β-amino hydroxylamine as a TFA salt (438 mg).

To a solution of the β-amino hydroxylamine TFA salt above (438 mg) in 1:1 EtOH/H_2O (4.20 mL) at 0 °C was added an aq. HCl soln. (1M, 2.70 mL, 2.70 mmol). The reaction mixture was degassed with Ar for 10 minutes. In a separate flask, a solution of $NaNO_2$ (97.0 mg, 1.40 mmol) in water (1.00 mL) was degassed with Ar for 10 minutes before being added to the solution of the hydroxylamine at 0 °C. The reaction mixture was stirred at 0 °C for 30 minutes. HPLC-MS monitoring indicated full conversion, as evident from the desired product mass eluting at the solvent front. The mixture was concentrated *in vacuo* to give a white

gummy residue, which was treated with acetone (2 × 0.5 mL), sonicated, and filtered to give a light-yellow gum (404 mg). The gum was reconstituted in Milli-Q® water (1 mL) and filtered through a DSC-18 cartridge (5 g bed) eluting with Milli-Q® water (3 column volumes). The filtrate was concentrated *in vacuo* to give a colourless gum (290 mg). The gum was purified by preparative HPLC (*Phenomenex Synergi™* 10 µm Hydro-RP 80 Å, 250 mm × 21.2 mm, 20 mL/min, 100% Milli-Q® water + 0.1% formic acid, 10 minutes, R_t = 6.5 minutes) to give (*R*)-**4** (54.0 mg, 0.367 mmol, 11% over 4 steps) as a white solid.

m.p. = 51.2–55.7 °C

^{1}H NMR (400 MHz, Methanol-d_4) δ = 4.26 – 4.18 (m, 1H), 3.53 (dd, J = 13.8, 9.6 Hz, 1H), 3.35 – 3.32 (m, 1H), 2.21 (dp, J = 8.5, 6.6 Hz, 1H), 1.06 (d, J = 6.8 Hz, 3H), 0.95 (d, J = 6.6 Hz, 3H).

^{13}C NMR (126 MHz, Methanol-d_4) δ = 75.98, 40.10, 30.42, 19.24, 19.10.

FTIR $\tilde{\nu}$ (cm^{-1}) = 2971br m, 1672m, 1532m, 1432m, 1396m, 1302w, 1272m, 1181s, 1133s, 1066m, 976m, 924m, 897m, 836m, 798m, 722m, 699m, 598w, 517m, 443w, 411w.

ESI-HRMS (MeOH): *m/z* 148.10810 ($C_5H_{14}O_2N_3^+$; $[M+H]^+$; calc. 148.10805).

Optical rotation: $[\alpha]_D^{25}$ = +8.085 (c 0.80, MeOH).

^{1}H NMR (500 MHz, Methanol-d_4)

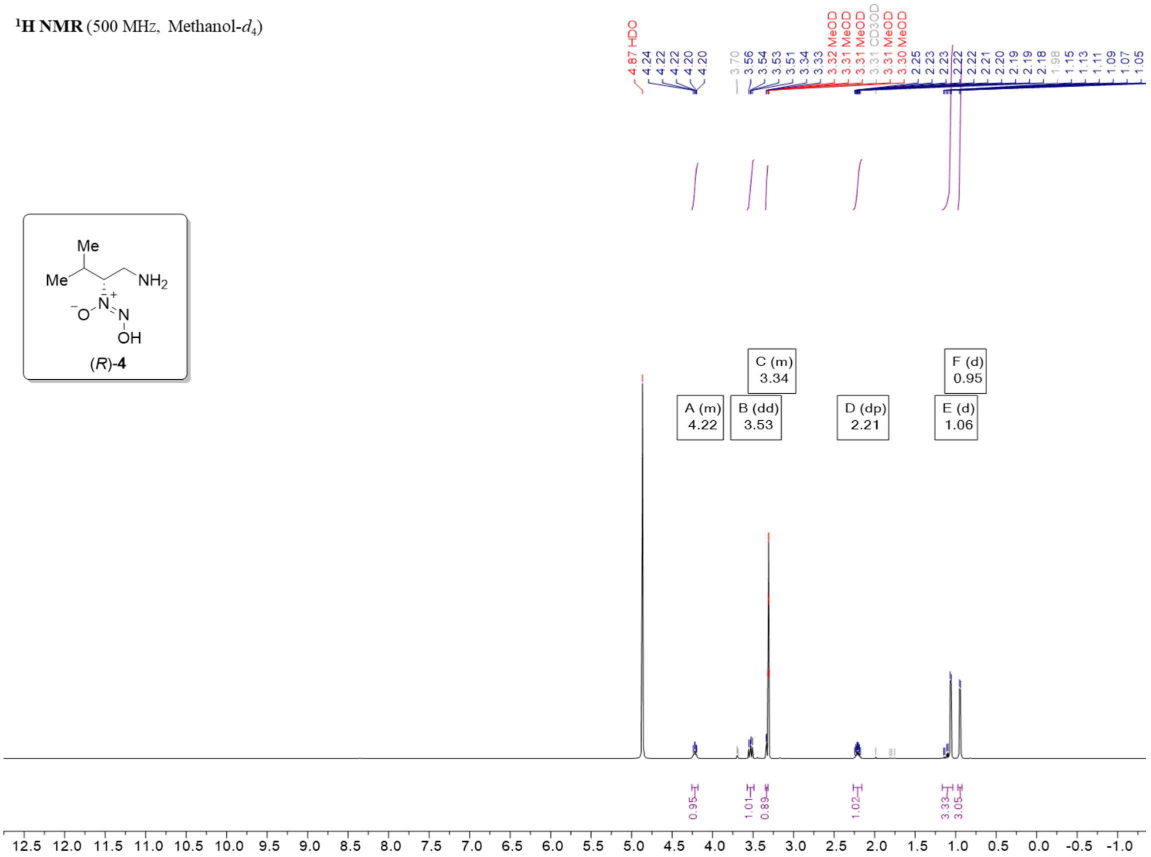

^{13}C NMR (126 MHz, Methanol-d_4)

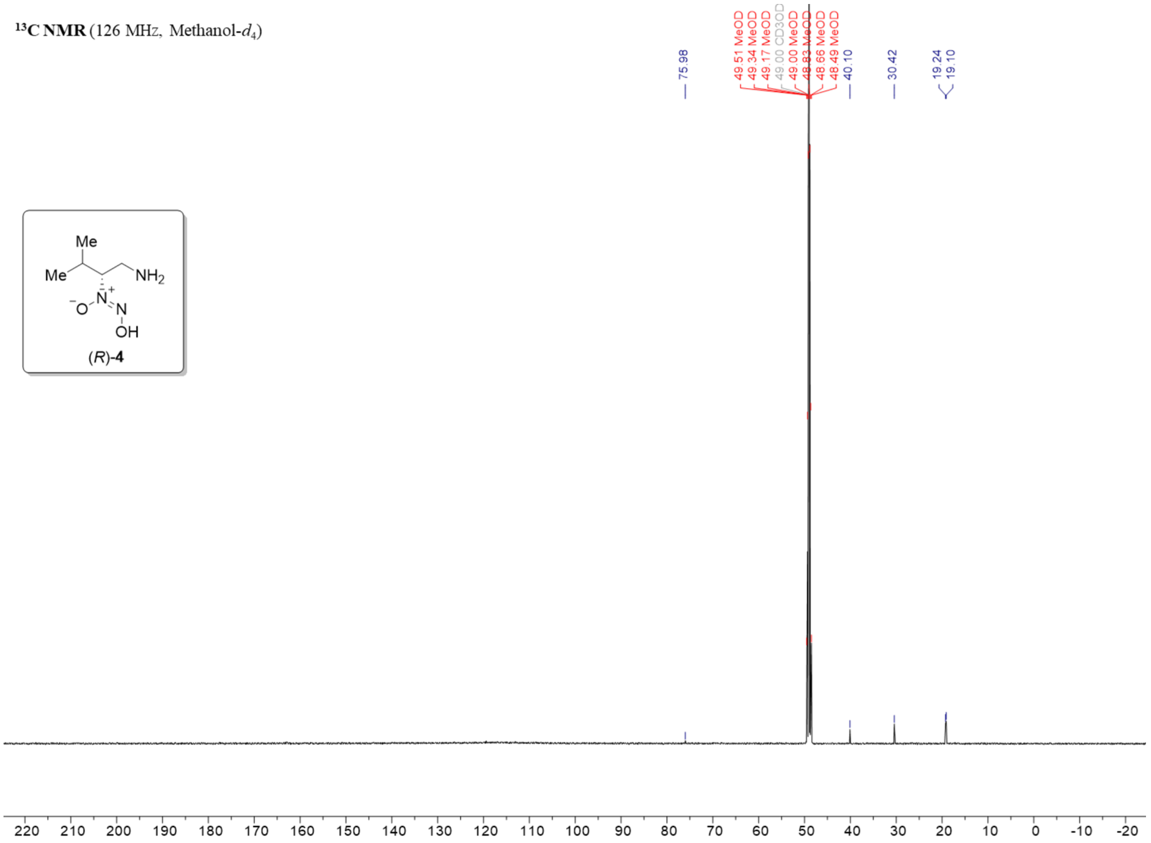

Synthetic attempts towards enantiomerically pure aldehyde (2)

Synthesis of methyl hydroxy-L-valinate (15)

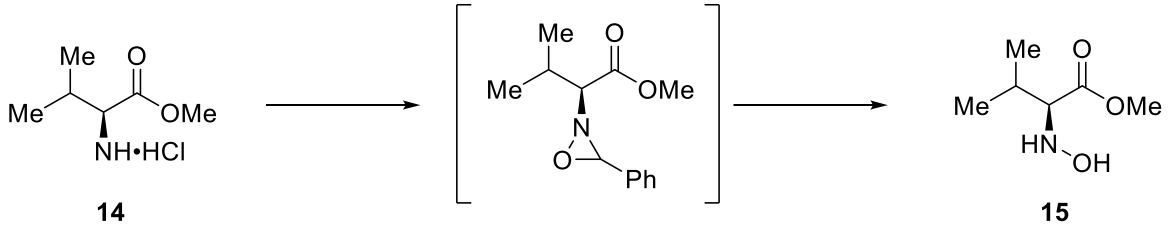

This experiment was conducted using a modified published procedure.[15] To a solution of L-valine methyl ester hydrochloride **14** (6.25 g, 37.3 mmol, 1 eq.) in dry methanol (120 mL), dry Na_2CO_3 (7.00 g, 55.9 mmol, 1.5 eq.) and *p*-anisaldehyde (4.60 mL, 37.0 mmol, 1 eq.) were added, and the reaction mixture was stirred for 18 hours at r.t. followed by 1 hour at 40 °C. The reaction mixture was filtered through a short pad of Celite® and concentrated *in vacuo*. The residue was redissolved in diethyl ether, filtered, and concentrated *in vacuo* to give a slightly yellow crude product, which was used without further purification in the next step. The imine was dissolved in dry DCM (30 mL) and cooled to −5 °C in an ice/NaCl bath. A solution of *m*-CPBA (8.02 g, 37.3 mmol, 1 eq.) in DCM was dried over $MgSO_4$, filtered and added dropwise to the reaction mixture over 1 hour. The reaction was allowed to warm to r.t. and was stirred for 24 hours. The reaction mixture was filtered, washed with a sat. aq. solution of $NaHCO_3$ (3 × 80 mL) and brine (80 mL), dried over anhydrous Na_2SO_4, filtered, and concentrated *in vacuo* to afford the oxaziridine, which was used without further purification in the next step. The oxaziridine was dissolved in dry MeOH (80 mL) and treated with hydroxylamine hydrochloride (5.23 g, 74.6 mmol, 2 eq.) at r.t. for 24 hours. The solvent was removed under reduced pressure, and the residues were suspended in H_2O and washed with Et_2O (3 × 80 mL). The aqueous layer was neutralized with $NaHCO_3$ and extracted with Et_2O (3 × 80 mL). The combined organic layers were dried over Na_2SO_4, filtered, and concentrated *in vacuo* to obtain the hydroxylamine **15** as a slightly yellow solid (3.16 g, 21.5 mmol, 58% over 3 steps).

$\mathbf{R_f}$ = 0.22 (SiO_2, 50% EtOAc in pentane, $KMnO_4$ stain).

m.p. = 157–161 °C

^{1}**H NMR** (400 MHz, Methanol-*d₄*): δ = 3.74 (s, 3H), 1.93 – 1.78 (m, J = 6.9 Hz, 1H), 0.99 (d, J = 6.9 Hz, 3H), 0.92 (d, J = 6.8 Hz, 3H).

^{13}**C NMR** (101 MHz, Methanol-d_4): δ = 175.82, 73.08, 52.00, 30.09, 19.94, 19.49.

FTIR $\tilde{\nu}$ (cm^{-1}) = 3259m, 3181br m, 2953m, 1742s, 1430m, 1370m, 1273m, 1185m, 1156s, 1030m, 992m, 764m, 683m, 524m, 533m.

Optical rotation: $[\alpha]_D^{25}$ = +9.200 (c 0.80, CH_2Cl_2).

ESI-HRMS (MeOH): *m/z* 148.09662 ($C_6H_{14}NO_3^+$; $[M+H]^+$; calc. 148.09682).

^{1}H NMR (400 MHz, Methanol-d_4)

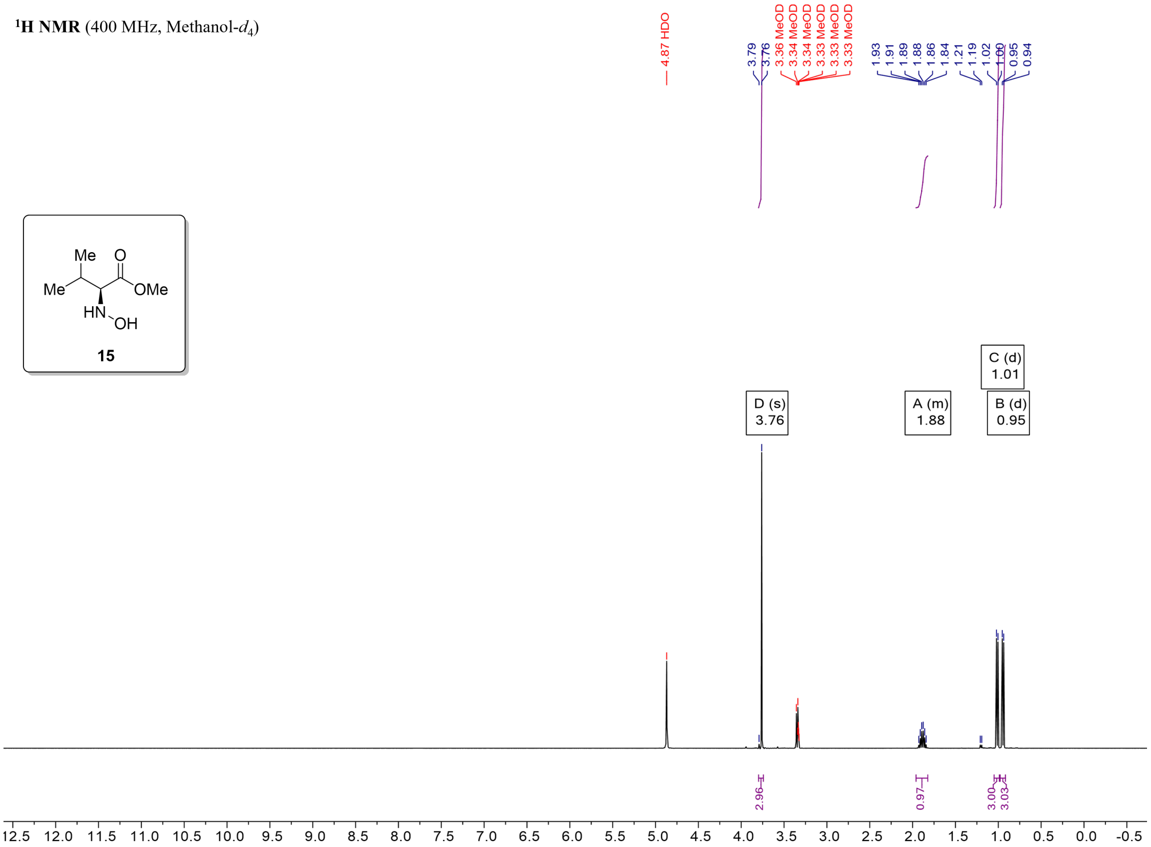

^{13}C NMR (101 MHz, Methanol-d_4)

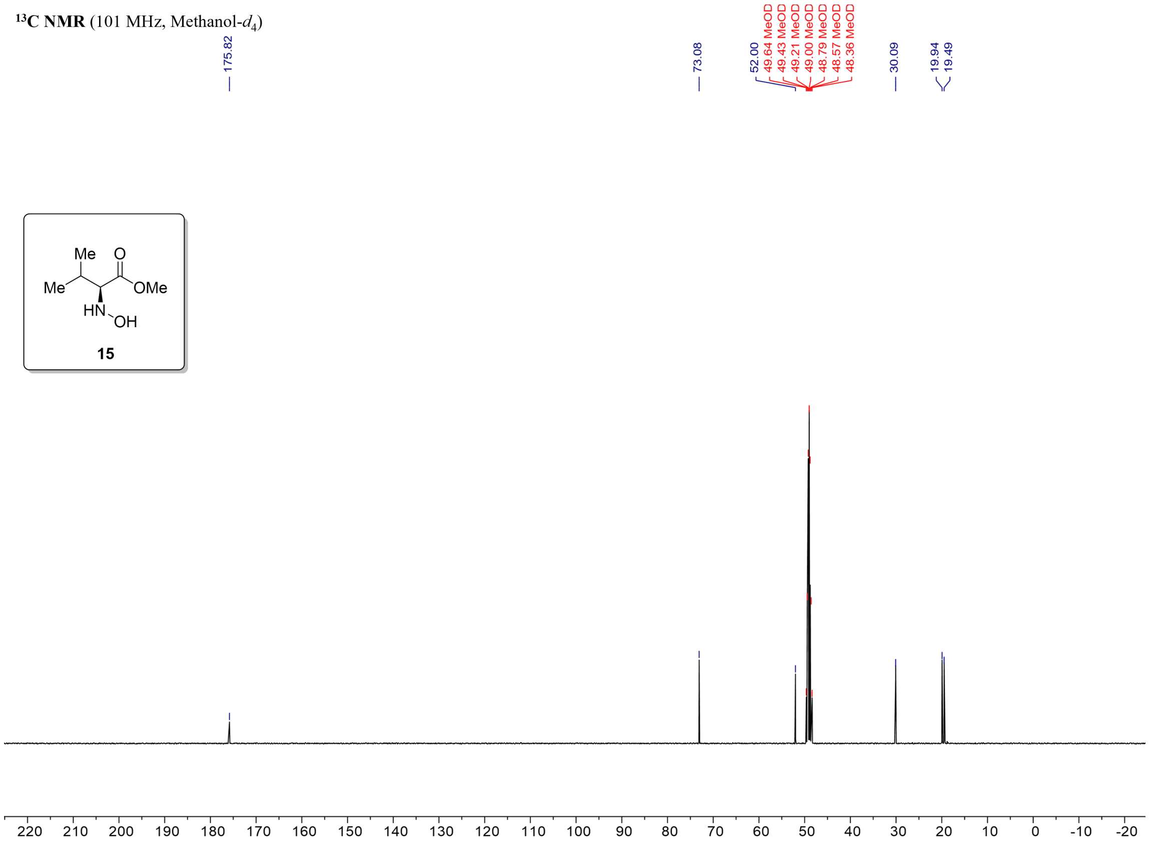

Synthesis of (*Z*)-2-(allyloxy)-1-(3-methyl-1-oxobutan-2-yl)diazene 1-oxide (16) and (*Z*)-2-(allyloxy)-1-(1,1-dihydroxy-3-methylbutan-2-yl)diazene 1-oxide (17)

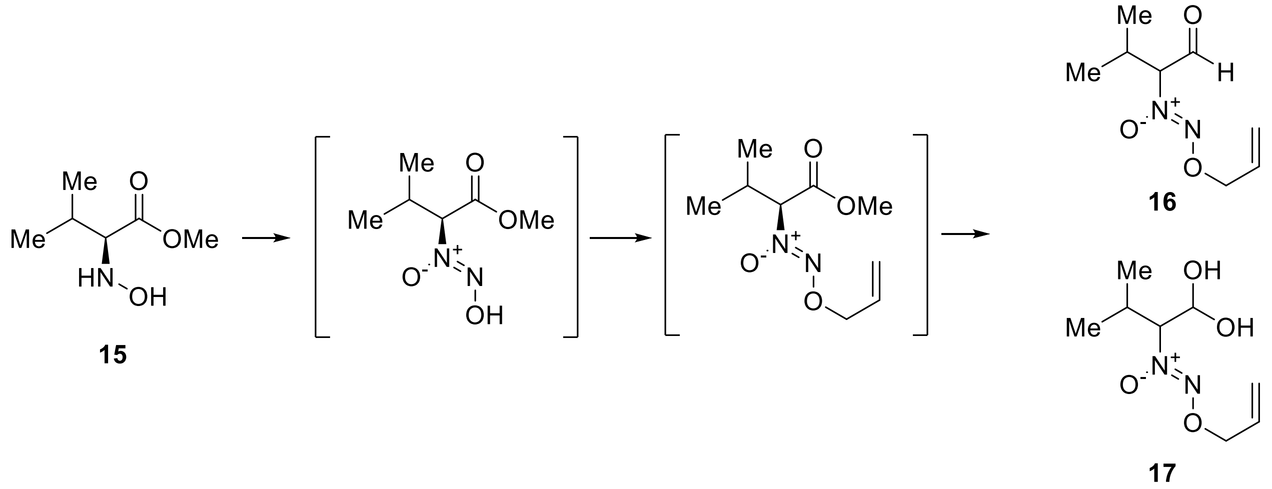

This experiment was conducted using a modified published procedure.[16,17] To a solution of (*S*)-methyl *N*-hydroxy valinate **15** (500 mg, 3.40 mmol, 1 eq.) in 1:1 EtOH/H_2O (15.0 mL) was added an aq. HCl soln. (4 M, 850 µL, 3.40 mmol, 1 eq.) at 0 °C. The mixture was degassed with Ar for 10 minutes. In a separate flask, a solution of $NaNO_2$ (235 mg, 3.40 mmol) in water (3.00 mL) was degassed with Ar for 10 minutes before being added dropwise with a syringe pump over 45 minutes to the solution of hydroxylamine at 0 °C. The mixture was stirred at 0 °C for 30 minutes. The yellow solution was diluted with H_2O (30 mL) and extracted with DCM (3 × 30 mL). The combined organic layers were washed with brine (30 mL), dried over anhydrous Na_2SO_4, filtered, and concentrated *in vacuo*. Full removal of the solvent was avoided, due to the observed spontaneous decomposition. The green oil was used in the next step without further purification. The residue was dissolved in dry DMF (5.00 mL) and Na_2CO_3 (324 mg, 3.40 mmol, 1 eq.) was added. Allyl bromide (186 µL, 2.15 mmol) was added at 0 °C and the reaction was allowed to warm to r.t. and stirred for 12 hours. The reaction mixture was filtered through a short pad of Celite® and concentrated *in vacuo* to obtain a yellow oil (235 mg) as a mixture of two compounds diverging slightly in the H and C NMR signals for the isopropyl group, but with similar R_f values on TLC. These compounds could not be further separated and were directly used as the starting material in the next step.

To a solution of the protected diazeniumdiolates (95.0 mg.) in dry DCM (12.0 mL) at –78 °C was added DIBAL-H in hexanes (1 M, 1.20 mL, 1.20 mmol, 2.7 eq.). The reaction was stirred at –78 °C for 1 hour and quenched with EtOAc (3 mL) followed by the addition of 1 M aq. NaOH (3 mL). The reaction was allowed to warm to r.t., treated with H_2O (20 mL), and extracted with DCM (3 × 20 mL). The combined organic layers were dried over anhydrous $MgSO_4$, filtered, and concentrated *in vacuo.* The residue was

purified by column chromatography with 20% – 50% diethyl ether in pentane to obtain a complex mixture of aldehydes **16** and aldehyde hydrate **17** as a yellow oil (83 mg). The aldehyde was assigned by the characteristic peak at 9.64 ppm in the ^{1}H NMR spectrum and at 195.3 ppm in the ^{13}C NMR spectrum. The presence of the diol was assigned by the presence of two doublets at 6.45 and 6.29 ppm corresponding for the two OH groups and with the downfield shielded carbon at 88.0 ppm in the ^{13}C NMR spectrum. The identity of the diol was further corroborated by an X-ray analysis of a single crystal obtained by treating the compound with a pentane/diethyl ether mixture at –20 °C. Due to the complexity of those mixtures, another route was designed for the project.

Characterization of the mixture of 16 and 17

R_f = 0.2 (SiO_2, 50% diethyl ether in pentane, $KMnO_4$ stain).

^{1}H NMR (500 MHz, DMSO-*d_6*) δ 9.64 (d, J = 0.9 Hz, 1H), 6.29 (d, J = 7.1 Hz, 1H), 6.20 (d, J = 6.4 Hz, 1H), 6.01 – 5.88 (m, 2H), 5.40 – 5.22 (m, 4H), 5.11 (q, J = 6.9 Hz, 1H), 4.85 (dd, J = 8.0, 0.9 Hz, 1H), 4.77 (d, J = 5.7 Hz, 2H), 4.68 (d, J = 5.5 Hz, 1H), 3.79 (dd, J = 7.5, 5.8 Hz, 1H), 2.49 – 2.42 (m, 1H), 2.18 (pd, J = 6.9, 5.8 Hz, 1H), 1.05 (d, J = 6.8 Hz, 3H), 0.95 – 0.89 (m, 9H).

^{13}C NMR (126 MHz, DMSO-*d_6*) δ 195.3, 133.0, 132.7, 119.1, 118.5, 88.0, 83.8, 81.8, 73.9, 73.2, 27.7, 27.6, 19.5, 18.4, 18.3, 17.6.

Optical rotation: $[\alpha]_D^{25}$ = +1.5 (c 0.84, CH_2Cl_2).

ESI-HRMS (MeOH): aldehydes (**16**): *m/z* 209.08965 ($C_8H_{14}N_2NaO_3^+$; $[M\text{+Na}]^+$; calc. 209.08966) and diols (**17**): *m/z* 227.10016 ($C_8H_{16}N_2NaO_4^+$; $[M\text{+Na}]^+$; calc. 227.10023)

X-ray crystallography: Structure obtained after analysis of a single crystal of the diol (see **Supplementary Table 6** and **Supplementary Data 3**)

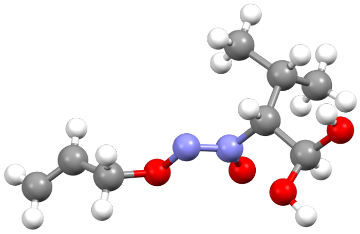

¹H NMR (500 MHz, DMSO-d_6)

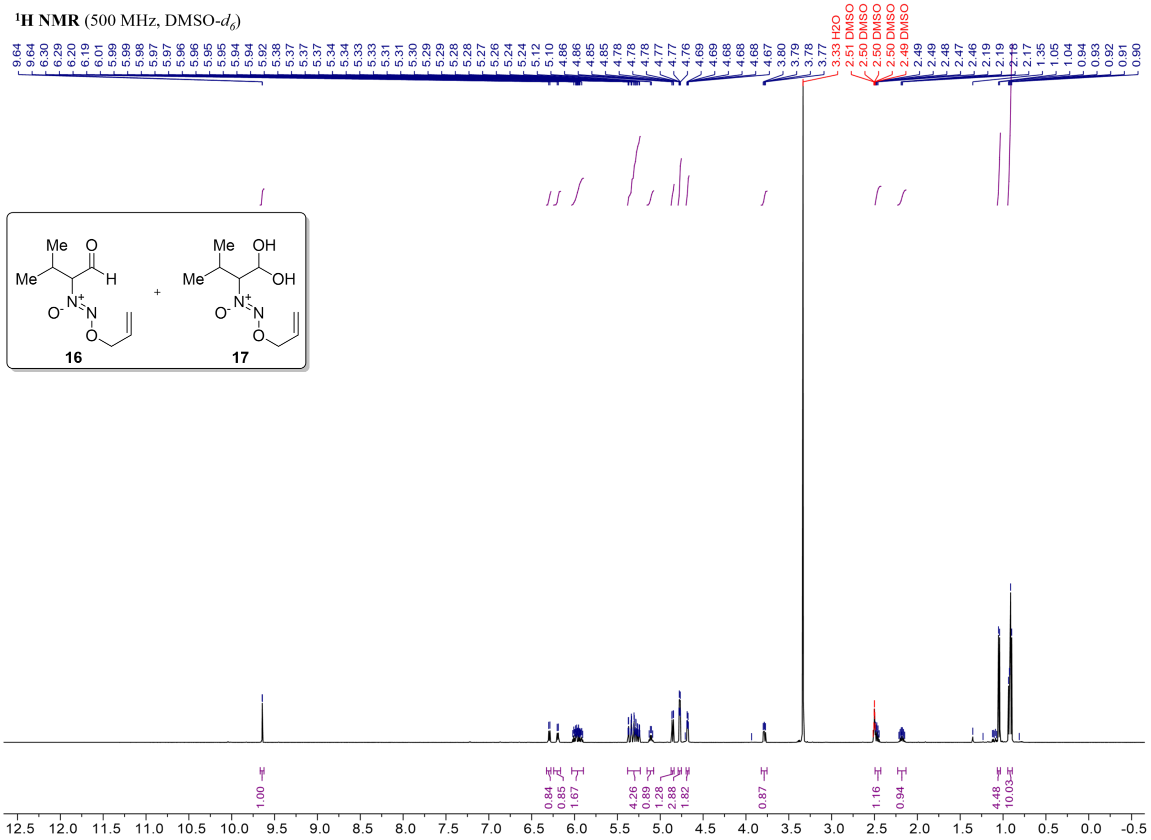

¹³C NMR (126 MHz, DMSO-d_6)

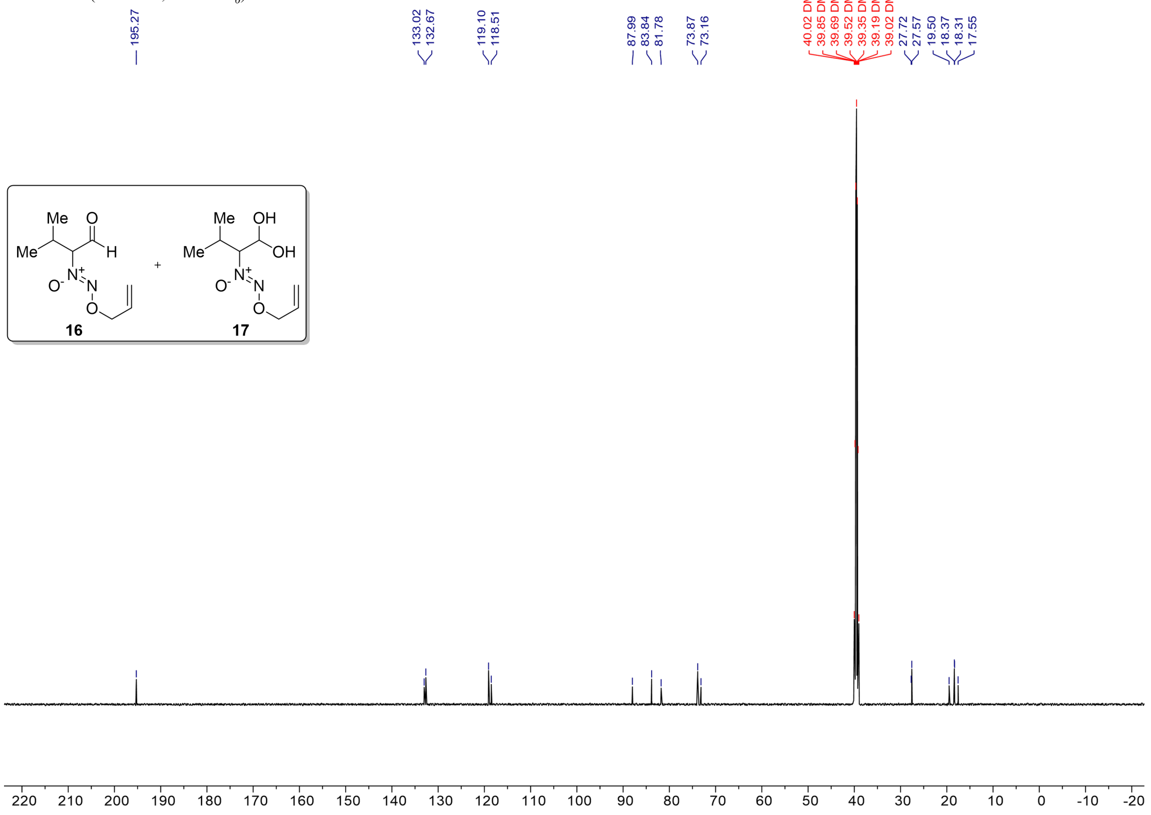

Supplementary References

1. Jenul, C. *et al.* Biosynthesis of fragin is controlled by a novel quorum sensing signal. *Nat. Commun.* **9**, 1297 (2018).

2. Dolomanov, O. V., Bourhis, L. J., Gildea, R. J., Howard, J. A. K. & Puschmann, H. OLEX2: a complete structure solution, refinement and analysis program. *J. Appl. Crystallogr.* **42**, 339–341 (2009).

3. Sheldrick, G. M. SHELXT – Integrated space-group and crystal-structure determination. *Acta Crystallogr. Sect. A: Found. Adv.* **71**, 3–8 (2015).

4. Sheldrick, G. M. Crystal structure refinement with SHELXL. *Acta Crystallogr. Sect. C: Struct. Chem.* **71**, 3–8 (2015).

5. Spek, A. L. Structure validation in chemical crystallography. *Acta Crystallogr. Sect. D: Biol. Crystallogr.* **65**, 148–155 (2009).

6. Sieber, S. *et al.* Biosynthesis and Structure–Activity Relationship Investigations of the Diazeniumdiolate Antifungal Agent Fragin. *ChemBioChem* **21**, 1587–1592 (2020).

7. Gotschlich, A. *et al.* Synthesis of Multiple *N*-Acylhomoserine Lactones is Wide-spread Among the Members of the *Burkholderia cepacia* Complex. *Syst. Appl. Microbiol.* **24**, 1–14 (2001).

8. Herrero, M., Lorenzo, V. de & Timmis, K. N. Transposon vectors containing non-antibiotic resistance selection markers for cloning and stable chromosomal insertion of foreign genes in gram-negative bacteria. *J. Bacteriol.* **172**, 6557–6567 (1990).

9. O'Grady, E. P., Viteri, D. F., Malott, R. J. & Sokol, P. A. Reciprocal regulation by the CepIR and CciIR quorum sensing systems in *Burkholderia cenocepacia*. *BMC Genomics* **10**, 441 (2009).

10. Habjanič, J., Mathew, A., Eberl, L. & Freisinger, E. Deciphering the Enigmatic Function of *Pseudomonas* Metallothioneins. *Front. Microbiol.* **11**, 1709 (2020).

11. Flannagan, R. S., Linn, T. & Valvano, M. A. A system for the construction of targeted unmarked gene deletions in the genus *Burkholderia*. *Environ. Microbiol.* **10**, 1652–1660 (2008).

12. Tiecco, M., Testaferri, L., Tingoli, M. & Bartoli, D. Selenium-catalyzed conversion of methyl ketones into α-keto acetals. *J. Org. Chem.* **55**, 4523–4528 (1990).

13. Allerton, C. M. N., Blagg, J., Bunnage, M. E. & Steele, J. Preparation of substituted imidazoles as TAFIa inhibitors. *WO 2002014285. 2002*; Chem Abstr 2002; 136: 184120, page 142.

14. Chang, Z. Y. & Coates, R. M. Diastereoselectivity of organometallic additions to nitrones bearing stereogenic *N*-substituents. *J. Org. Chem.* **55**, 3464–3474 (1990).

15. Breuning, M., Häuser, T. & Tanzer, E.-M. A Novel One-Pot Procedure for the Stereoselective Synthesis of α-Hydroxy Esters from Ortho Esters. *Org. Lett.* **11**, 4032–4035 (2009).

16. Cason, J. & Prout, F. S. The Synthesis of tris-(Hydroxymethyl)-methylnitramine and Other Compounds Related to tris-(Hydroxymethyl)-aminomethane. *J. Am. Chem. Soc.* **71**, 1218–1221 (1949).

17. Holland, R. J. *et al.* Direct Reaction of Amides with Nitric Oxide To Form Diazeniumdiolates. *J. Org. Chem.* **79**, 9389–9393 (2014).
