# Supplementary material for: Functional biosynthetic stereodivergence in a gene cluster via a dihydrosydnone N-oxide
Source: Commun Chem. 2024 Dec 19;7:301. doi: 10.1038/s42004-024-01372-3 (PMC11659414; doi:10.1038/s42004-024-01372-3)

checkCIF/PLATON report

Structure factors have been supplied for datablock(s) 17

THIS REPORT IS FOR GUIDANCE ONLY. IF USED AS PART OF A REVIEW PROCEDURE FOR PUBLICATION, IT SHOULD NOT REPLACE THE EXPERTISE OF AN EXPERIENCED CRYSTALLOGRAPHIC REFEREE.

No syntax errors found.   CIF dictionary   Interpreting this report

Datablock: 17

Bond precision:	C-C = 0.0016 A		Wavelength=1.54184
Cell:	a=9.7461(2)	b=10.1007(4)	c=10.8278(2)
	alpha=90	beta=90.358(2)	gamma=90
Temperature:	160 K		

	Calculated	Reported
Volume	1065.89(5)	1065.89(5)
Space group	P 21/c	P 21/c
Hall group	-P 2ybc	-P 2ybc
Moiety formula	C8 H16 N2 O4	C8 H16 N2 O4
Sum formula	C8 H16 N2 O4	C8 H16 N2 O4
Mr	204.23	204.23
Dx,g cm-3	1.273	1.273
Z	4	4
Mu (mm-1)	0.860	0.860
F000	440.0	440.0
F000'	441.53	
h,k,lmax	12,12,13	12,12,13
Nref	2130	2108
Tmin,Tmax	0.807,0.983	0.877,1.000
Tmin'	0.807	

Correction method= # Reported T Limits: Tmin=0.877 Tmax=1.000
AbsCorr = MULTI-SCAN

Data completeness= 0.990   Theta(max)= 72.998

R(reflections)= 0.0316(1971)   wR2(reflections)= 0.0843(2108)

S = 1.043   Npar= 138

The following ALERTS were generated. Each ALERT has the format
test-name_ALERT_alert-type_alert-level.
Click on the hyperlinks for more details of the test.

Alert level C

```
PLAT911_ALERT_3_C Missing FCF Refl Between Thmin & STh/L=    0.600          3 Report
                0 12  0,  -1 12  1,   0 12  1,
```

Alert level G

```
PLAT395_ALERT_2_G Deviating  X-O-Y   Angle From 120 for O4       .      109.6 Degree
PLAT432_ALERT_2_G Short Inter X...Y Contact  O2       ..C6       .       3.02 Ang.
                                         -x,1-y,1-z  =      3_566 Check
PLAT899_ALERT_4_G SHELXL2018 is Deprecated and Succeeded by SHELXL     2019/3 Note
PLAT912_ALERT_4_G Missing # of FCF Reflections Above STh/L=  0.600         19 Note
PLAT969_ALERT_5_G The 'Henn et al.' R-Factor-gap value ..........       6.72 Note
              Predicted wR2: Based on SigI**2  1.26 or SHELX Weight  8.36
PLAT978_ALERT_2_G Number C-C Bonds with Positive Residual Density.          5 Info
```

0 **ALERT level A** = Most likely a serious problem - resolve or explain
0 **ALERT level B** = A potentially serious problem, consider carefully
1 **ALERT level C** = Check. Ensure it is not caused by an omission or oversight
6 **ALERT level G** = General information/check it is not something unexpected

0 ALERT type 1 CIF construction/syntax error, inconsistent or missing data
3 ALERT type 2 Indicator that the structure model may be wrong or deficient
1 ALERT type 3 Indicator that the structure quality may be low
2 ALERT type 4 Improvement, methodology, query or suggestion
1 ALERT type 5 Informative message, check

It is advisable to attempt to resolve as many as possible of the alerts in all categories. Often the minor alerts point to easily fixed oversights, errors and omissions in your CIF or refinement strategy, so attention to these fine details can be worthwhile. In order to resolve some of the more serious problems it may be necessary to carry out additional measurements or structure refinements. However, the purpose of your study may justify the reported deviations and the more serious of these should normally be commented upon in the discussion or experimental section of a paper or in the "special_details" fields of the CIF. checkCIF was carefully designed to identify outliers and unusual parameters, but every test has its limitations and alerts that are not important in a particular case may appear. Conversely, the absence of alerts does not guarantee there are no aspects of the results needing attention. It is up to the individual to critically assess their own results and, if necessary, seek expert advice.

Publication of your CIF in IUCr journals

A basic structural check has been run on your CIF. These basic checks will be run on all CIFs submitted for publication in IUCr journals (*Acta Crystallographica*, *Journal of Applied Crystallography*, *Journal of Synchrotron Radiation*); however, if you intend to submit to *Acta Crystallographica Section C* or *E* or *IUCrData*, you should make sure that full publication checks are run on the final version of your CIF prior to submission.

Publication of your CIF in other journals

Please refer to the *Notes for Authors* of the relevant journal for any special instructions relating to CIF submission.

PLATON version of 06/01/2024; check.def file version of 05/01/2024

Datablock 17 - ellipsoid plot

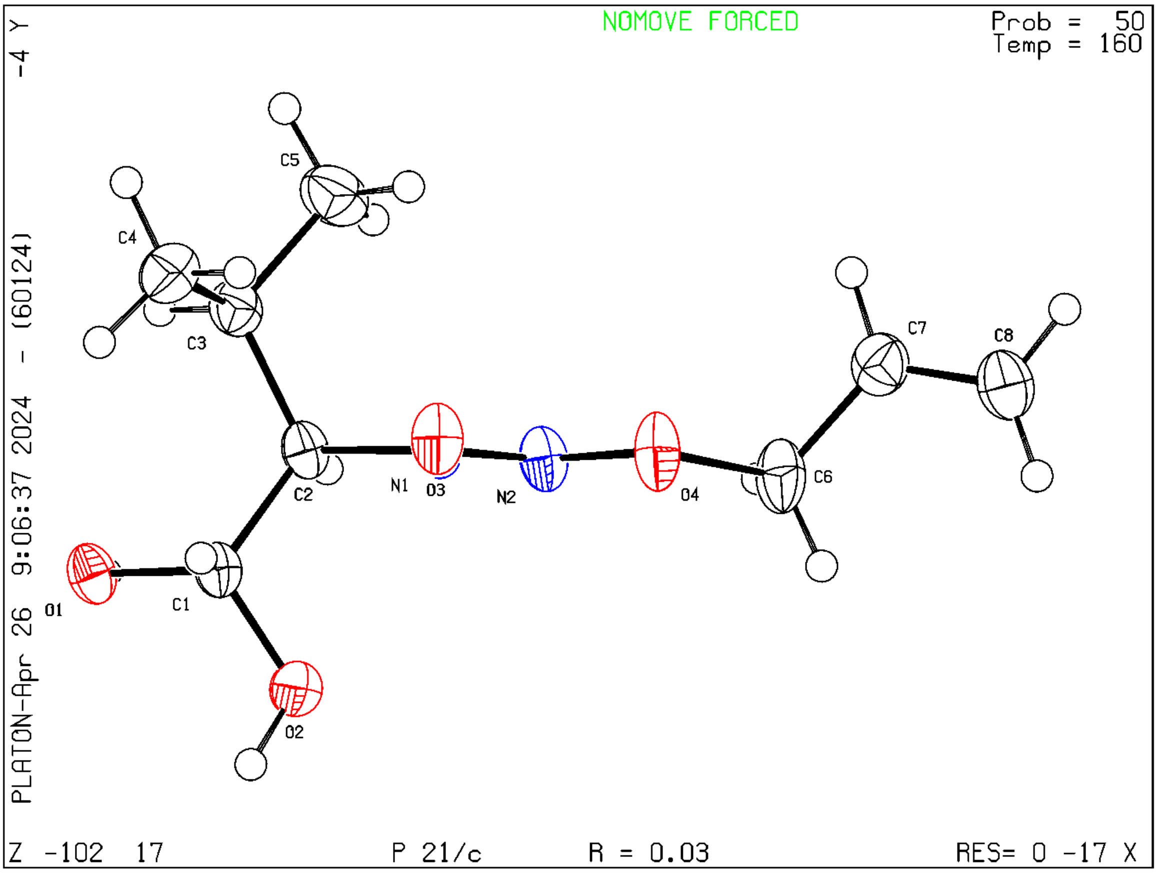

Supplement: Supplementary file 5 — Supplementary Data 3 [file 42004_2024_1372_MOESM5_ESM.pdf]
